# Ruthenium Assemblies for CO_2_ Reduction and H_2_ Generation: Time Resolved Infrared Spectroscopy, Spectroelectrochemistry and a Photocatalysis Study in Solution and on NiO

**DOI:** 10.3389/fchem.2021.795877

**Published:** 2021-12-24

**Authors:** Florian J. R. Cerpentier, Joshua Karlsson, Ralte Lalrempuia, Michael P. Brandon, Igor V. Sazanovich, Gregory M. Greetham, Elizabeth A. Gibson, Mary T. Pryce

**Affiliations:** ^1^ School of Chemical Sciences, Dublin City University, Dublin, Ireland; ^2^ Energy Materials Laboratory, Department of Chemistry, School of Natural and Environmental Science, Newcastle University, Newcastle upon Tyne, United Kingdom; ^3^ Department of Chemistry, School of Physical Sciences, Mizoram University, Aizawl, India; ^4^ Central Laser Facility, Science and Technology Facilities Council, Research Complex at Harwell, Rutherford Appleton Laboratory, Oxford, United Kingdom

**Keywords:** photocatalysis, hydrogen, CO_2_, time resolved spectroscopy, ruthenium assemblies, spectroelectrochemistry

## Abstract

Two novel supramolecular complexes **RuRe** ([Ru(dceb)_2_(bpt)Re(CO)_3_Cl](PF_6_)) and **RuPt** ([Ru(dceb)_2_(bpt)PtI(H_2_O)](PF_6_)_2_) [dceb = diethyl(2,2′-bipyridine)-4,4′-dicarboxylate, bpt = 3,5-di(pyridine-2-yl)-1,2,4-triazolate] were synthesized as new catalysts for photocatalytic CO_2_ reduction and H_2_ evolution, respectively. The influence of the catalytic metal for successful catalysis in solution and on a NiO semiconductor was examined. IR-active handles in the form of carbonyl groups on the peripheral ligand on the photosensitiser were used to study the excited states populated, as well as the one-electron reduced intermediate species using infrared and UV-Vis spectroelectrochemistry, and time resolved infrared spectroscopy. Inclusion of ethyl-ester moieties led to a reduction in the LUMO energies on the peripheral bipyridine ligand, resulting in localization of the ^3^MLCT excited state on these peripheral ligands following excitation. **RuPt** generated hydrogen in solution and when immobilized on NiO in a photoelectrochemical (PEC) cell. **RuRe** was inactive as a CO_2_ reduction catalyst in solution, and produced only trace amounts of CO when the photocatalyst was immobilized on NiO in a PEC cell saturated with CO_2_.

## Introduction

The transition to renewable energy has become a key global objective due to the dwindling supply of fossil fuels, as well as the detrimental effect they have on the environment. While sufficient renewable energy is available in the form of sunlight and wind, the intermittency of these sources remains a problem. Storage of excess energy captured from these sources into chemical bonds can potentially provide a viable solution (Cline and Bernhard, 2009). Nature captures sunlight by photosynthesis, and much effort has been made in our research group and others to mimic this process and create efficient systems for artificial photosynthesis (Tamaki et al., 2016; Kaufhold et al., 2017; Đokić and Soo, 2018; Dalle et al., 2019; Põldme et al., 2019; Toupance et al., 2019).

A typical catalytic system consists of two components: a photosensitizer (**PS**) which captures (visible) light and a catalyst capable of converting a low energy substrate, such as CO_2_ or water, into a higher energy vector, for example carbon fuels or hydrogen gas (Deponti and Natali, 2016; Cancelliere et al., 2020). Classical intermolecular systems have been reported, where both components are present in solution and interact with each other upon collision in a bimolecular process (Hansen et al., 2012). However, in order to improve the interaction between the **PS** and catalyst, and thus the rate of forward electron transfer, the two functionalities can be covalently bonded to each other (Ogawa et al., 2010; Ozawa et al., 2010; Kowacs et al., 2015; Kaeffer et al., 2016; O’Reilly et al., 2018). These intramolecular systems show that electron transfer from the **PS** to the catalyst is improved (Ozawa et al., 2006; Li et al., 2009; Ozawa et al., 2010), but these systems may suffer from rapid charge recombination. Ruthenium polypyridyl complexes have played a major role in the development of supramolecular systems, both for hydrogen evolution and CO_2_ reduction. For the former, the groups of Rau and Dietzek (Tschierlei et al., 2010; Zedler et al., 2014; Mengele et al., 2016) and the group of Sakai (Ozawa et al., 2006; Yamauchi et al., 2009; Ozawa et al., 2010; Ozawa and Sakai, 2011), as well as our own (Singh Bindra et al., 2011; Rau et al., 2013; Kowacs et al., 2016; O’Reilly et al., 2018), have reported a large range of dinuclear complexes in which the catalytic metals, as well as bridging ligand and peripheral ligands, were varied and the effects investigated.

**FIGURE 1:**
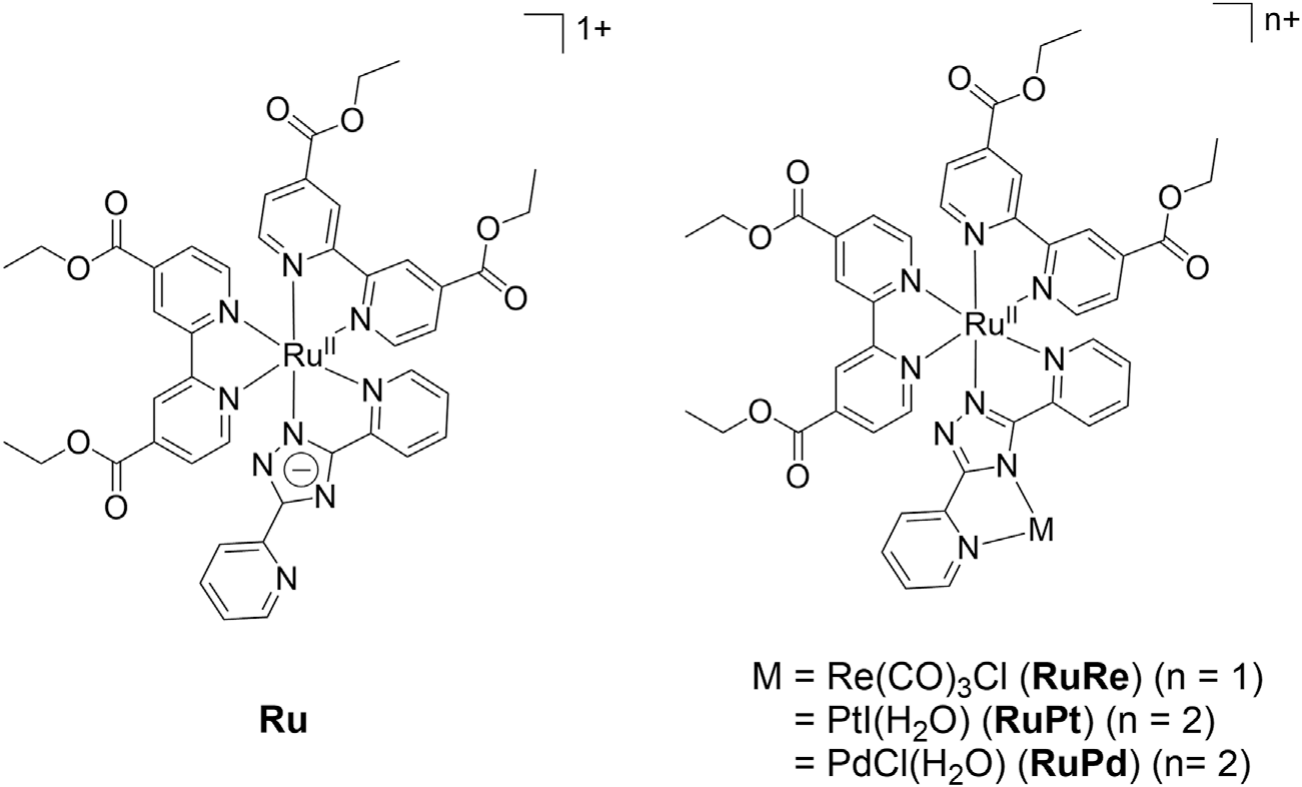
The complexes studied in this paper and the previously reported (Põldme et al., 2019) RuPd complex.

We and others have shown the importance of the energy levels of the peripheral and bridging ligand (Tschierlei et al., 2010), as directional electron transfer from the ruthenium centre via the bridging ligand to the catalytic centre is required to facilitate catalysis (Tschierlei et al., 2010; Karnahl et al., 2011; Singh Bindra et al., 2012; Zedler et al., 2019). For CO_2_ reduction, the group of Ishitani has reported various suitable systems, showcasing the influence of the bridging ligand in particular on the catalysis (Sato et al., 2007; Nakada et al., 2015; Ueda et al., 2015; Tamaki and Ishitani, 2017). In their systems, it was apparent that catalysis was enhanced if the bridging ligand was not conjugated, minimizing the electronic interaction between the ruthenium and the rhenium centres (Gholamkhass et al., 2005; Bian et al., 2008; Bian et al., 2009; Yamazaki et al., 2019). They reported that stronger conjugation led to a weaker reduction potential of the rhenium unit, and decreased the efficiency of the catalyst to reduce CO_2_ (Gholamkhass et al., 2005). For large scale application, photoelectrochemical cells where the reductive reactions are coupled with a suitable oxidative reaction which can provide electrons are required. (Yu et al., 2015; Hoogeveen et al., 2016; Morita et al., 2017). A preferred way to do so is by attaching the system for proton reduction to a semiconductor cathode, while a water oxidation system is coupled to a semiconductor anode, facilitating the electron flow (Kamata et al., 2021). For this, the **PS** requires suitable anchoring groups to be attached to a semiconductor surface (Tamaki and Ishitani, 2017; Kamata et al., 2019; Materna et al., 2020). We have previously shown that these anchoring groups affect the energy levels of the peripheral ligands (Kowacs et al., 2016). In the case of the ester anchoring groups employed by us, they lower the triplet metal to ligand charge transfer (^3^MLCT) excited state localized on the peripheral ligand resulting in increased electron density on the peripheral ligands in the excited state. While this reduces electron transfer to the metal centre *via* the bridging ligand (Tschierlei et al., 2010; Karnahl et al., 2011; Singh Bindra et al., 2012; Zedler et al., 2019), it might also aid in storing the two reductive equivalents required for catalysis (Pan et al., 2016; Põldme et al., 2019). As previously reported by our groups (Põldme et al., 2019), the interaction with the semiconductor drastically influences the properties of these supramolecular systems. In our case, the ruthenium-palladium system **RuPd** was inactive in solution but was active for hydrogen production when immobilized on a NiO semiconductor surface through a shift in the energy levels of the peripheral and bridging ligands. Transient absorption spectroscopy (TAS) indicated that when the **RuPd** sample was integrated on the NiO semiconductor, rapid hole injection occurred following excitation, however the reduced species undergoes charge recombination within 30 ps. Furthermore surface based TAS studies indicated that the electron density is localized on the bridging ligand. This is in contrast to the studies in solution, where the excited state was mainly localised on the peripheral ligands. Previous reports have shown that supramolecular systems with either palladium or platinum as the hydrogen evolution catalyst perform catalysis *via* different mechanisms (Pfeffer et al., 2015). For systems using palladium, partial decomposition of the supramolecular assembly has been observed, resulting in the release of palladium from the supramolecular assemblies to form palladium colloids which are thought to act as catalysts (Lei et al., 2008; Pfeffer et al., 2015; Staniszewska et al., 2019). In the case of platinum, it remained coordinated to the bridging ligand during photocatalysis. For catalysis to occur, efficient electron transfer towards the metal centre *via* the bridging ligand is required (Tschierlei et al., 2010; Karnahl et al., 2011; Singh Bindra et al., 2012; Pfeffer et al., 2015; Zedler et al., 2019). We synthesized **RuRe** and **RuPt** complexes as the rhenium and platinum analogues of **RuPd**, to investigate the influence of the catalytic metal on the excited state properties and the catalytic properties of these systems, not only in solution but when integrated onto NiO (Figure 1).

Infrared and UV-Vis spectroelectrochemistry, as well as steady state photophysical methods, time-resolved infrared spectroscopy and time-resolved emission spectroscopy were used to probe and compare the properties of these complexes. The mononuclear photosensitizer **Ru** was used as a benchmark in these studies. Spectroelectrochemistry has been a valuable tool in the analysis of ruthenium complexes and their bimetallic supramolecular systems. Specifically UV-Vis and Raman spectroelectrochemistry have been widely applied to study the localization of the electron in the one electron reduced species (Zedler et al., 2014; Zhang et al., 2015; Zhang et al., 2018). This species is considered an intermediate in the catalytic cycle to produce hydrogen and thus a better understanding of its properties is of interest (Zedler et al., 2014). UV-Vis spectroelectrochemistry can indicate the electronic transitions involved in the localization of the first light-induced electron transfer and the transition involved in the second excitation. However, various states often overlap making their assignment difficult, generally requiring complementary computational chemistry to interpret the spectra (Zedler et al., 2014). Raman spectroelectrochemistry shows more detail and can use the various electronic transitions to focus on different aspects of the molecule. In order to analyse the various signals and assign them to the various, often very similar parts of the complexes, benchmarks or additional computational studies are required (Zedler et al., 2014; Zhang et al., 2015; Kranz and Wächtler, 2021). Infrared spectroelectrochemistry can complement these techniques. The presence of IR-active functional groups, such as carboxyl moieties, can make interpretation of the changes occurring following reduction of the complex easier, as these functional groups have unique signals in the IR spectrum. In our complex, we use the organic carbonyl groups on the peripheral ligands, as well as the metal-carbonyl groups on the rhenium catalyst to investigate the localization of the electron after the first reduction. Finally, time-resolved infrared spectroscopy (TRIR) on the picosecond and nanosecond timescale was used to study the excited state of the complexes. TRIR has been a widely applied tool to study ruthenium based supramolecular systems, in particular for systems containing metal-carbonyl moieties such as Re(CO)_3_X (X = halide), as the metal carbonyls at the catalytic site are valuable handles in the IR spectrum (Bian et al., 2009; Frayne et al., 2018; Kamata et al., 2019; Põldme et al., 2019; Yamazaki et al., 2019). The metal-carbonyl moieties are mostly influenced through effects on the bridging ligand, while the organic carbonyls are mostly influenced by effects on the peripheral ligands. This helped us to assign the localization of the excited states following excitation.

## Results and Discussion

### UV-Vis Spectroscopy and Emission Spectra

**FIGURE 2:**
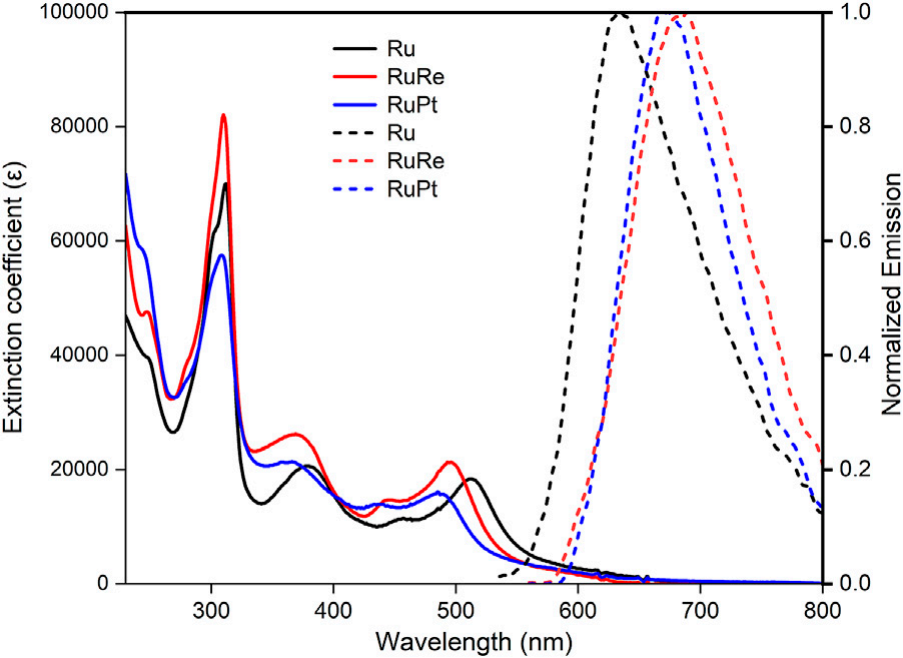
UV-Vis absorption of Ru (black), RuRe (red) RuPt (blue) and normalized emission spectra of Ru (dashed black), RuRe (dashed red), RuPt (dashed blue). UV-Vis spectra were collected using a 0.025 mM solution of the complexes in aerated acetonitrile in a 1 cm path length cell. Emission spectra were collected in a 1 cm pathlength cell at 490 nm excitation with all complexes having an absorbance of 0.1 at 490 nm.

The UV-vis absorption and emission spectra for **Ru**, **RuRe,** and **RuPt** are shown in Figure 2 and summarized in Table 1. Absorptions bands are observed at 312 and 381 nm for Ru and are assigned to ligand centred (LC) π-π* transitions on the bridging and peripheral ligands (Isakov et al., 2019). The broad absorption with a λ_max_ at 505 nm as well as the slight shoulder at 455 nm are assigned to metal-to-ligand charge transfer (MLCT) transitions from the Ru metal centre to the ligands (Isakov et al., 2019; Põldme et al., 2019). The absorption spectra of both **RuRe** and **RuPt** resemble that of **Ru**, showing two similar LC π-π* transitions in the UV region of the spectrum. A slight blue shift is observed for the MLCT transition for both dinuclear species following complexation with the second metal (Re or Pt) when compared to the **Ru** precursor, with a shift of the λ_max_ from 505 nm for **Ru** to 492 and 486 nm for **RuRe** and **RuPt**, respectively. This indicates some electronic coupling via the bridging ligand between the ruthenium centre and the second metal centre.

**TABLE 1:** Summarized absorption and emission maxima and emission lifetimes in aerated acetonitrile.

Compound	λ_max_ (nm)	*ε* x 10^4^ (M^−1^ cm^−1^)^a^	λ_em_ (nm)^b^	Stokes shift (cm^−1^)	τ (ns)^d^
**Ru**	312, 381, 455, 505	1.38	639	3,871	94, 1,294
**RuRe**	310, 374, 441, 492	2.06	697	5,727	557
**RuPt**	309, 376, 439, 486	1.57	682	5,914	14, 517

a Exctinction coefficient at λ_max_ for the MLCT band.

b Emission spectra were collected following excitation at 490 nm and all samples were isoabsorptive (0.1 A).

c The UV-Vis spectra for these species can be found in Figure 2.

d Lifetimes were determined at 690 nm following excitation at 355 nm.

The emission spectra further demonstrate the influence of the additional metal centres. The emission maximum for Ru was observed at 639 nm, which is in good agreement with values reported for related compounds (Pan et al., 2016; Frayne et al., 2018). Complexation with the second metal results in a red shift in emission, with maxima at 697 and 682 nm for RuRe and RuPt, respectively. A similar red shift in emission properties was previously observed for analogous complexes (Das et al., 2017). A significant change is observed in the Stokes shift which increases from 3,871 cm^−1^ for Ru to 5,727 and 5,914 cm^−1^ for RuRe and RuPt, respectively. This large increase indicates that the energy of the excited states is influenced upon coordination of a second metal.

### Electrochemistry

All complexes were studied using standard cyclic voltammetry techniques in acetonitrile with 0.1 M tetrabutylammonium hexafluorophosphate as supporting electrolyte. The potentials are quoted versus the ferrocene/ferrocenium redox couple unless otherwise noted and can be found in Table 2. **Ru** is studied as a benchmark for the dinuclear species **RuPt** and **RuRe**, as well as providing a comparison to other ruthenium photosensitizers in literature. As with other heteroleptic ruthenium polypyridine complexes, three reversible reductions are observed, which can be assigned to the individual reductions of each of the three polypyridyl ligands (Hage et al., 1991; Frayne et al., 2018) (Supplementary Figure S1). Using infrared spectroelectrochemistry (*vide infra*) the reductions are assigned as follows: the first two waves, with closely spaced E_1/2_ potentials at −1.41 and −1.54 V are assigned to reductions on the dceb ligands. The third reduction, with a characteristic E_1/2_ value of −2.12 V, is assigned to the reduction of the triazole bridging ligand. This value matches well with that previously observed for a similar ruthenium complex bearing the same triazole ligand studied by Mulhern et al., who reported a reduction on the triazole ligand at −2.16 V (Mulhern et al., 2006). One reversible oxidation was observed at 0.68 V which is assigned to the reversible Ru^II/III^ oxidation. A second shouldering oxidation feature was observed at 0.81 V which was confirmed with differential pulse voltammetry (Supplementary Figure S2). In previous work reported on the analogues [Ru(bpy)_2_(bpt)]^+^ complex (bpy = 2,2′-bipyridine) by Browne et al. (2002), a positive shift of the oxidation potential was observed upon protonation of the triazole ligand, prompting us to investigate if these two oxidations could be assigned to the protonated and deprotonated species in solution. CV experiments in the presence of 0.02 M NaOH resulted in the disappearance of the oxidation band at 0.81 V, confirming that this oxidation is indeed that for the protonated species (Supplementary Figure S3). The reductive processes were not affected by the addition of base to the solution, suggesting that these signals should be assigned to the deprotonated species.

**TABLE 2:** Reduction and oxidation potentials of the reported ruthenium complexes in nitrogen purged acetonitrile with 0.1 M TBAPF_6_ as supporting electrolyte. All potentials are versus the Fc/Fc^+^ reference. i = irreversible process.

Compound	E_1/2_ (V)	E_1/2_ (V)	E_1/2_ (V)	E_1/2_ (V)	E (V)
**Ru**	−1.41	−1.54	−2.12	0.68 (Ru)	—
**RuRe**	−1.39	−1.61	−2.11	0.87 (Ru)	1.12 (i) (Re)
**RuPt**	−1.38	−1.62	−2.16 (i)	0.98 (i) (Ru)	1.22 (i)

**FIGURE 3:**
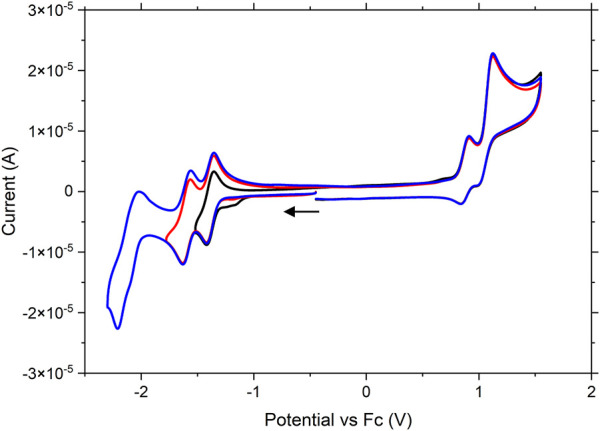
Cyclic voltammogram of RuRe vs ferrocene. Experiments were run with 1 mM RuRe in nitrogen purged acetonitrile with 0.1 M tetrabutylammonium hexafluorophosphate as supporting electrolyte. A glassy carbon electrode, platinum wire and Ag/AgNO_3_ electrode were used as working, counter and reference electrodes, respectively. Scans were taken at 0.1 V/s.

**RuRe** showcased electrochemical properties very similar to those observed for **Ru** (Figure 3). Three reversible reductions were evident with E_1/2_ potentials at −1.39, −1.61, and −2.11 V. A reversible oxidation was observed with an E_1/2_ potential of 0.87 V which was assigned to the Ru^II/III^ oxidation. This oxidation has shifted to a more positive potential (0.87 V vs. 0.68 V for **Ru**). As previously discussed for **Ru**, protonation of the triazole unit has been shown to effect such an anodic shift in the Ru^II^ oxidation potential. It is therefore not unreasonable to postulate that the observed increase in potential for Ru^II/III^ couple arises due to the coordination of the Re centre to the triazole bridge, which helps to neutralize negative charge on this ligand. A second irreversible oxidation is observed at 1.12 V which is assigned to the Re^I/II^ oxidation. This value matches well with the Ru-Re quaterpyridine analogue Ru (dceb)_2_(L)Re(CO)_3_Cl (L = 2,2′,5′,3″,6″,2‴-quaterpyridine) previously reported by Frayne et al. (2018), who observed an irreversible oxidation assigned to the Re^I/II^ couple at 1.07 V. The profile of this peak is reminiscent of a coupled electrochemical-chemical process in which the oxidation product is consumed by an irreversible chemical (decomposition) reaction. When the experiment was conducted at a scan rate of 0.1 V.s^−1^, a small inflection was observed at 1.00 V on the return scan following the oxidation at 1.12 V. This inflection is assigned to the re-reduction of a residual amount of the 1.12 V oxidation product that has not decomposed. When the time-scale of the experiment was increased by changing the scan rate to 0.01 V.s^−1^ (Supplementary Figure S4), more complete chemical decomposition of the oxidation product occurs and consequently the inflection feature becomes less prominent. This observation coincides with the results of oxidative spectroelectrochemistry experiments (*vide infra*) where a significant loss of signal for the metal carbonyls was noted following oxidation, suggesting that decomposition may follow directly upon the oxidation of the Re moiety.

For **RuPt** slightly different behaviour was observed (Supplementary Figure S5). Three reductions were observed at −1.38, −1.62, and −2.16 V, similar to those reported for **Ru** and **RuRe**. However, reversibility was partially lost for the first two reductions and completely lost for the third reduction. By analogy to the potentials observed for **Ru** and **RuRe**, these reductions are assigned to take place on the peripheral ligands and bridging ligand, respectively. However, a possible Pt centred reduction overlapping with the ligand centred reduction at −1.62 V cannot be ruled out. Two irreversible oxidations were observed at 0.98 and 1.22 V. The first oxidation at 0.98 V is tentatively assigned to the Ru^II/III^ oxidation. This oxidation has shifted anodically 300 mV when compared to **Ru**. We are currently unclear about the origin of the rather broad anodic current peak at 1.22 V, as similar oxidative features have not been observed for other Ru-Pt dinuclear species reported in the literature (Kobayashi et al., 2010; Ogawa et al., 2011; Miyaji et al., 2017).

### Spectroelectrochemistry

Infrared and UV-Vis spectroelectrochemistry were used to gain a better understanding of the potential intermediates generated during photocatalysis. IR-SEC can help to determine localization of the electrons during photocatalysis and aid in determining the mechanisms, as well as optimizing ligand and complex design. In the case of the **RuRe** complex, the metal-carbonyls on the rhenium centre provide an extra handle to monitor changes in electron density at the metal centre.

#### Infrared Spectroelectrochemistry

Initially, the **RuRe** complex was studied and carbonyls of the ester functional groups, as well as the metal carbonyls on the rhenium centre are used as a handle to determine the localization of the electrons during electrochemical reduction.

**FIGURE 4:**
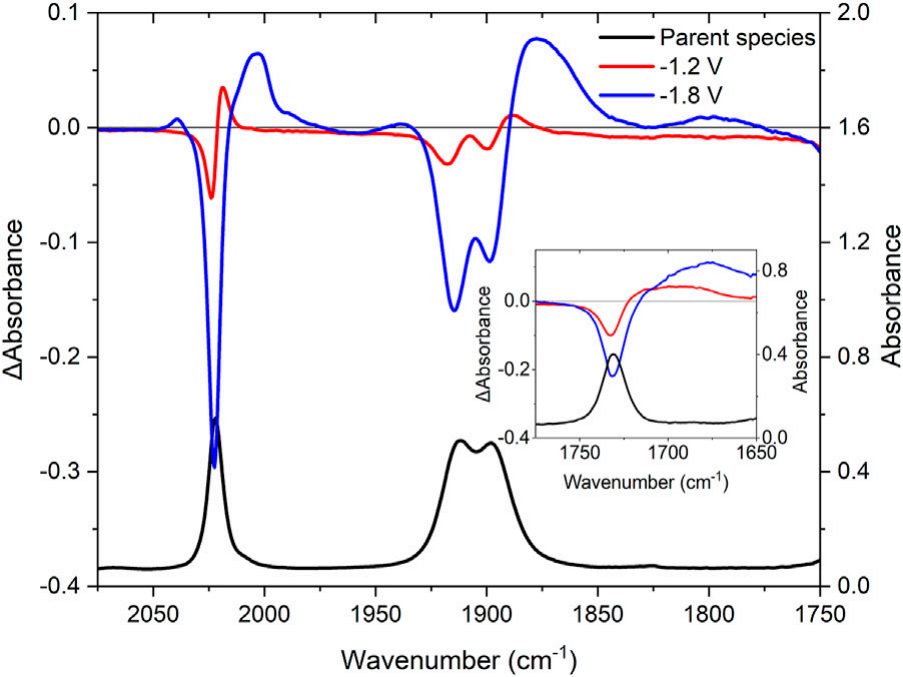
IR-SEC spectra for **RuRe** in acetonitrile with 0.1 M TBAPF_6_ as supporting electrolyte. The IR (black) and difference spectra at −1.2 V (red) and −1.8 V (blue) vs. silver wire pseudo-reference.

The IR spectrum of **RuRe** has metal carbonyl stretches at 2,022, 1,913, and 1,899 cm^−1^, as expected for a *fac*-tris carbonyl rhenium species, together with the organic carbonyl band at 1,730 cm^−1^ (Figure 4, black trace). Upon the first 1-electron reduction starting at −1.2 V *vs* the Ag/Ag^+^ pseudo-electrode (Figure 4, red trace), bleaching of the organic carbonyl at 1,730 cm^−1^ occurs, with the formation of a broad absorption ranging from 1,712 to 1,656 cm^−1^. This suggests localization of the electron on the peripheral ligand resulting in weakening of the C-O bond, with shifting of the stretching vibration to lower energy by approximately 40 cm^−1^. The Re-carbonyl stretching vibrations also shift slightly to lower energy from 2,022, 1,913 and 1,899 to 2,018, 1,905 and 1,885 cm^−1^. This shift is smaller than that observed for the organic carbonyls, suggesting that the reduction takes place on the peripheral ligands and not on the bridging ligand.

As the reduction potential was slowly decreased, the second reduction was reached at −1.4 V vs the Ag/Ag^+^ couple and the intensity of the new signals for the organic carbonyl between 1,712 and 1,656 cm^−1^ increased, while the signals in the metal carbonyl region at 2,018, 1,905 and 1,885 cm^−1^ further depleted. This indicates that the reduction of the second peripheral ligand occurs prior to the reduction of the bridging ligand as suggested by the electrochemical data (*vide supra*). A third reduction occurs at −1.8 V vs. the Ag/Ag^+^ couple, leading to the changes shown in Figure 4 (blue trace). Initially, the bleach at 2,022 cm^−1^ increases and the new signal at 2,018 cm^−1^ disappears with a new broad band at ∼2,000 cm^−1^. The bleaches at 1,913 and 1,899 deplete further, together with the formation of a new broad band at 1,880 cm^−1^. The similarity of the new signals to the neutral complex suggest that the reduction takes place on the bridging ligand and not at the rhenium centre, as reduction of the rhenium metal would result in the dissociation of the halide and subsequent reactions (Stor et al., 1995; Frantz et al., 2004; Záliš et al., 2011).

Oxidation of **RuRe** at 2 V vs. the Ag/Ag^+^ couple leads to bleaching of the metal carbonyl signals at 2,022, 1,913, and 1,899 cm^−1^ (Supplementary Figure S9) together with bleaching of the organic carbonyl signal at 1,730 cm^−1^. New Re-CO carbonyl stretching vibrations were observed at higher energy, at 2,031 and 1,932 cm^−1^ (albeit very weak in intensity). This may suggest possible loss of the metal carbonyl moieties under oxidative conditions. A stronger new signal is observed for the organic carbonyl at 1,740 cm^−1^ indicating a decrease of electron density on the peripheral ligand. Oxidation on the ruthenium centre induces an electron withdrawing effect on the peripheral ligands leading to a lowering in electron density on the ligand and a shift in the carbonyl bands to higher energy. A similar but weaker effect is observed for the metal carbonyls.

#### UV-Vis Spectroelectrochemistry

**FIGURE 5:**
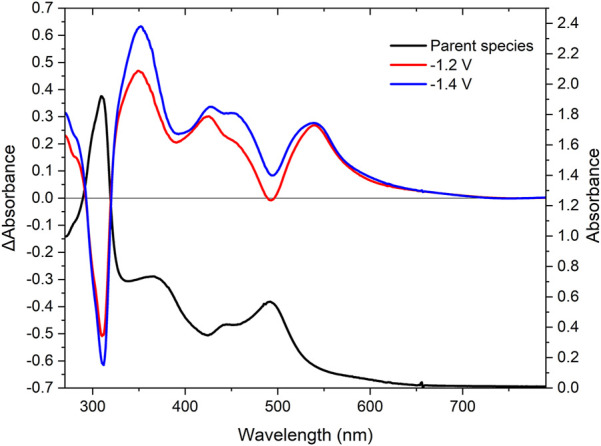
UV-Vis SEC spectra of RuRe in acetonitrile with 0.1 M TBAPF_6_. The UV-Vis spectrum (black) and difference spectra at −1.2 V (red) and −1.4 V (blue) *vs* the silver wire pseudo-reference.

Reductive UV-SEC was also carried out using **RuRe** with the changes observed depicted in Figure 5. Upon the first one electron reduction at −1.2 V vs. the Ag/Ag^+^ couple, the ligand centred transitions at 310 nm and the MLCT bands at 441 and 492 nm are bleached. New ligand centred transitions appear at 276 and 352 nm, in addition to new absorptions in the visible region at 444 and 548 nm. The new band at 444 nm is assigned to a ligand-to-metal charge transfer (LMCT) based on the literature (Põldme et al., 2019; Rupp et al., 2019). The new band at 548 nm is assigned to a ligand centred π-π* transition (Zedler et al., 2014; O’Reilly et al., 2018; Põldme et al., 2019). Changing the potential to include the second reversible reduction at −1.4 V resulted in a further increase of intensity for the signals observed after the first reduction (Figure 5, blue trace), further supporting the assignment of this reduction on the second peripheral ligand. Probing of the third irreversible reduction was attempted. However, interference from solvent loss prevented us from studying the changes in the absorbance spectra following the third electron reduction.

The oxidative UV-Vis spectroelectrochemistry of **RuRe** is presented in the **ESI** in Supplementary Figure S17. At the onset of the first reversible oxidation of the ruthenium centre at 1.4 V vs. the Ag/Ag^+^ couple, the ligand-centred transition at 310 nm redshifts slightly to 332 nm, through an inductive effect of the more positively charged ruthenium metal centre. The bands at 441 and 492 nm are bleached. As an electron is removed from the ruthenium centre, insufficient electron density is present on the metal centre to facilitate a MLCT. A broad but weak absorption between 650 and 1,080 nm is observed. This absorption is assigned to a new LMCT from the polypyridyl ligands to the ruthenium centre (Zhang et al., 2018).

### Time-Resolved Infrared Spectroscopy

**FIGURE 6:**
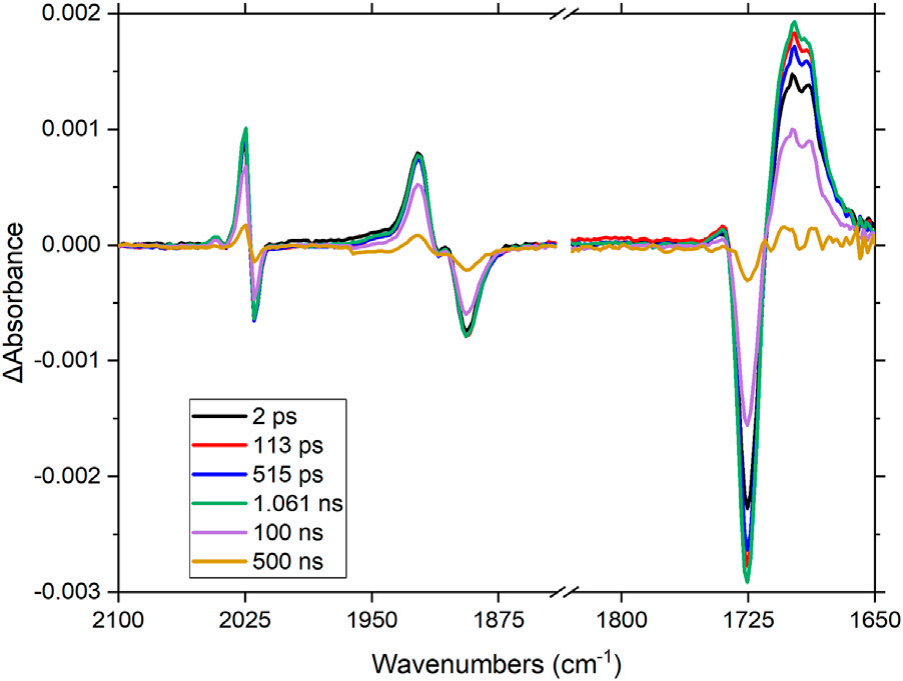
Time-resolved infrared spectrum following excitation of RuRe in CH_3_CN-d_3_ at 510 nm (1 μJ). Note-the spectra were collected using two separate solutions, indicated by the dash in the axis.

Time-resolved infrared spectroscopy (TRIR) was used to investigate the excited state properties of the dinuclear complexes. Insight into the localization and lifetimes of the excited states is important to further improve materials for either hydrogen generation or CO_2_ reduction (Tschierlei et al., 2009; Koike et al., 2018; Yamazaki et al., 2019). The TRIR spectra for **RuRe** following excitation at 510 nm are shown in Figure 6.

Upon excitation at 510 nm, the Re-CO stretching vibrations at 1,892, 1,911, and 2,019 cm^−1^ deplete (Figure 6), together with the organic carbonyl (1,730 cm^−1^) on the peripheral ligands. Four new absorptions in this region are observed within the pulse; a broad signal at 1,708 cm^−1^ and three sharper signals at 1,904, 1,922 (overlaps with parent depletion) and 2,024 cm^−1^ (there is another feature to the high energy side of this latter band, the origin of which is unclear). These signals decay within 500 ns with concomitant recovery of the parent species, in non-degassed acetonitrile. The shift of the organic carbonyl to lower energy suggests that charge transfer takes place from the ruthenium centre towards the peripheral ligands. The shift of the metal-carbonyl signals to higher energy suggests that electron density decreases on the rhenium centre, thus ruling out electron transfer occurring from the ruthenium centre via the bridging ligand towards the rhenium metal centre, following excitation at 510 nm. These spectral changes agree with the previous observations in the electrochemical and spectroelectrochemical results (*vide supra*), were the LUMO was assigned to be localized on the peripheral dceb ligand with reduction on the bridging ligand only occurring at much lower potentials.

The TRIR data for **RuPt** is shown in the **ESI** (Supplementary Figure S21). As for **RuRe**, the organic carbonyl from the ester moiety on the peripheral ligands in **RuPt** bleaches at 1,729 cm^−1^ following excitation at 510 nm. A new absorption at approximately 1,700 cm^−1^ is observed, again indicating an increase in electron density on the peripheral ligands, as was similarly observed for **RuRe**. This signal decays over 500 ns with concomitant recovery of the parent species.

### Emission Lifetimes

Emission lifetimes were determined at 690 nm following excitation at 355 nm (Supplementary Figures S22–S24). The three complexes were studied in deoxygenated acetonitrile solutions. For the mononuclear **Ru** species, a biexponential decay with lifetimes of 94 and 1,284 ns was observed. The longer lifetime corresponds well to those found for similar ruthenium complexes (Hage et al., 1991). The origin of the shorter lifetime is currently unclear. For the **RuRe** dinuclear complex a single lifetime of 557 ns was observed which was assigned to the ^3^MLCT state, and is significantly shorter than that of the mononuculear analogue. For the **RuPt** complex a biexponential decay with lifetimes of 14 and 517 ns was observed. In other dinuclear species, electron transfer towards the second metal resulted in a decrease of the lifetimes (Rau et al., 2013; Pan et al., 2014; Das et al., 2017; O’Reilly et al., 2018). However, the TRIR data did not indicate any electron transfer from the ruthenium centre towards the second metal centre for **RuRe**. Instead the coordination of the Re and Pt metal centres alters the energy levels of the ground and excited states, as observed in the shift in the UV-Vis spectra and emission spectra between the mononuclear **Ru** and the dinuclear **RuRe** and **RuPt** (*vide supra*).

### Photocatalysis in Solution

**RuPt** was assessed for photocatalytic hydrogen evolution in an acetonitrile:triethylamine:water (60:30:10) mixture, using a 470 nm LED. A time dependent study over 24 h showed no induction period for hydrogen generation (Supplementary Figure S25) with a TON of 81 at 24 h. In addition, TOFs of up to 20 h^−1^ were observed in the early stage of the catalytic reaction, which decreased notably within 10 h. Absorption spectra obtained before and after photocatalysis experiments (Supplementary Figure S26) indicates decomposition which correlated with the TONs observed for **RuPt**. The formation of a black precipitate after 10 h further supports this assignment. Under identical conditions no hydrogen was detected for the previously reported palladium analogue (**RuPd**). However, other Ru-Pt dincluear systems, for example Ru (tbbpy)_2_ (tpphz)PtI_2_ [tbbpy = 4,4′-di-tert-butyl-2,2′-bipyridine, tpphz = tetrapyrido(3,2-a:2′,3′-c:3″,2″-h:2‴,3‴-j)phenazine] reported by Pfeffer et al. reported TONs of up to 238 under similar experimental conditions. Another ruthenium complex, Ru (dceb)_2_(2,5-dpp)PtCl(H_2_O) (dpp = 2,2′; 5′,2″-terpyiridine) which contains the same peripheral ligands but a terpyridine bridging ligand also yielded much higher TONs of up to 400 after 6 h of irradiation. This indicates that the bpt bridging ligand limits electron transfer towards the platinum centre following excitation and reductive quenching.

**RuRe** was assessed for photocatalytic CO_2_ reduction in a 5:1 DMF:TEOA solution (purged with CO_2_) with 1,000 equivalents (vs. catalyst) of BIH as a sacrificial agent, and 470 nm irradiation. However, no CO was detected in solution after 24 h of irradiation.

### Photoelectrocatalysis on NiO

**RuPt** and **RuRe** were also tested for photoelectrocatalytic water-splitting and CO_2_ reduction respectively when immobilized on a p-type semiconductor (NiO) in a small-scale photoelectrochemical (PEC) test device. Photoelectrochemical experiments were performed in a gas-tight three electrode glass cell with the photocatalyst immobilized on a thin film of mesoporous NiO. In this setup the counter electrode was a platinum wire and the reference Ag/AgCl (3 M KCl). Mesoporous NiO thin films of approximately 1.5 micron thickness were produced by doctor blading NiO precursor solution onto conductive glass substrates (Pilkington TEC 15 fluorine doped tin oxide glass), followed by annealing at 450°C for 30 min with a ramp time of 30 min. Full details of the method for preparing a NiO precursor solution can be found in the experimental section. The assembled PEC cell was filled with the desired aqueous electrolyte and purged for 20 min with research grade CO_2_ (BOC) or argon before an experiment. The electrodes were connected to a potentiostat (Palmsens EmStat3 Blue) and illumination was provided by a 300 W Xe arc lamp. The distance from the lamp to the face of the photocathode was adjusted so that illumination intensity was equivalent to one Sun (100 mW cm^−2^). An AM 1.5 filter was used with the light source to simulate the solar spectrum.

We and others showed previously (Põldme et al., 2019) that the ethyl ester group is an effective means of adsorption of the photocatalyst to the NiO surface, as it facilitates photoinduced charge transfer at the interface and has an impact on tuning the energy and dynamics of the excited states.

With the photocatalysts immobilized on the NiO following dye bath (3 mM concentration of dye in dry acetonitrile) deposition for 12 h, the NiO was visibly coloured. UV-vis spectroscopy revealed that the absorption maxima for **RuPt** and **RuRe** had red-shifted, the absorption maximum for the MLCT band now residing at 575 for **Ru**, 520 for **RuPt** and 537 nm for **RuRe** (Supplementary Figure S28). Similar broadening and red-shifting of the spectra is consistent with previous studies with similar photocatalysts on NiO (Põldme et al., 2019).

Dye adsorption was quantified by UV-vis absorption measurements of solutions following dye desorption from the sensitized NiO films after soaking in 0.5 M NaOH solution. Using this method the approximate dye loading of the systems on NiO was 9 nmol cm^−2^ for **Ru**, 7 nmol cm^−2^ for **RuRe** and 4 nmol cm^−2^ for **RuPt**. Poldme et al. found 9 nmol cm^−2^ for **RuPd.** These values are similar in magnitude to other systems with typical anchoring groups such as phosphonic acid and carboxylic acid (Pellegrin et al., 2011; Wahyuono et al., 2020).

Photoelectrocatalysis was performed in various aqueous buffer solutions for **RuRe,** at pH 5, 8, and 9.2. The aim was to optimize device operating conditions such as the maximum and most stable photocurrent, as well as the detection of gaseous products. Table 3 provides a summary of electrochemical measurements in our PEC cell. In the case of **RuRe** photocurrent densities were consistently 1 μA cm^−2^ in pH 5 acetate buffer with an applied bias of −0.2 V, but this was improved in more alkaline conditions, with an average current density of 5.0 μA cm^−2^ in pH 9.2 carbonate buffer. Linear sweep voltammetry was used to determine an appropriate bias to be applied to the system without degrading the photocatalyst. Optimum performance was seen with an applied bias of −0.2 V in the carbonate buffer, where minimal surface charging of the NiO was observed. The systems were also tested with an applied bias of −0.5 V (see supporting information).

**TABLE 3:** **(A)** Photoelectrochemical measurement of mesoporous NiO films containing the photocatalyst RuRe. **(B)** Photoelectrochemical measurement of mesoporous NiO films containing the photocatalyst RuPt. Values are an average photocurrent across a measurement spanning 15 min. The stated applied bias is vs Ag/AgCl.

RuRe photocurrent density (μA cm^−2^)	pH 5 acetate buffer	pH 8 phosphate buffer	pH 9.2 carbonate buffer
−0.2 V	1.3	1.0	4.5
−0.5 V	1.1	2.5	5.0

RuPt photocurrent density (μA cm^−2^)	pH 5 acetate buffer	pH 8 phosphate buffer
−0.2 V	2.2	1.3
−0.5 V	7.9	7.4

Control experiments consisted of measuring blank NiO films and a NiO film with the compound lacking the catalytic centre (**Ru**). For a bare NiO film in pH 5 buffer with an applied bias of −0.2 V, light response was far less than with any of the dyed films, with an average photocurrent density of approx. 0.3 μA cm^−2^ recorded. Under the same conditions we recorded a photocurrent density of 1.3 μA cm^−2^ for **RuRe**. The experiments confirm that in general the performance of these photocatalysts is quite poor in acidic media, but there is evidence to suggest that more alkali conditions improve the performance. Gas chromatography measurement by head space sampling showed trace amounts of carbon monoxide being produced during photocatalysis of **RuRe** in pH 5 acetate buffer. After 1 h irradiation, ca. 25 nmols CO was recorded (Supplementary Figure S48 for GC trace) which is equivalent to a TON of 4.5. For **RuPt** we could not detect more than very small trace amounts of evolved hydrogen from the NiO surface. After 1 h, approximately 0.3 µmol H_2_ was recorded by GC. Photocurrent densities were similar to those of **RuRe** under similar conditions, but rapid dye desorption prevented measurements at higher pH.

## Conclusion

Two novel supramolecular photocatalysts **RuRe** and **RuPt** were synthesized for CO_2_ reduction and H_2_ evolution, respectively. The complexes showed comparable photophysical properties and also similar electrochemical properties were observed for the **RuRe** and the mononuclear benchmark **Ru** complex. Three reversible reductions were observed for **Ru** and **RuRe**, while for the **RuPt** analogue the reversibility of the third reduction was lost. The three reductions could be assigned to the various ligands using the spectroelectrochemical results. The first two reductions were found to take place on the peripheral ligands, while the third reduction takes place on the bridging ligand. Upon one electron reduction, the organic carbonyl stretch of the ester moiety on the peripheral ligand moves to lower energy, suggesting an increase in electron density on the peripheral ligand. A smaller shift of the metal carbonyls for **RuRe** was assigned to an inductive effect due to the overall increase in electron density. Time-resolved infrared spectroscopy on the picosecond to nanosecond timescale was used to probe the nature of the excited state. A shift to lower energy for the organic carbonyl with a concomitant shift of the metal-carbonyls to higher energy suggest a MLCT excited state that predominantly localizes on the peripheral ligands. **RuRe** was found to be inactive for photocatalytic CO_2_ reduction in DMF solution with BIH as sacrificial agent using 470 nm irradiation. The directionality of electron transfer towards the peripheral ligand, as observed in the TRIR experiment, may explain the inactivity of the photocatalyst. Additionally, the significantly more negative reduction potential of the bridging ligand at −2.11 V vs. Fc^0/+^, compared to the reduction potentials of the peripheral ligands at −1.39 and −1.61 V vs. Fc^0/+^, suggest a clear preference of the complex to store any photo accumulated electrons on the peripheral ligand, from where electron transfer to the rhenium centre does not occur. In contrast, **RuPt** showed activity for the generation of H_2_, with TONs of 81 after 24 h. This was quite surprising as the previously reported palladium analogue, **RuPd** did not show any activity in solution. The difference in mechanism between palladium and platinum complexes may account for this variation in results. When immobilized on a NiO surface and assembled in a photoelectrocatalysis cell, **RuPt** showed very little activity, unlike the previously reported **RuPd** analogue. This was surprising and suggests that the mechanism taking place in solution is different compared to the immobilized system. The photocurrent density was disappointing for both **RuRe** under CO_2_ and **RuPt** under Ar. It is likely that electron transfer to the peripheral ligand rather than the bridge favours charge-recombination at the interface between the NiO and the **Ru** photosensitizer rather than charge-transfer to the Re or Pt catalyst. Further work is necessary to optimize the relative energy of the components in the photocatalyst in order to improve the performance both in solution and in photocathodes.

## Experimental

### Materials

All chemicals and solvents were supplied by Aldrich Chemicals Co. and anhydrous solvents containing sure/seal were used under the flow of nitrogen.

### UV-Vis and Emission Spectroscopy

All UV spectra were recorded on an Agilent 8453 UV-vis spectrophotometer equipped with Agilent ChemStation software. Emission and excitation spectra were obtained on a Perkin Elmer LS 50B at 20 ± 1°C. UV-Vis spectra were taken at 0.025 mM concentration in aerated acetonitrile in a 1 cm pathlength cell. Emission spectra were measured at ∼0.01 mM with all complexes being iso-absorptive at 490 nm in a 1 cm pathlength fluorescence cuvette. All three complexes were excited at 490 nm.

### Fourier Transform Infrared Spectroscopy

FTIR measurements were carried out on Perkin-Elmer 2000 FTIR spectrophotometer in a liquid solution cell using spectrophotometric grade acetonitrile.

### Electrochemistry

All experiments were conducted with 1 mM solutions of the complex in spectroscopic grade acetonitrile with 0.1 M tetrabutylammonium hexafluorophosphate as supporting electrolyte. All solutions were purged for at least 10 min with N_2_. A glassy carbon electrode, platinum wire and Ag/AgNO_3_ electrode were used as working, counter and reference electrode, respectively. Ferrocene was added as internal reference. Scans were taken at 0.1 V/s.

### Spectroelectrochemistry

All experiments were conducted in a custom OTTLE cell supplied by Spectroelectrochemistry Reading with a platinum mesh working electrode, platinum mesh counter electrode and silver wire pseudo reference electrode. Fresh solutions of the complexes at 1.5 mM (UV-Vis) or 10 mM (IR) in spectroscopic grade acetonitrile with 0.1 M tetrabutylammonium hexafluorophosphate as supporting electrolyte were used. All solutions were purged for at least 10 min with N_2_.

### NMR Spectroscopy

All NMR spectra were recorded on either a Bruker 400 or 600 MHz spectrometer and were referenced to the deuterated solvent peak as an internal reference.

### Ps-Time Resolved Infrared Spectroscopy

ps-TRIR spectra were recorded using the ULTRA instrument at the Central Laser Facility in the Rutherford Appleton Laboratory in the United Kingdom and has been described elsewhere (Greetham et al., 2010). Spectra were collected using 510 nm excitation with 1 μJ power in a 0.1 mm pathlength Harrick cell.

### Time Resolved Emission Spectroscopy

Time-resolved photoluminescence was recorded using an Edinburgh Instruments LP980 Transient Absorption Spectrometer. The samples were prepared in anhydrous acetonitrile (Sigma-Aldrich, purity>99.9%) and degassed by three cycles of freeze-pump-thaw. The samples had a typical absorbance (in optical density OD) at the excitation wavelength (355 nm) equal to 0.1–0.15.

### Photocatalytic Homogeneous H_2_ Evolution and CO_2_ Reduction Experiments

For photocatalytic hydrogen evolution, each sample was prepared in a 23 ml glass Schlenk tube stoppered with an air-tight rubber septum. 0.01 mM catalyst was dissolved in a 6:3:1 mixture of acetonitrile:triethylamine:water. The solution was degassed using three freeze-pump-thaw cycles and placed under an N_2_ atmosphere prior to irradiation. An array with 470 nm LEDs with 20 mW output power was used to irradiate the sample. At selected timepoints, 1 ml of the headspace was withdrawn and injected into a GC to quantify the amount of hydrogen.

For photocatalytic CO_2_ reduction, each sample was prepared in a 23 ml glass Schlenk tube stoppered with an air-tight rubber septum. 0.01 mM catalyst was dissolved in a 5:1 N,N-dimethyl formamide:triethanolamine mixture with 0.01 M BIH (dimethylphenylbenzimidazoline) as sacrificial agent. The solution was degassed using three freeze-pump-thaw cycles prior to irradiation and subsequently purged with CO_2_ for 5 min. An array with 470 nm LEDs with 20 mW output power was used to irradiate the sample. At selected timepoints, 1 ml of the headspace was withdrawn and injected into a GC to quantify the amount of carbon monoxide.

### Photoelectrochemical Measurements on NiO

Photoelectrochemical measurements on dye-sensitized mesoporous NiO thin films were performed using the device illustrated in the supporting information, connected to a PalmSens EMStat3 Blue USB potentiostat. NiO sol-gel precursor solution was prepared by adding anhydrous NiCl_2_ (1 g, Sigma) and tri-block co-polymer F108 [poly (ethylene glycol)-block-poly (propylene glycol)-block-poly (ethylene glycol)] (1g, Sigma) to ethanol (6 ml) and ultrapure water (5 ml). The solution was left to age in a vial for 2 weeks and subsequently centrifuged to remove any large aggregated polymer.

The thin films were then prepared by doctor blading onto cleaned conductive fluorine doped tin oxide glass (TEC™ 15, Pilkington), cut into 2 × 2 cm squares. The area of the NiO was 0.79 cm^2^, punched through Scotch Magic Tape. After doctor blading, the film was allowed to dry for 5 min before annealing in a furnace for 30 min at 450°C (in air), with a ramp time of 30 min. The doctor blading was repeated two more times at which point the “three-layer” mesoporous films have an approximate thickness of 1.5 microns (measured with a Dektak^3^ST surface profiler).

Steady-state UV-vis absorption measurements were performed using an Ocean Optics USB-2000 + spectrophotometer connected to a light source with deuterium and tungsten iodide lamps *via* a fibre optic coupling. The following aqueous buffer solutions were used for photoelectrocatalysis experiments: 0.1 M pH 5 acetate buffer, 0.1 M pH 8 phosphate buffer. 0.1 M pH 9.2 carbonate buffer.

### Synthesis

#### Ru(dceb)_2_Cl_2_

Ru(dceb)_2_Cl_2_ was synthesized using a procedure developed in our lab. 0.42 g (2.05 mmol) RuCl_3_.3 H_2_O was dissolved in 45 ml of ethanol. The mixture was brought to reflux and 1 g (4.1 mmol) 4,4′-dicarboxylic acid-2,2′-bipyridine was slowly added over 30 min. The reaction was refluxed for 24 h, after which the ethanol was removed under reduced pressure. The residue was washed with 10 ml diethyl ether and air dried yielding the product as a dark red solid (1.31 g, 1.7 mmol, 83%). 1H-NMR (DMSO-d_6_): 1.309 (t, 4 H), 1.4495 (t, 4H), 4.36 (q, 3H), 4.5322 (q, 3H), 7.494 (m, 2H), 7.761 (m, 2H), 8.258 (m, 2H), 8.931 (m, 2H), 9.117 (m, 2H), 10.105 (m, 2H).

#### [Ru(dceb)_2_(bpt)](PF_6_) (Ru)

0.42 g 3,5-di(pyridin-2-yl)-4H-1,2,4-triazole (HBpt) (1.88 mmol 1.4 equivalents) was dissolved in 300 ml of a 2:1 ethanol/water mixture. The solution was refluxed for 20 min 1 g (1.30 mmol) of Ru(dceb)_2_Cl_2_ was dissolved in 200 ml ethanol and added to the reaction mixture over 30 min. The reaction was refluxed for 6 h, after which the ethanol was removed under reduced pressure. 200 ml of water was added, the mixture was filtered and 0.4 g (2.45 mmol, 1.9 equivalents) of NH_4_PF_6_ was added. The product precipitated out of solution as a red solid which was further purified by column chromatography using SiO_2_ as stationary phase and 10:1:0.1 acetonitrile/water/10% KNO_3_ solution in water as the eluent yielding **Ru** as a dark red solid (0.33 g, 0.26 mmol, 20%). 600 MHz ^1^H-NMR (DMSO-d_6_): 1.346 ppm (12H, m), 4.426 ppm (8H, m), 7.271 (1H, td), 7.304 (1H, td), 7.538 (1H, d), 7.775 (2H, m),7.860 (1H, dd), 7.917 (2H, d),7.968 (1H, dd), 8.020 (1H, dd), 8.043–8.086 (2H, m), 8.098 (1H, d), 8.119 (1H, d), 8.257 (1H, d), 8.467 (1H, d)9.225 (2H, dd), 9.250 (2H, dd). ESI mass spectroscopy: 924.205 (calc.), 924.2046 (found).

#### [Ru(dceb)_2_(bpt)Re(CO)_3_Cl](PF_6_) (RuRe)

0.1 g (0.082 mmol) of **Ru** was dissolved in 5 ml of methanol. 0.046 g (0.124 mmol, 1.5 equivalents) of Re(CO)_5_Cl was added. The flask was wrapped in tin foil to avoid any contact with light. The reaction was refluxed for 2 hours, after which the solvent was removed under reduced pressure. 5 ml of ether was added and the formed suspension was stirred for an additional 30 min to remove unreacted Re(CO)_5_Cl. The mixture was filtered and air dried to yield **RuRe** as a purple-red solid (0.098 g, 0.07 mmol, 87%). ^1^H-NMR (DMSO-d_6_): 1.361 (12H, m), 4.443 (8H, m), 7.562 (1H, td), 7.62 (1H, d), 7.641 (1H, td), 7.748 (1H, dd), 7.797 (1H, dd), 7.886 (2H, d), 7.995 (1H, dd), 8.037 (1H, d), 8.073–8.152 (m, 4H), 8.346 (1H, td), 8.604 (1H, d), 8.989 (1H, d), 9.248–9.299 (4H, m). ESI mass spectroscopy: 1230.115 (calc.), 1230.113 (found).

#### [Ru(dceb)_2_(bpt)Pt(I)H_2_O)](PF_6_)_2_ (RuPt)

0.1 g **Ru** (0.082 mmol) and 0.075 g (0.124 mmol, 1.5 eq.) Pt(I)_2_(DMSO)_2_ were dissolved in 10 ml acetonitrile. The solution was refluxed overnight. The solvent was removed under reduced pressure and 10 ml diethyl ether was added. The suspension was stirred for 30 min to remove unwanted Pt(I)_2_(DMSO)_2_. The suspension was filtered and air dried to yield **RuPt** as a dark-red solid (0.106 g, 0.07 mmol, 85%). 600 MHz ^1^H-NMR (dmso-d_6_): 1.358 ppm (12H, m), 4.426 (8H, m), 7.386 (1H, t), 7.447 (1H, t), 7.576 (1H, d), 7.784–7.863 (3H, m), 7.931 (1H, t), 7.986 (2H, m), 8.021 (1H, d), 8.162 (1H, t), 8.238 (1H, d), 8.356 (2 H, s, br), 8.623 (1H, br), 8.722 (1H, br), 9.128 (1H, s)9,221 (3H, m) ESI-mass spectroscopy: 1372.980 (calc.), found 1372.977(found).

## Data Availability

The original contributions presented in the study are included in the article/Supplementary Material, further inquiries can be directed to the corresponding authors.
