# Supplementary material for: Ruthenium Assemblies for CO2 Reduction and H2 Generation: Time Resolved Infrared Spectroscopy, Spectroelectrochemistry and a Photocatalysis Study in Solution and on NiO
Source: Front Chem. 2021 Dec 24;9:795877. doi: 10.3389/fchem.2021.795877 (PMC8738169; doi:10.3389/fchem.2021.795877)
Supplement: Supplementary file 1 [file DataSheet1.PDF]

Table of Contents

Electrochemistry 2

FTIR spectroelectrochemistry 8

[Ru(dceb)$_2$(bpt)](PF$_6$) (**Ru**) 8

[Ru(dceb)$_2$(bpt)Re(CO)$_3$Cl](PF$_6$)$_2$ (**RuRe**) 10

[Ru(dceb)$_2$(bpt)PtI(H$_2$O)](PF$_6$)$_2$ (**RuPt**) 11

UV-Vis spectroelectrochemistry 13

[Ru(dceb)$_2$(bpt)](PF$_6$) (**Ru**) 13

[Ru(dceb)$_2$(bpt)PtI$_2$](PF$_6$) (**RuPt**) 15

[Ru(dceb)$_2$(bpt)Re(CO)$_3$Cl](PF$_6$)$_2$ (**RuRe**) 17

Time resolved infrared spectroscopy 19

[Ru(dceb)$_2$(bpt)](PF$_6$)$_2$ (**Ru**) 19

[Ru(dceb)$_2$(bpt)Re(CO)$_3$Cl](PF$_6$) (**RuRe**) 20

Time-Resolved emission spectroscopy 23

Photocatalysis 26

Surface studies 28

NMR spectra 43

Mass spectrometry 54

Electrochemistry

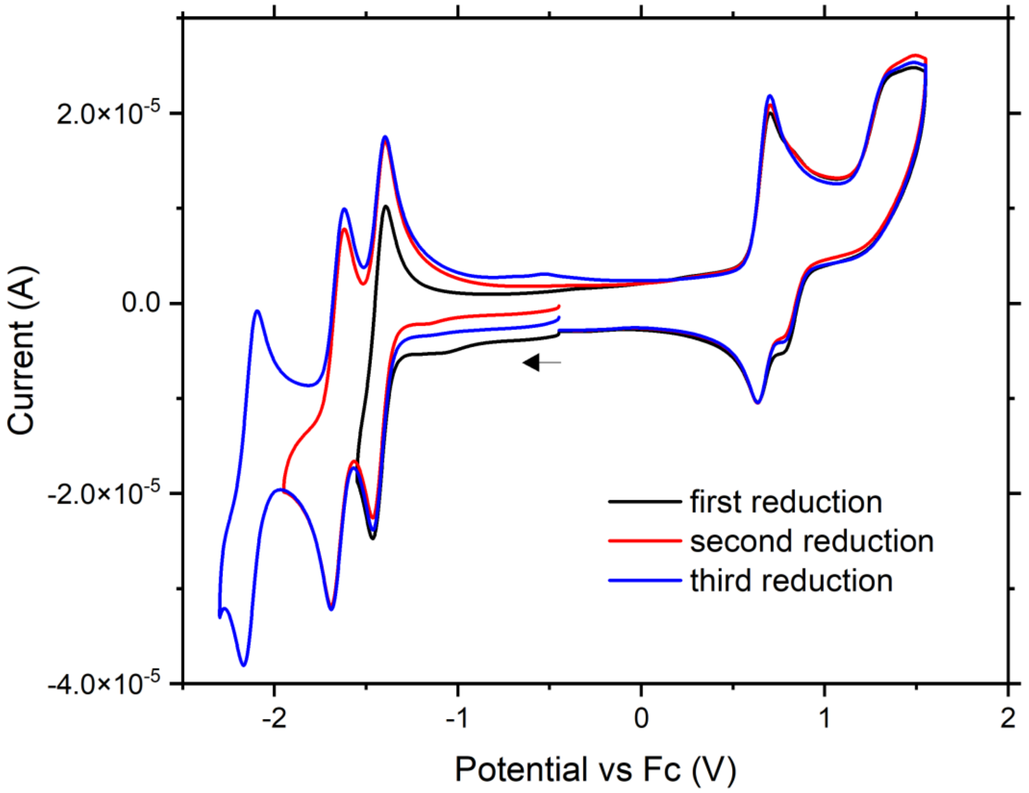

Figure S1. Cyclic voltammogram of **Ru**. Experiments were run with 1 mM **Ru** in nitrogen purged acetonitrile with 0.1 M tetrabutylammonium hexafluorophosphate as supporting electrolyte. The reversibility of the three reductions was probed by returning the scan at various potentials. A glassy carbon electrode, platinum wire and Ag/$AgNO_3$ electrode were used as working, counter and reference electrode, respectively. Ferrocene was added as internal reference. Scans were taken at 0.1 V/s.

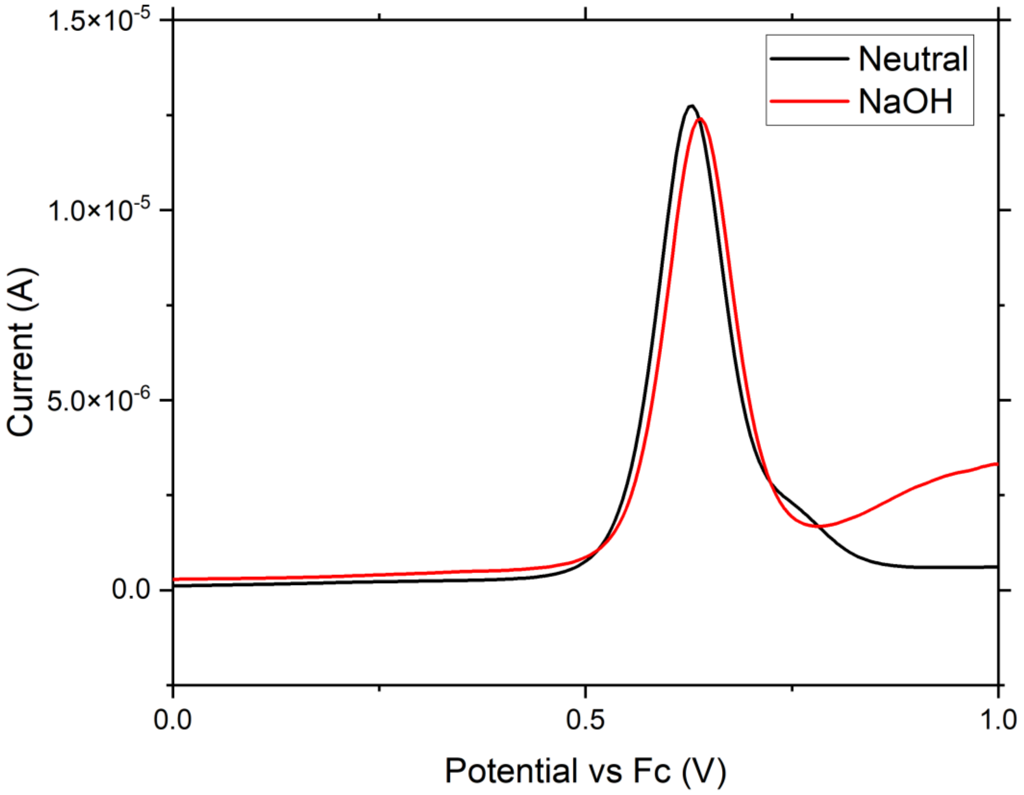

Figure S2. DPV study of the oxidation for **Ru** in the absence (black) and presence (red) of 0.02 M NaOH. Experiments were run with 1 mM **Ru** in nitrogen purged acetonitrile with 0.1 M tetrabutylammonium hexafluorophosphate as supporting electrolyte. A glassy carbon electrode, platinum wire and Ag/$AgNO_3$ electrode were used as working, counter and reference electrode, respectively. Ferrocene was added as internal reference. Scans were taken with an increment of 0.005 V, amplitude of 0.025 V, pulse width of 0.05 s, sample width of 0.02 s and pulse period of 1 s.

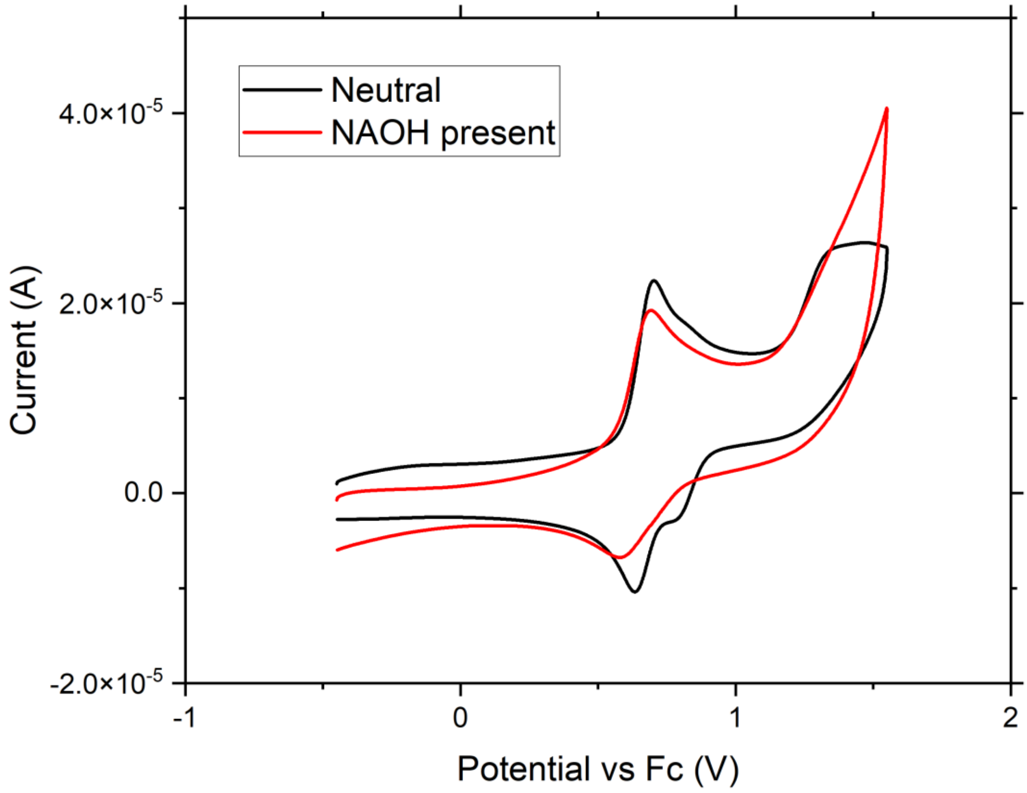

Figure S3. CV study of the oxidation for **Ru** in the absence (black) and presence (red) of 0.02 M NaOH. Experiments were run with 1 mM **Ru** in nitrogen purged acetonitrile with 0.1 M tetrabutylammonium hexafluorophosphate as supporting electrolyte. A glassy carbon electrode, platinum wire and Ag/$AgNO_3$ electrode were used as working, counter and reference electrode, respectively. Ferrocene was added as internal reference. Scans were taken at 0.1 V/s.

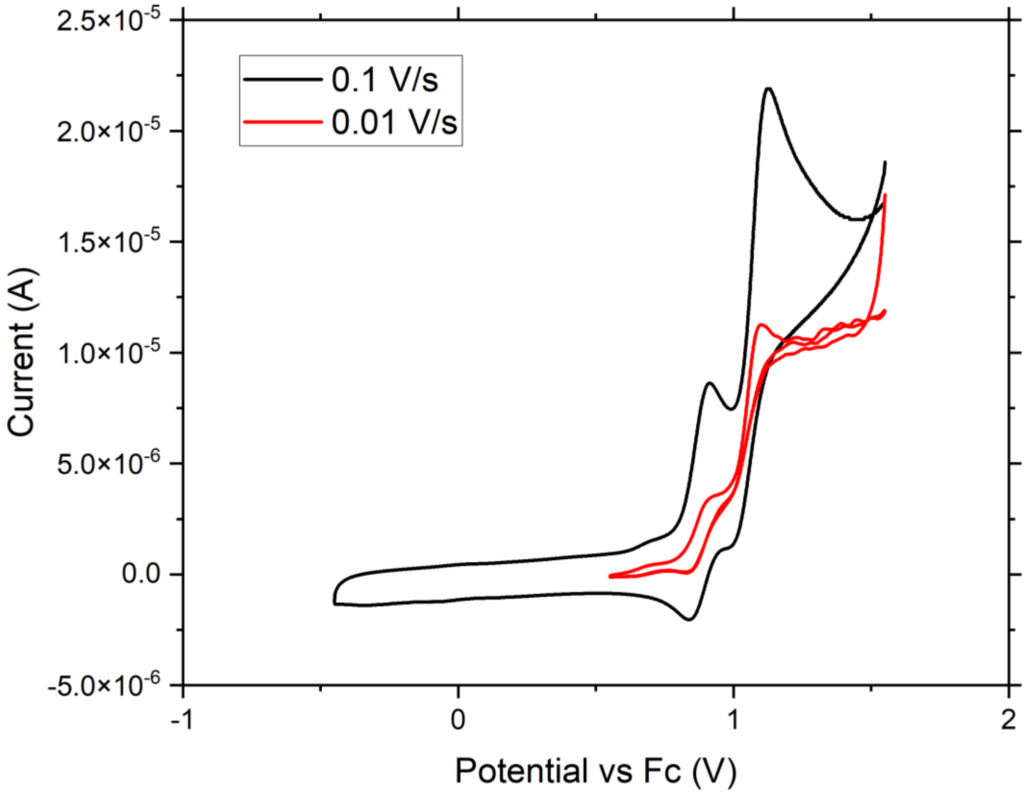

Figure S4. Scan rate study of the oxidations for **RuRe**. Cyclic voltammograms between -0.45 V and 1.55 V at 0.1 V/s (black) and 0.01 V/s (red). Experiments were run with 1 mM **RuRe** in nitrogen purged acetonitrile with 0.1 M tetrabutylammonium hexafluorophosphate as supporting electrolyte. A glassy carbon electrode, platinum wire and $Ag/AgNO_3$ electrode were used as working, counter and reference electrode, respectively. Ferrocene was added as internal reference.

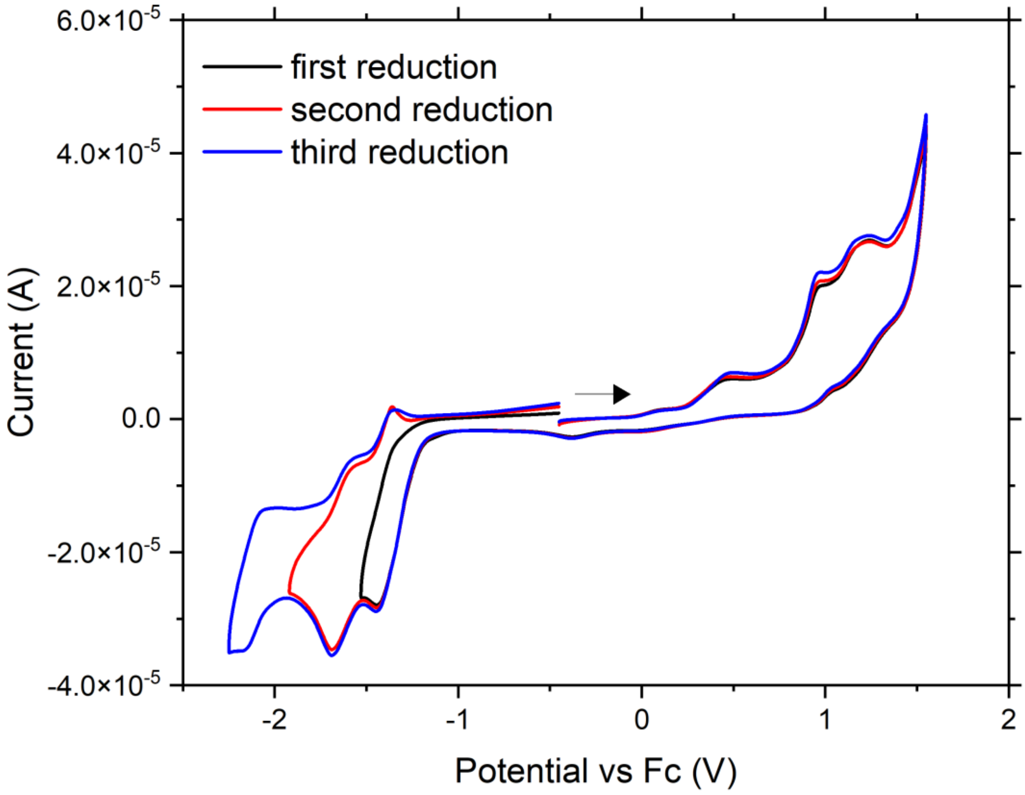

Figure S5. Cyclic voltammogram of **RuPt**. Experiments were run with 1 mM **RuPt** in nitrogen purged acetonitrile with 0.1 M tetrabutylammonium hexafluorophosphate as supporting electrolyte. The reversibility of the three reductions was probed by returning the scan at various potentials. A glassy carbon electrode, platinum wire and Ag/$AgNO_3$ electrode were used as working, counter and reference electrode, respectively. Ferrocene was added as internal reference. Scans were taken at 0.1 V/s.

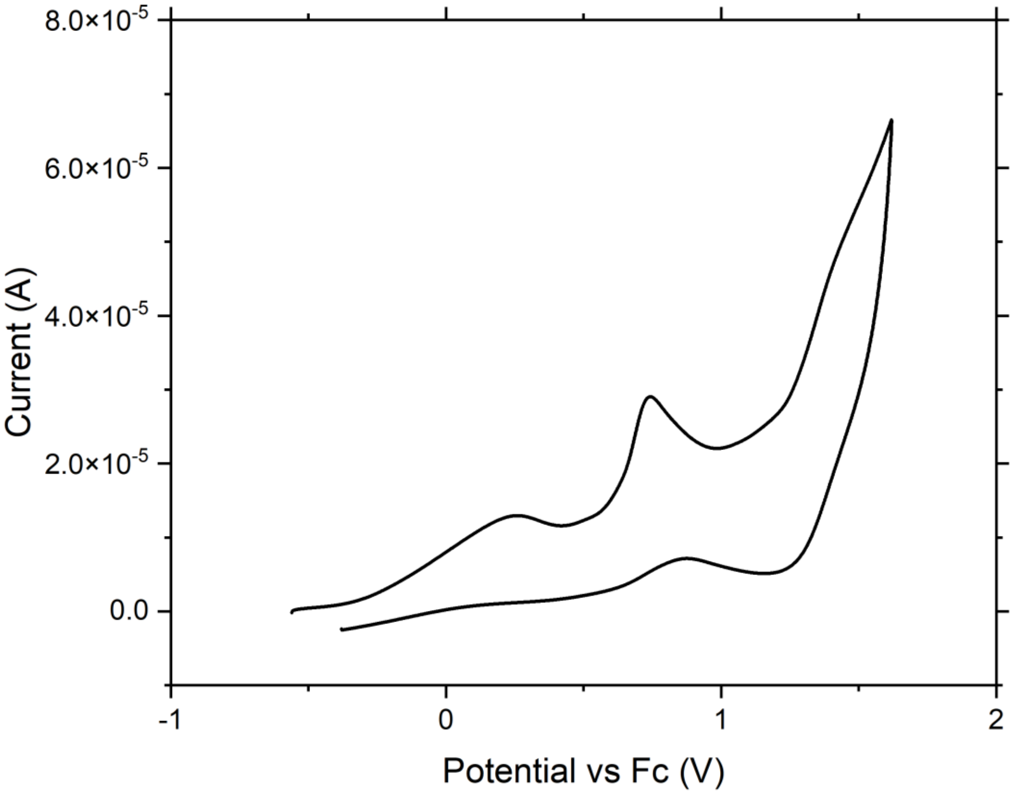

Figure S6. Cyclic voltammogram of the Nabpt ligand. Experiment was conducted with 1 mM of the Hbpt ligand in dry nitrogen purged acetonitrile with 0.1 M tetrabutylammonium hexafluorophosphate as supporting electrolyte. 0.1 M NaOH was added to deprotonate the ligand and form the Nabpt complex to enable the ligand to dissolve in the solution.

FTIR spectroelectrochemistry

[Ru(dceb)$_2$(bpt)](PF$_6$) (**Ru**)

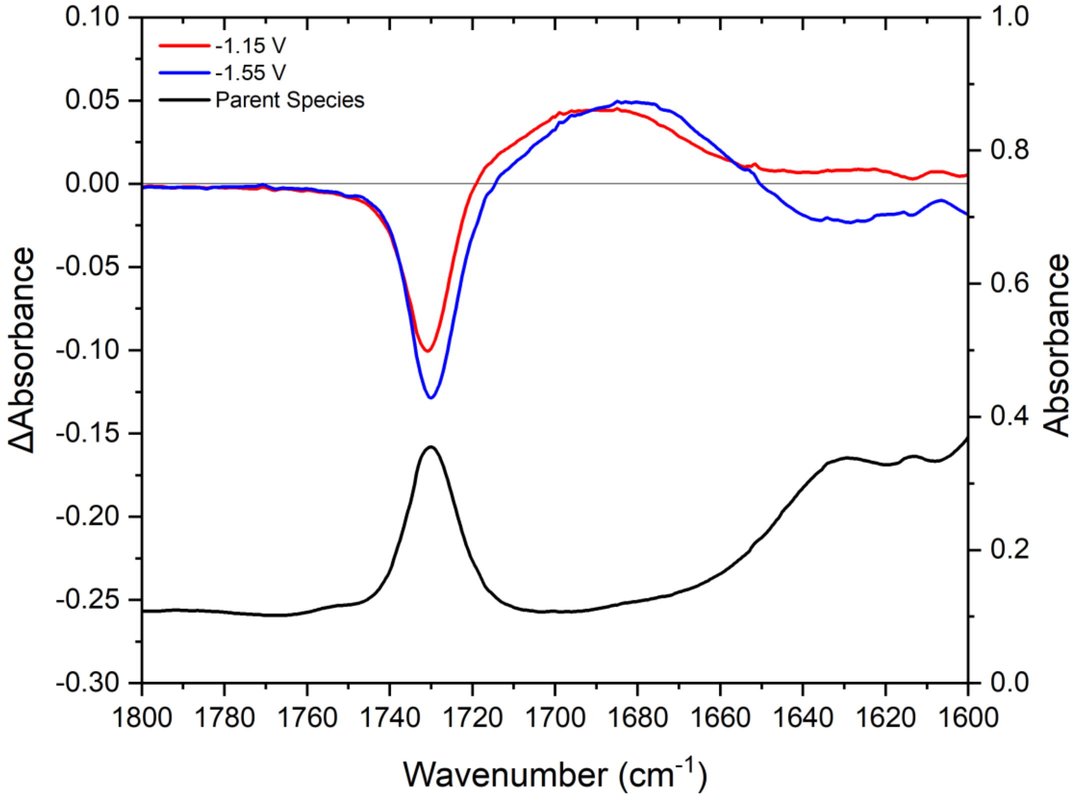

Figure S7. 2.5 mM **Ru** in 0.1 M TBPAF$_6$/ACN. The IR spectrum (black) and difference spectra at -1.15 (red) and -1.55 V (blue) are shown. All potentials vs the silver wire pseudo-reference. The signal at 1632 cm^{-1} is a residual signal from solvent.

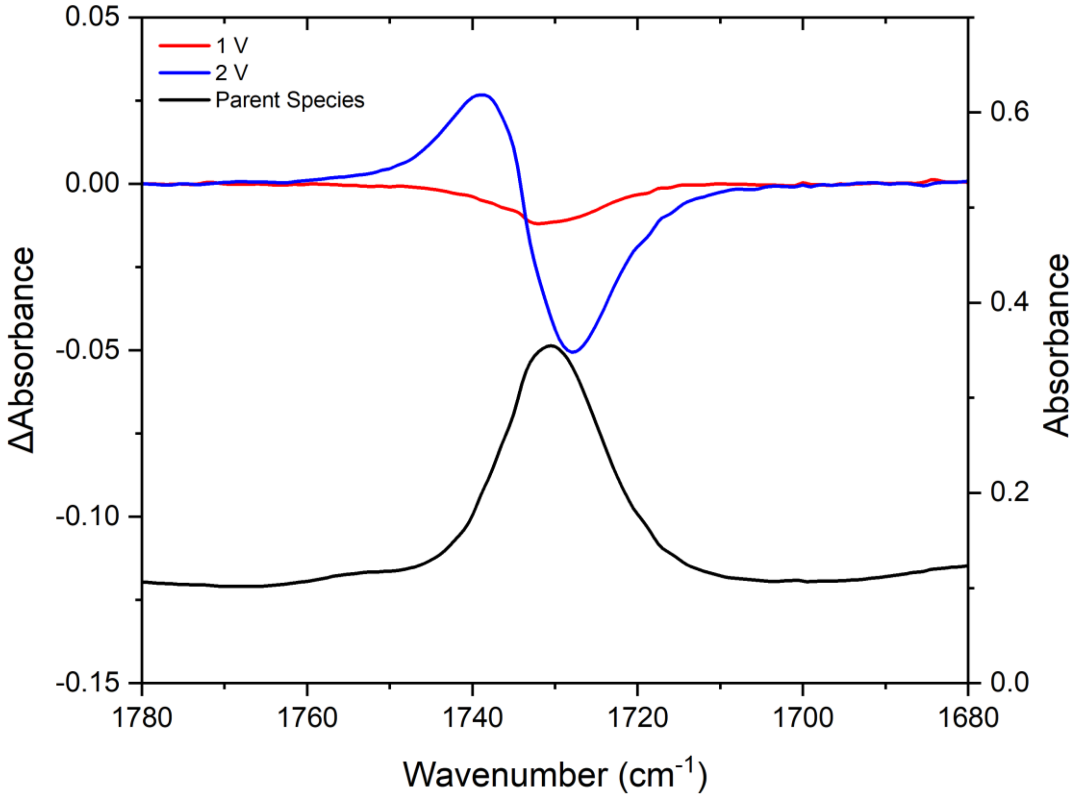

Figure S8. 2.5 mM **Ru** in 0.1 M $TBPAF_6$/ACN. IR spectrum (black) and difference spectra at 1 V (red) and 2 V (blue) vs silver wire pseudo-reference.

[Ru(dceb)$_2$(bpt)Re(CO)$_3$Cl](PF$_6$)$_2$ (**RuRe**)

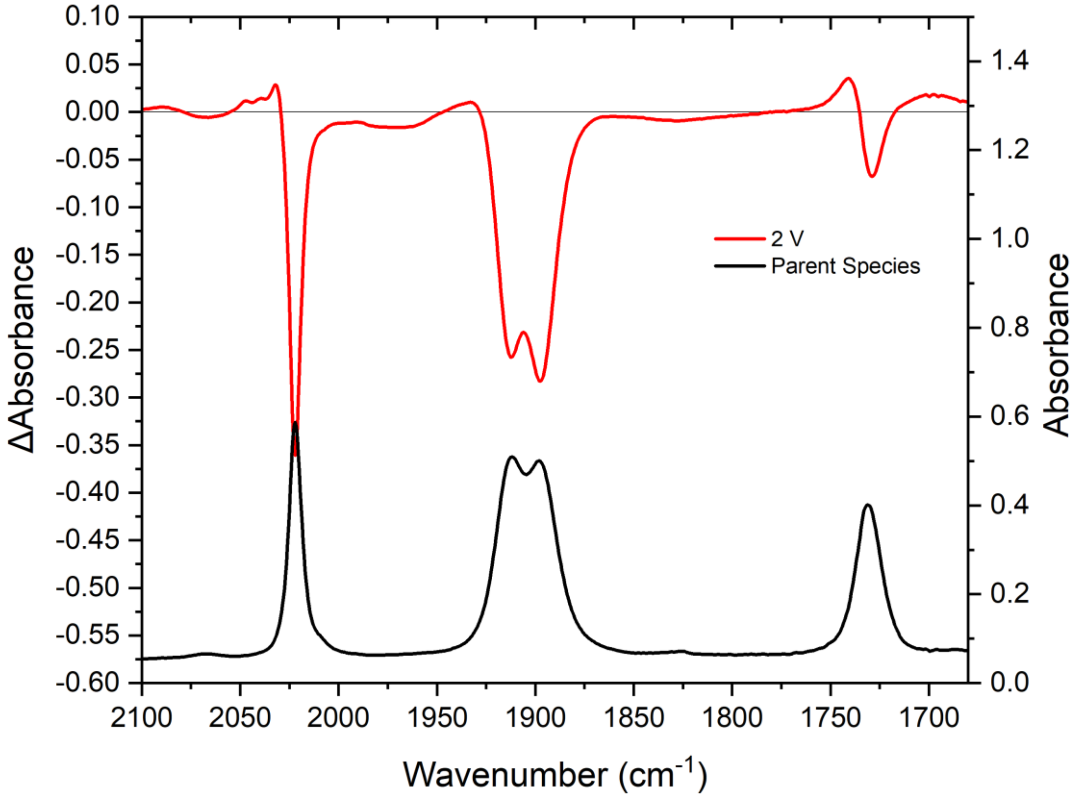

Figure S9. 2.5 mM **RuRe** in 0.1 M TBPAF$_6$/ACN. The IR spectrum (black) and difference spectra at 2 V (red) vs silver wire pseudo-reference.

[Ru(dceb)$_2$(bpt)PtI(H$_2$O)](PF$_6$)$_2$ (**RuPt**)

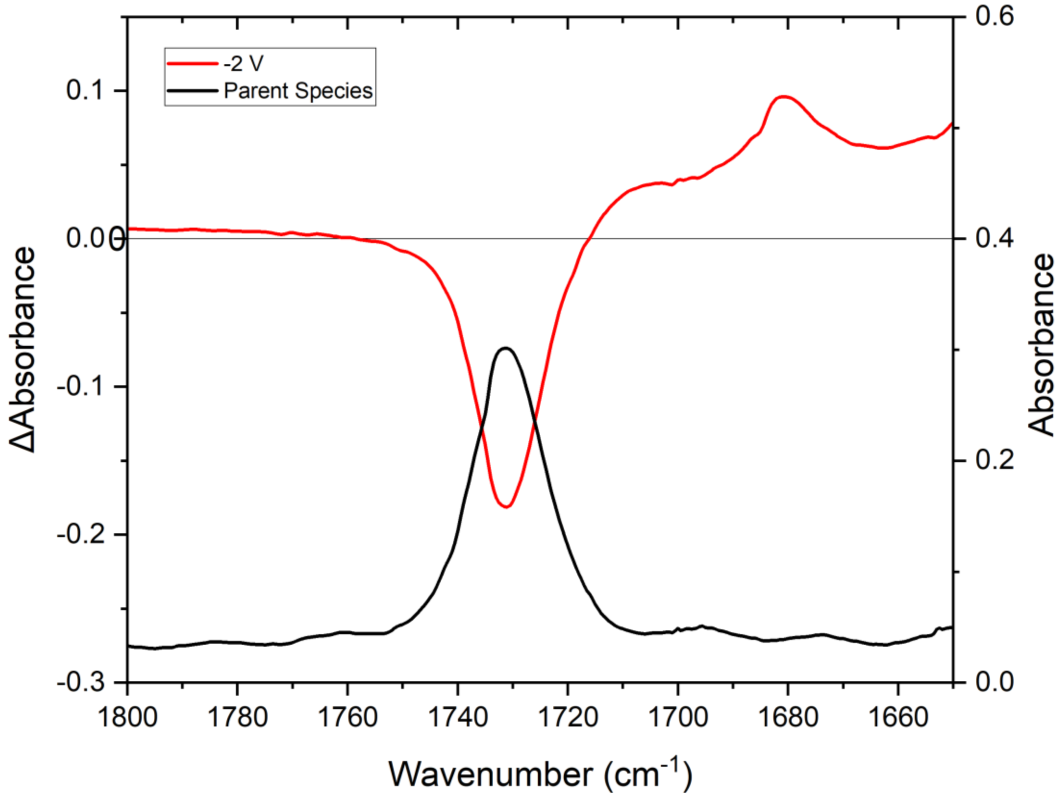

Figure S10. 2.5 mM **RuPt** in 0.1 M TBPAF$_6$/ACN. The IR spectrum (black) and difference spectra at -2 V (red) vs silver wire pseudo-reference.

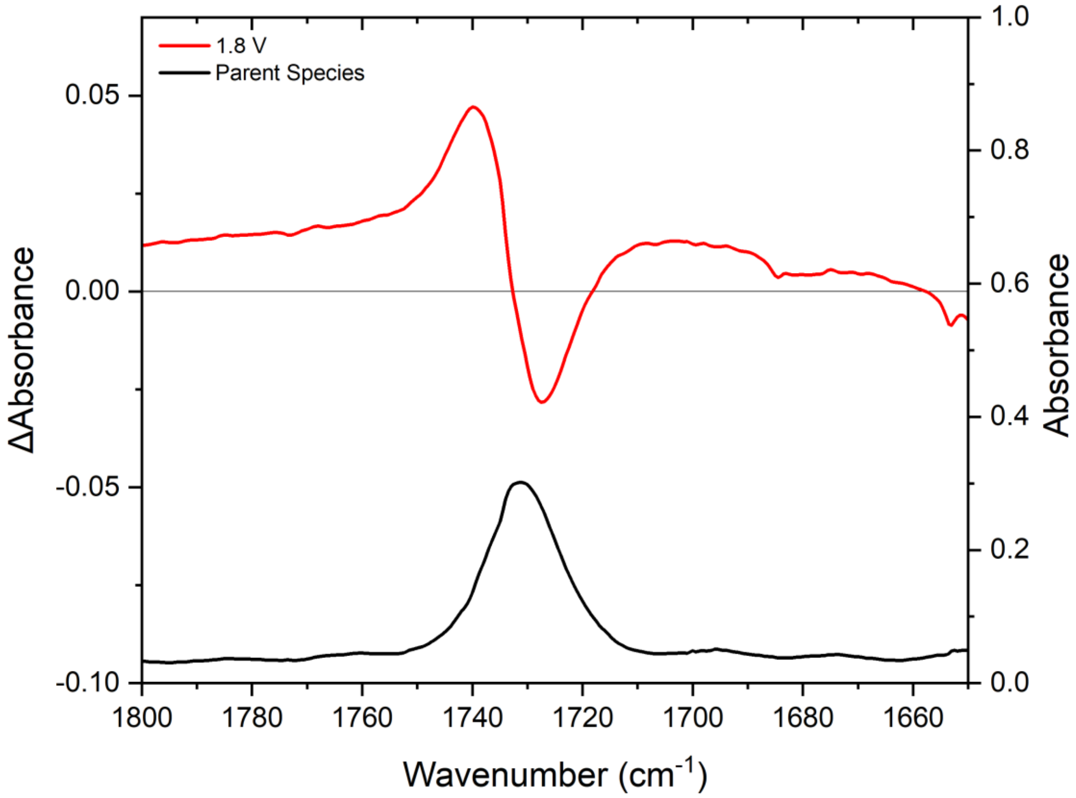

Figure S11. 2.5 mM **RuPt** in 0.1 M TBPAF6/ACN. The IR spectrum (black) and difference spectra at 1.8 V (red) vs silver wire pseudo-reference.

UV-Vis spectroelectrochemistry

$[Ru(dceb)_2(bpt)](PF_6)$ (**Ru**)

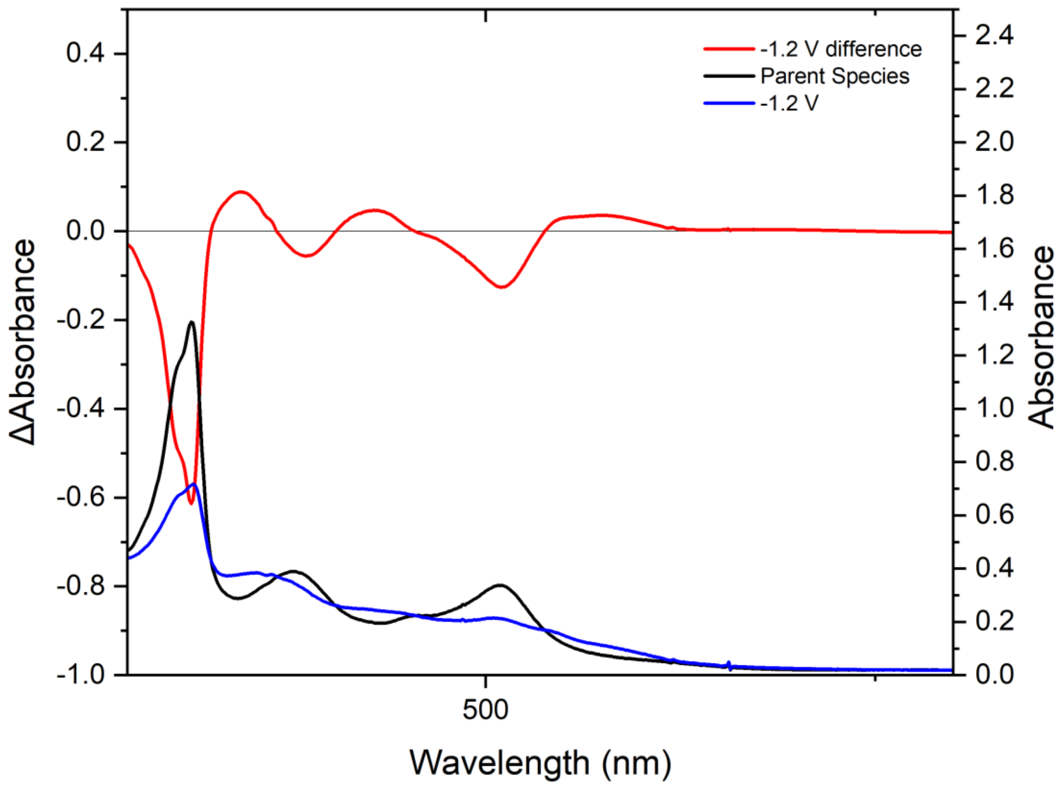

Figure S12. Ru reductive UV-Vis spectroelectrochemistry. The UV-Vis spectrum at open circuit potential (black), and at -1.2 V (blue) as well as the difference spectrum at -1.2 V (red) vs silver wire pseudo-reference.

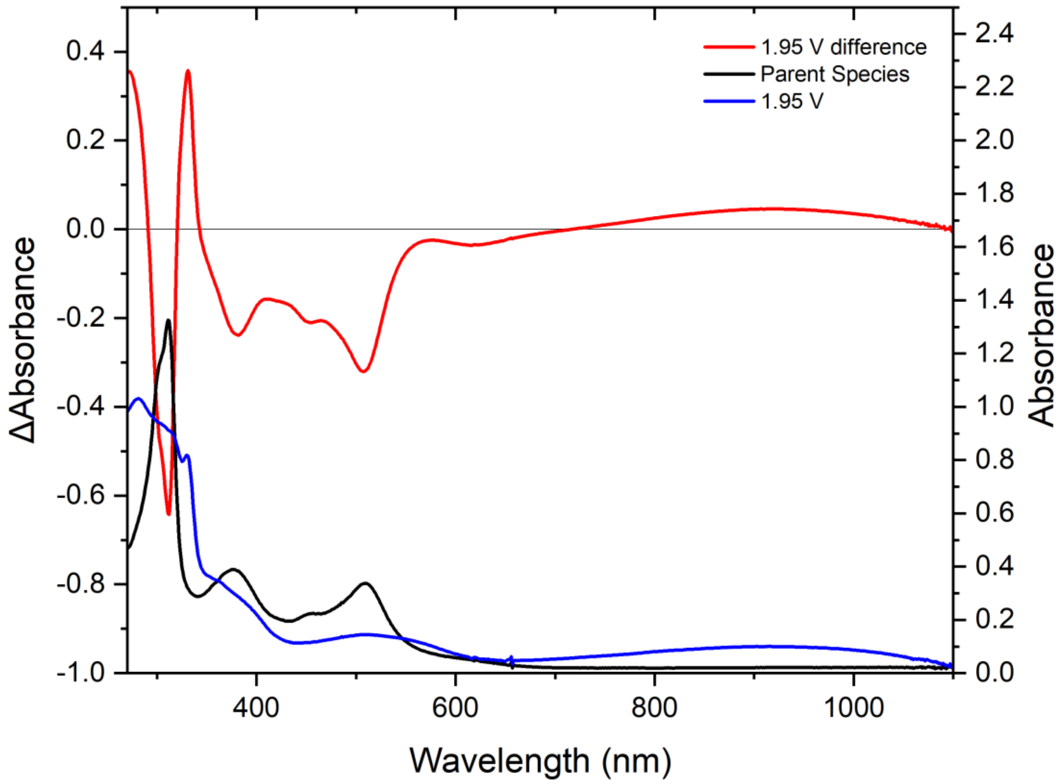

Figure S13. Ru oxidative UV-Vis spectroelectrochemistry. The UV-Vis spectrum at open circuit potential (black) and at 1.95 V (blue) as well as the difference spectrum at 1.95 V (red) vs silver wire pseudo-reference.

[Ru(dceb)$_2$(bpt)PtI$_2$](PF$_6$) (**RuPt**)

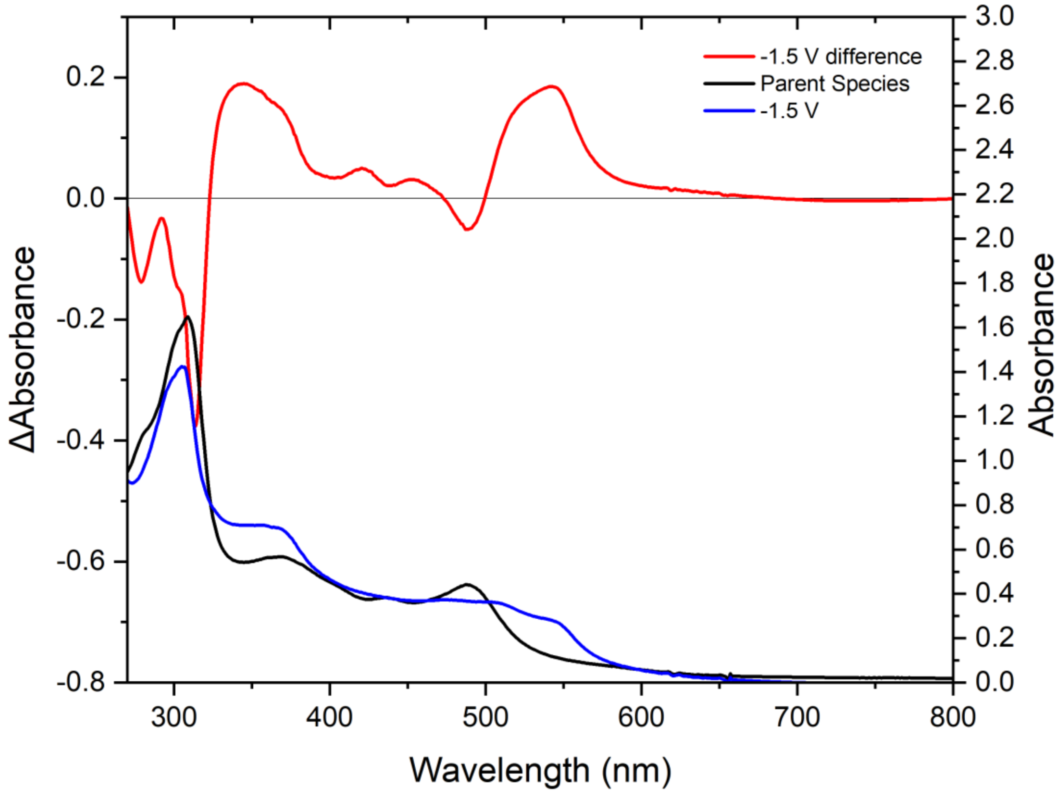

Figure S14. **RuPt** reductive UV-Vis spectroelectrochemistry. The UV-Vis spectrum at open circuit potential (black) and at -1.5 V (blue), as well as the difference spectrum at -1.5 V (red) vs silver wire pseudo-reference.

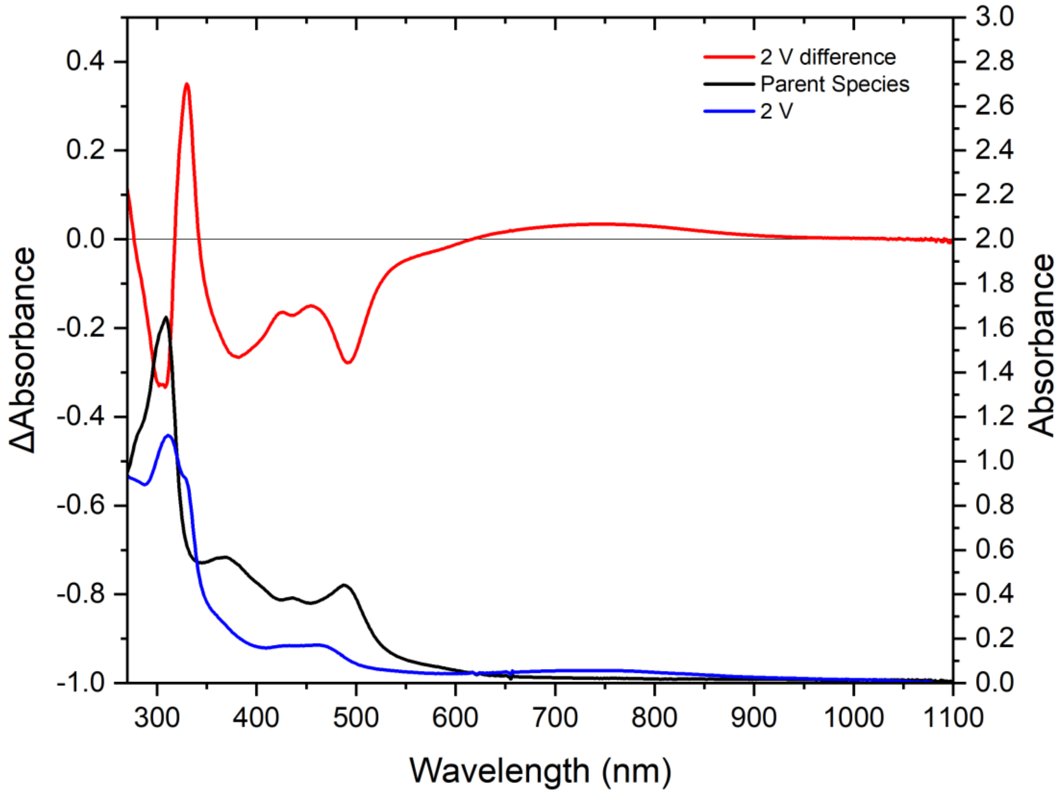

Figure S15. **RuPt** oxidative spectroelectrochemistry. The UV-Vis spectrum at open circuit potential (black) and at 2 V (blue), as well as the difference spectrum at 2 V (red) vs silver wire pseudo-reference.

[Ru(dceb)$_2$(bpt)Re(CO)$_3$Cl](PF$_6$)$_2$ (**RuRe**)

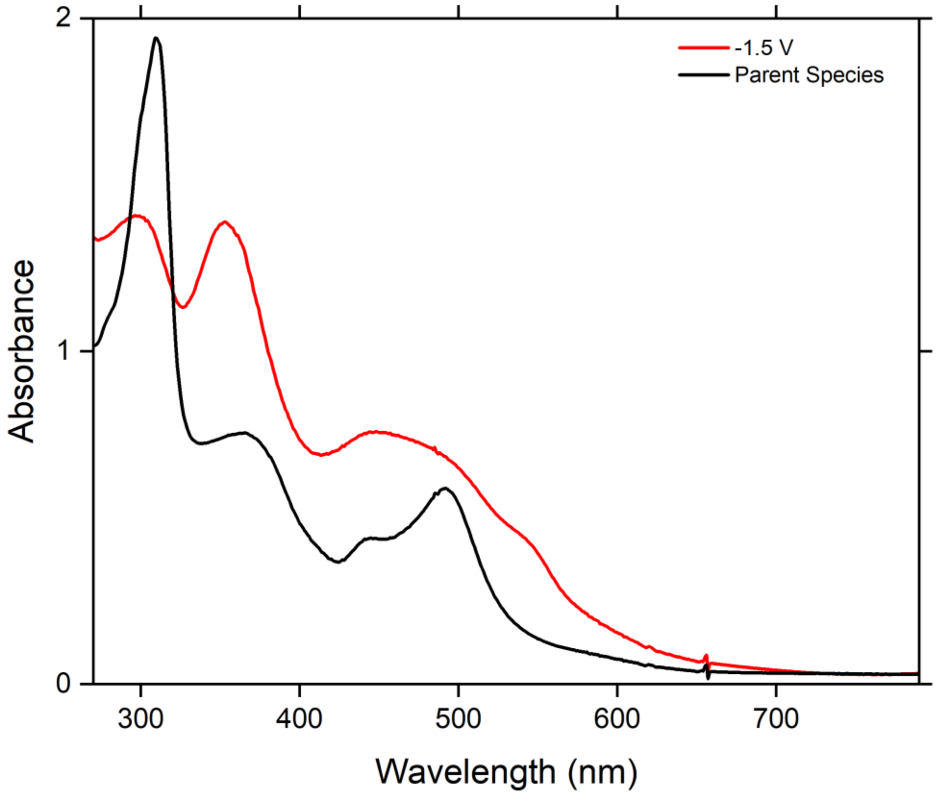

Figure S16. RuRe reductive UV-Vis spectroelectrochemistry. The UV-Vis spectrum at open circuit potential (black) and at -1.5 V (red) vs silver wire pseudo-reference.

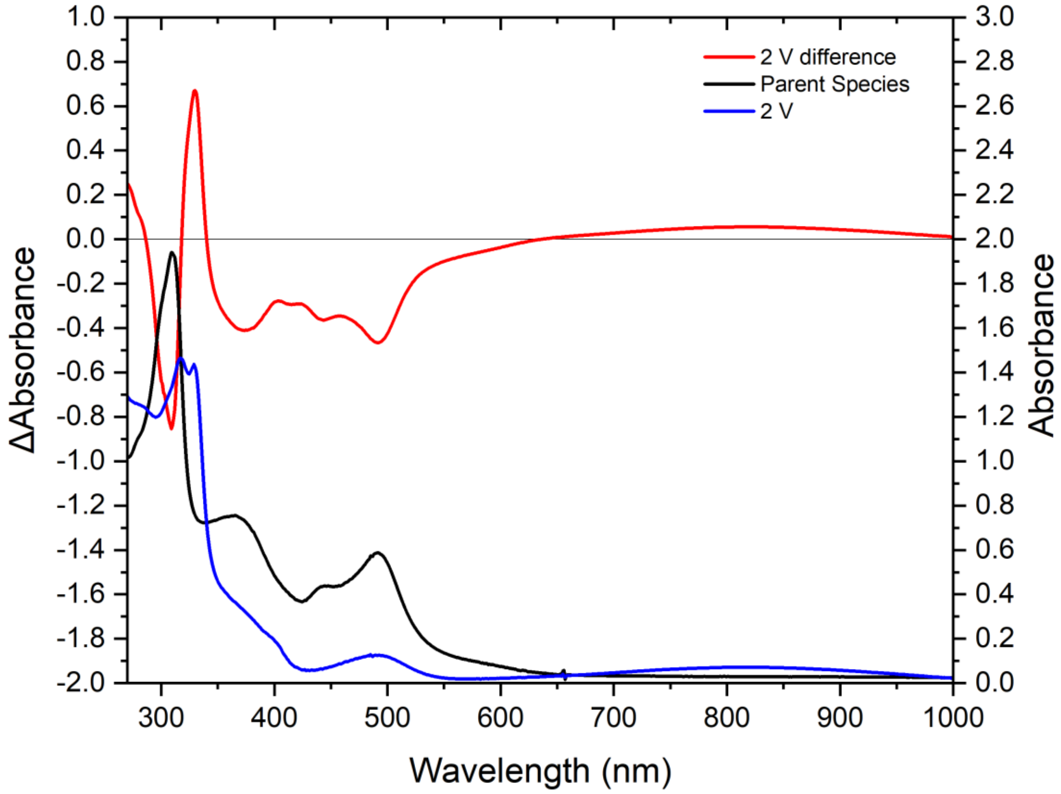

Figure S17. RuRe oxidative UV-Vis spectroelectrochemistry. The UV-Vis spectrum at open circuit potential (black) and at 2 V (blue), as well as the difference spectrum at 2 V (red) vs silver wire pseudo-reference.

Time resolved infrared spectroscopy

[Ru(dceb)$_2$(bpt)](PF$_6$)$_2$ (**Ru**)

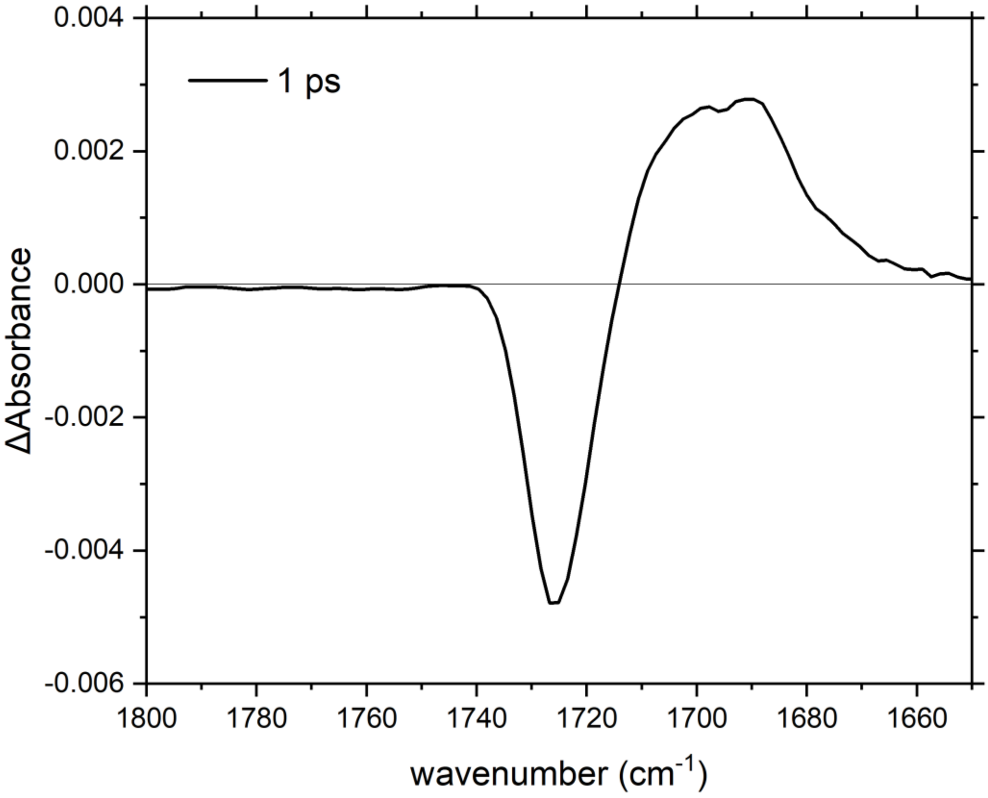

Figure S18. TRIR spectrum of **Ru** in d3-acetonitrile at 1 picosecond. Excited at 510 nm with 1 microjoule energy.

[Ru(dceb)$_2$(bpt)Re(CO)$_3$Cl](PF$_6$) (**RuRe**)

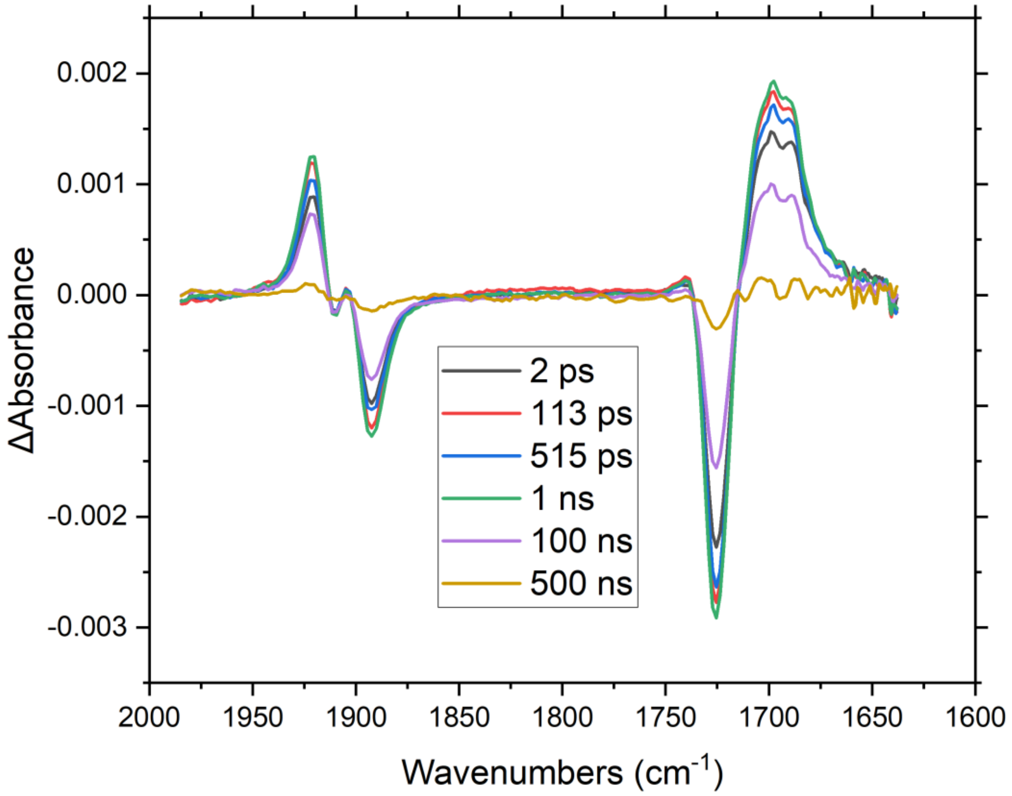

Figure S19. TRIR spectrum of **RuRe** in d$_3$-acetonitrile. Excited at 510 nm with 1 microjoule power. Spectra at 2 ps (black), 113 ps (red), 515 ps (blue), 1 ns (green), 100 ns (purple) and 500 ns (yellow). 1650-2000 cm^{-1} range.

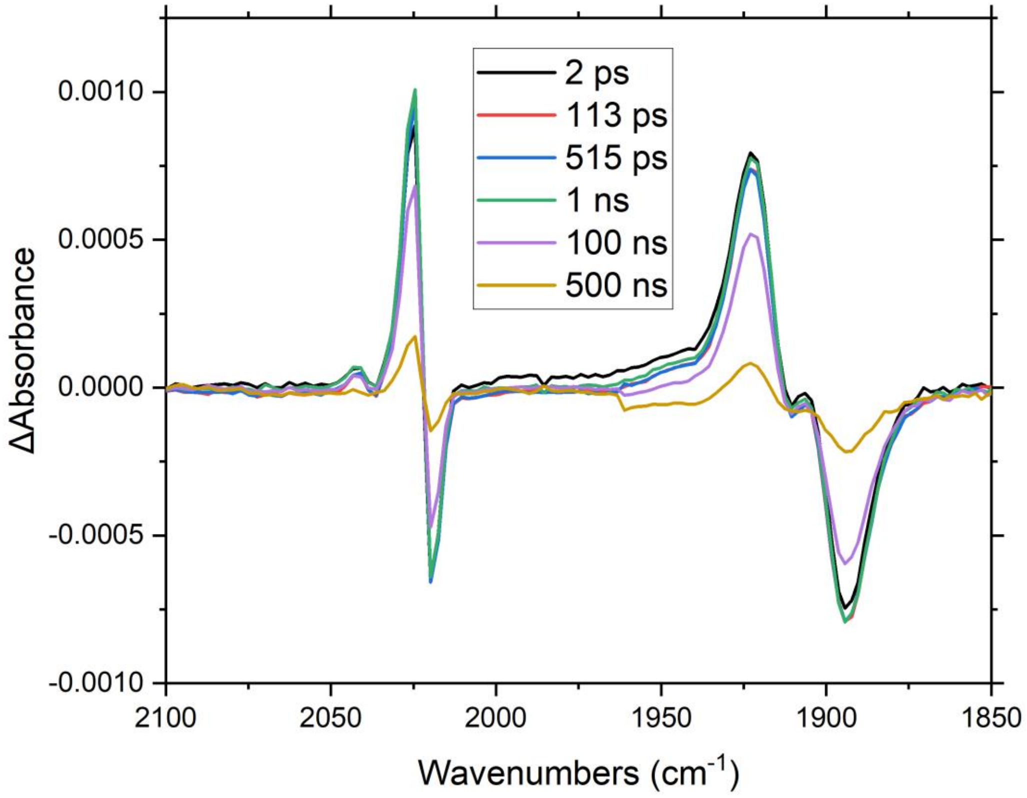

Figure S20. RuRe in d_3-acetonitrile, excited at 510 nm with 1 microjoule power. Spectra at 2 ps (black), 113 ps (red), 515 ps (blue), 1 ns (green), 100 ns (purple) and 500 ns (yellow).. 1750-2150 cm^{-1} range.

Ru(dceb)$_2$(bpt)Pt(I)$_2$ (**RuPt**)

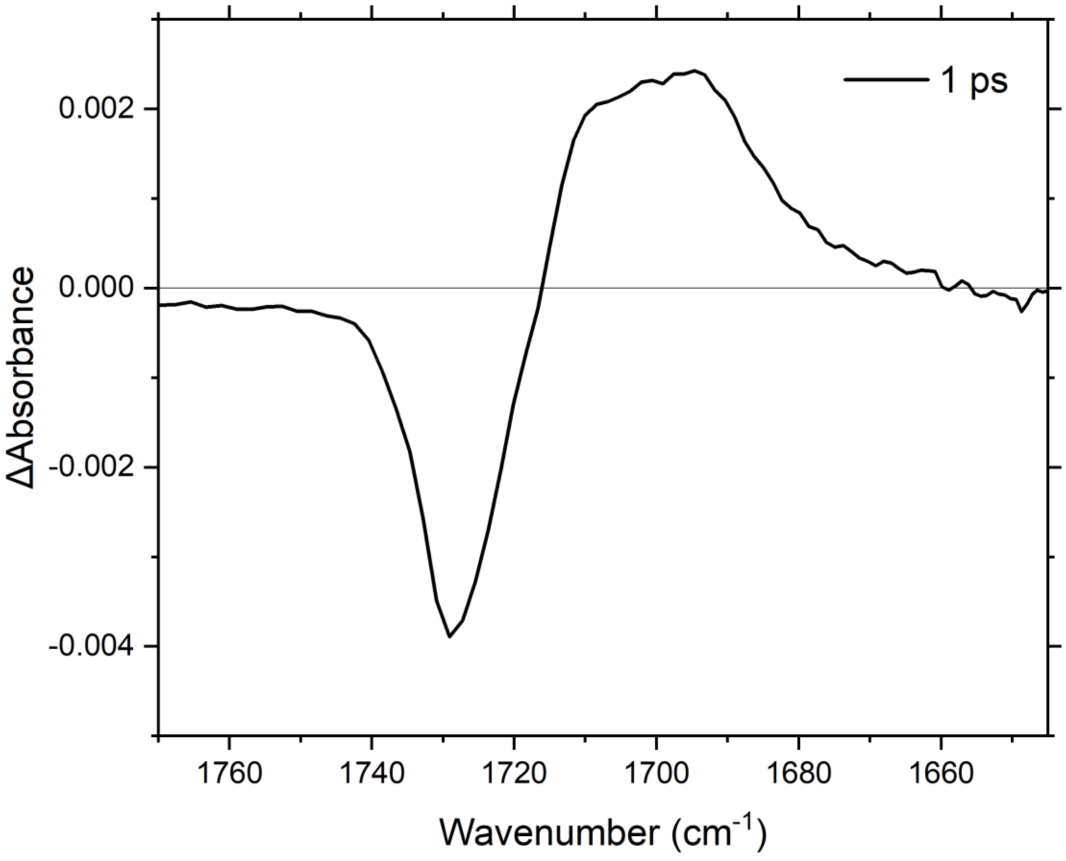

Figure S21 TRIR spectrum of **RuPt** in ACN-d$_3$ at 510 nm excitation with 1 microjoule power. Spectrum at 1 ps.

Time-Resolved emission spectroscopy

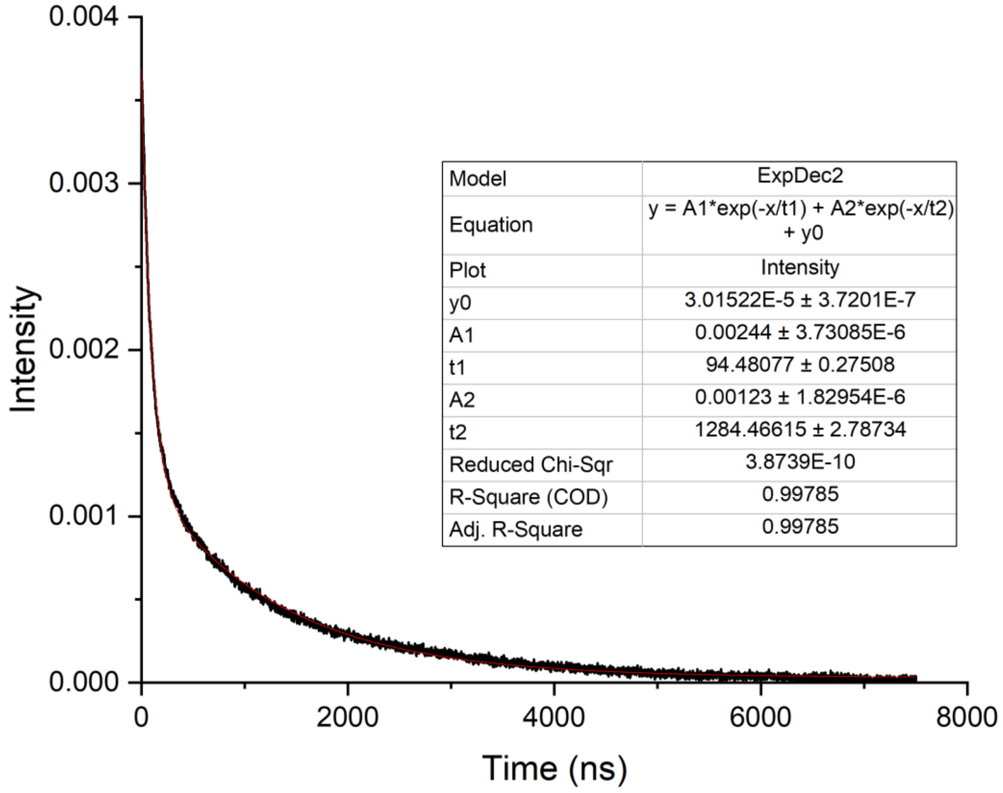

Figure S22. 690 nm emission lifetime of **Ru**. Experiments were run in deaerated acetonitrile at 0.01 mM concentration with 355 nm excitation.

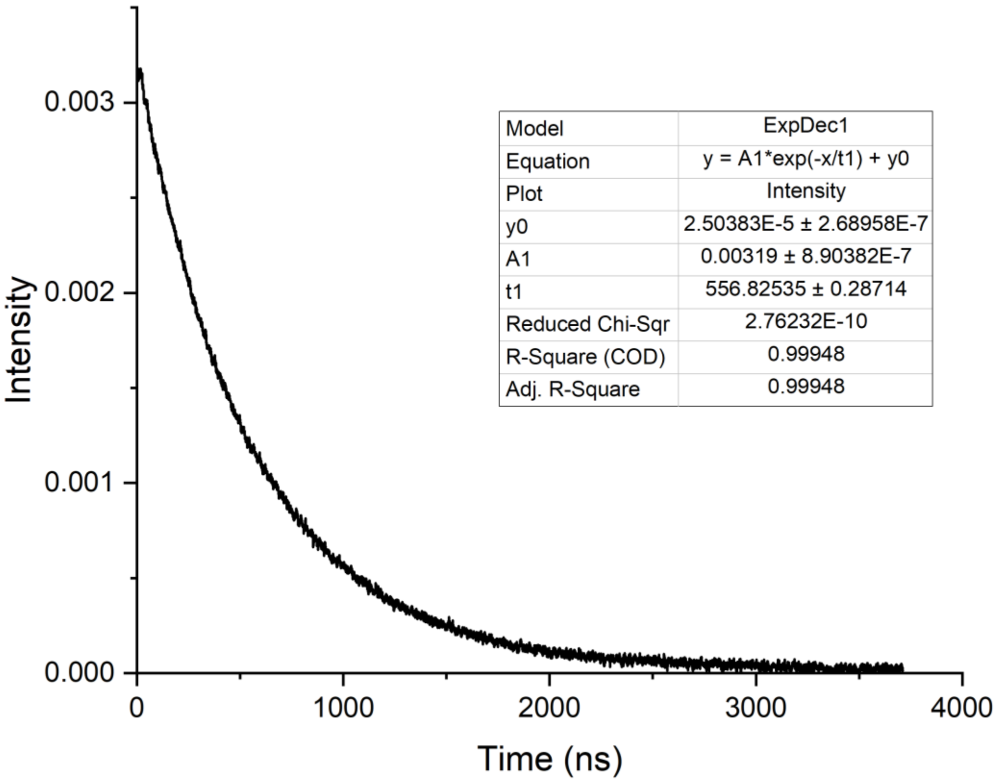

Figure S23. 690 nm emission lifetime of **RuRe**. Experiments were run in deaerated acetonitrile at 0.01 mM concentration with 355 nm excitation.

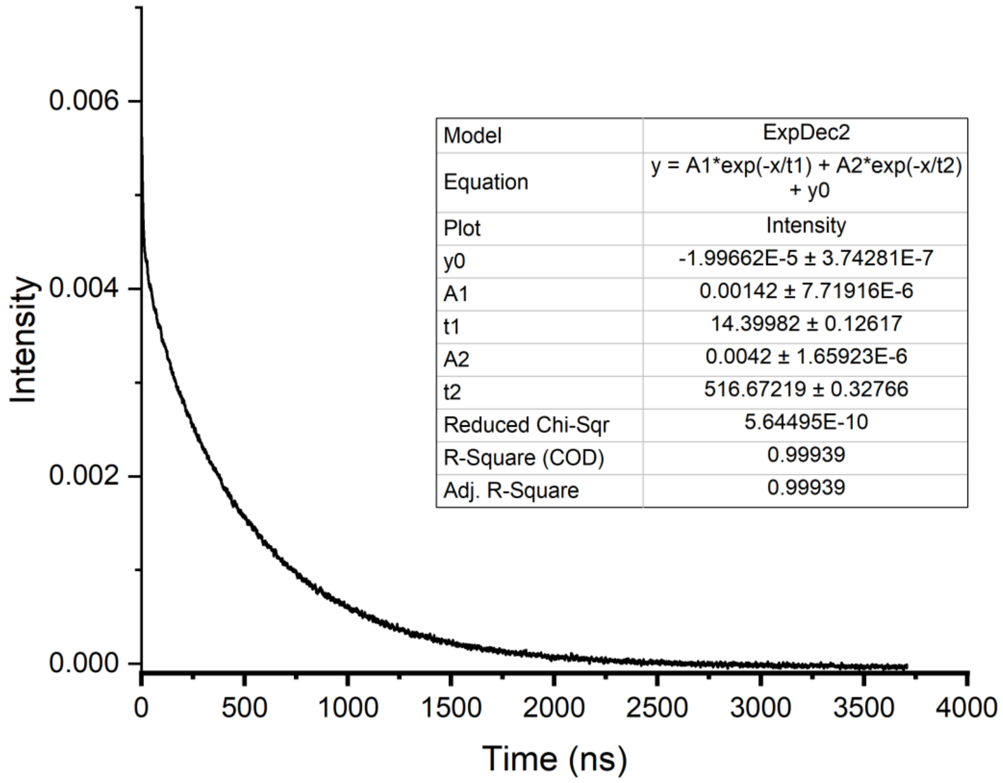

Figure S24. 690 nm emission lifetime of **RuPt**. Experiments were run in deaerated acetonitrile at 0.01 mM concentration with 355 nm excitation.

Photocatalysis

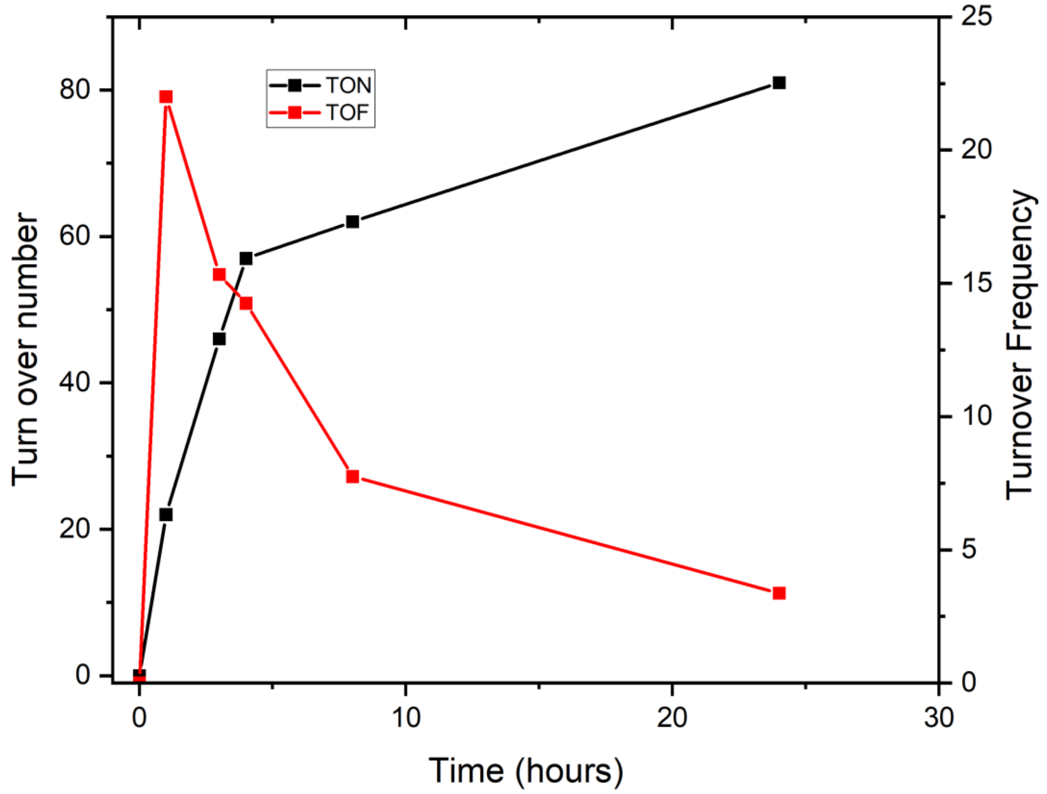

Figure S25. Time dependent photocatalysis for **RuPt**. The turnover numbers at various time internals (black) and TOF (red).

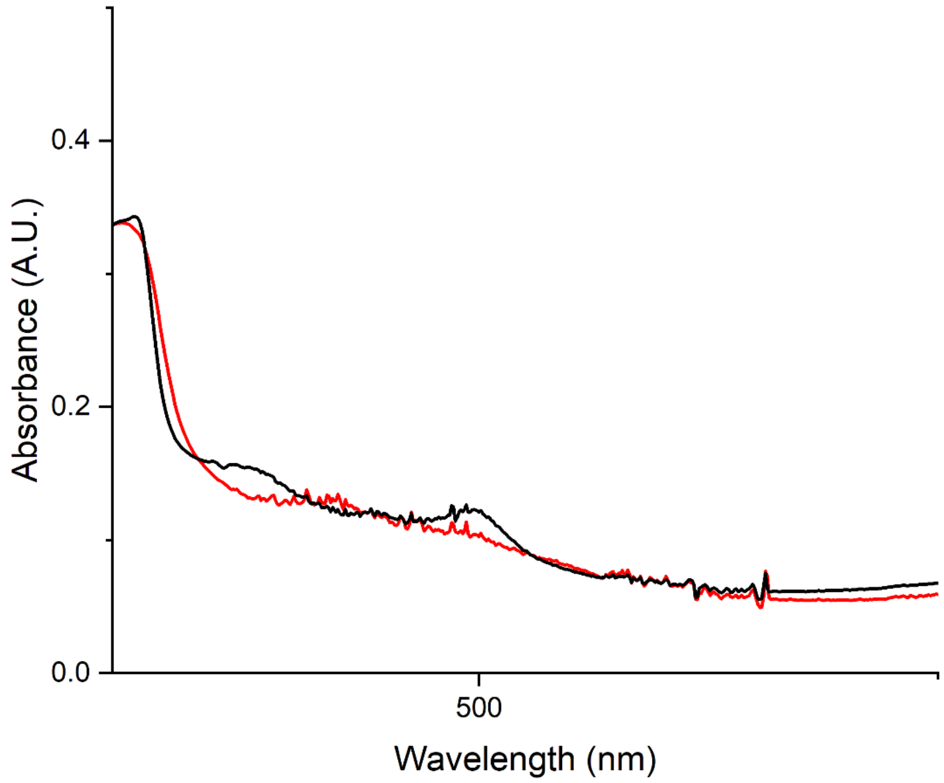

Figure S26 UV-Vis spectrum of **RuPt** in 60:30:10 acetonitrile:triethylamine:water before (black) and after (red) 24 hour of 470 nm irradiation.

Surface studies

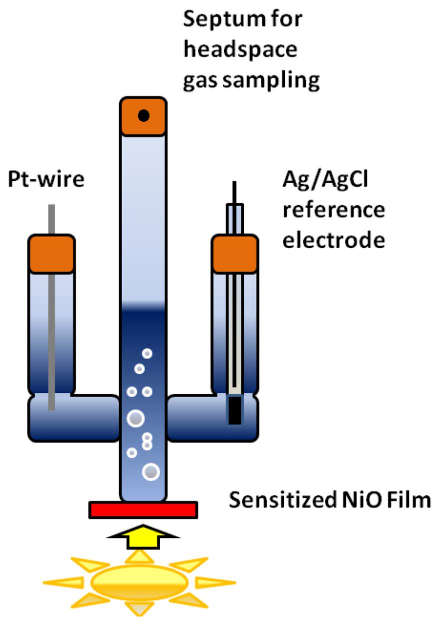

Figure S27. Schematic of photoelectrochemical cell used for CO_2 reduction experiments.

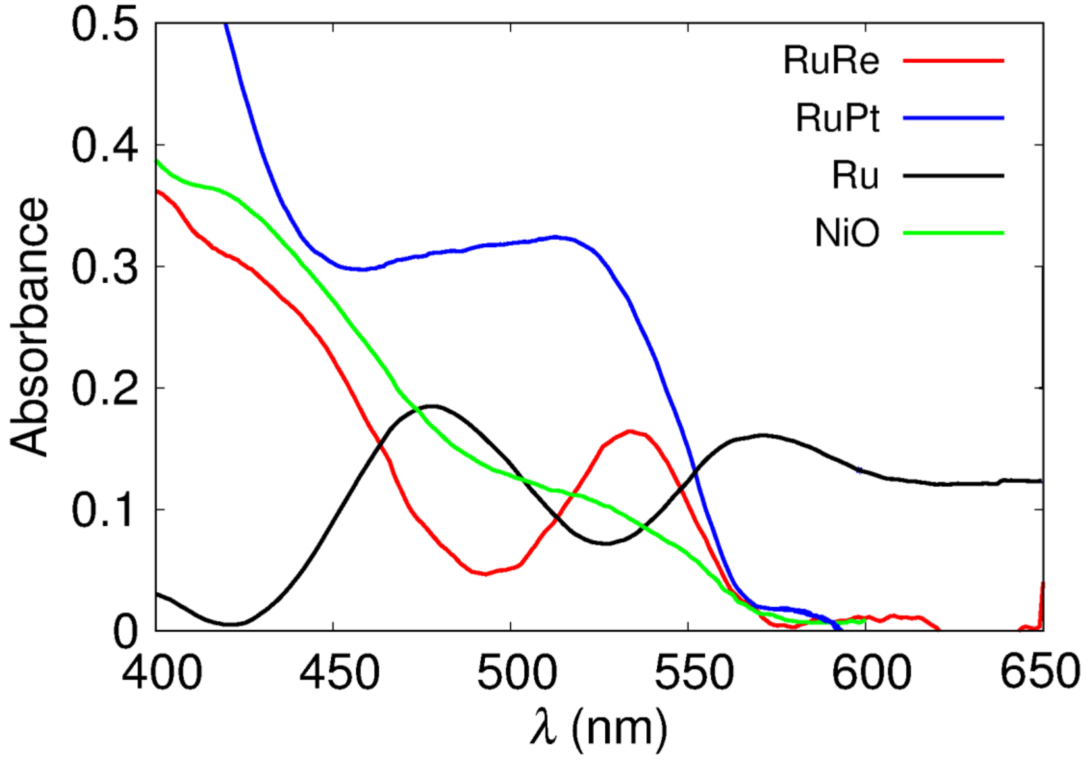

Figure S28. UV-visible absorption spectra of chromophores used in this study on mesoporous NiO thin films. Note the spectrum for NiO has been subtracted from **Ru**, **RuPt** and **RuRe** for clarity and have been normalized.

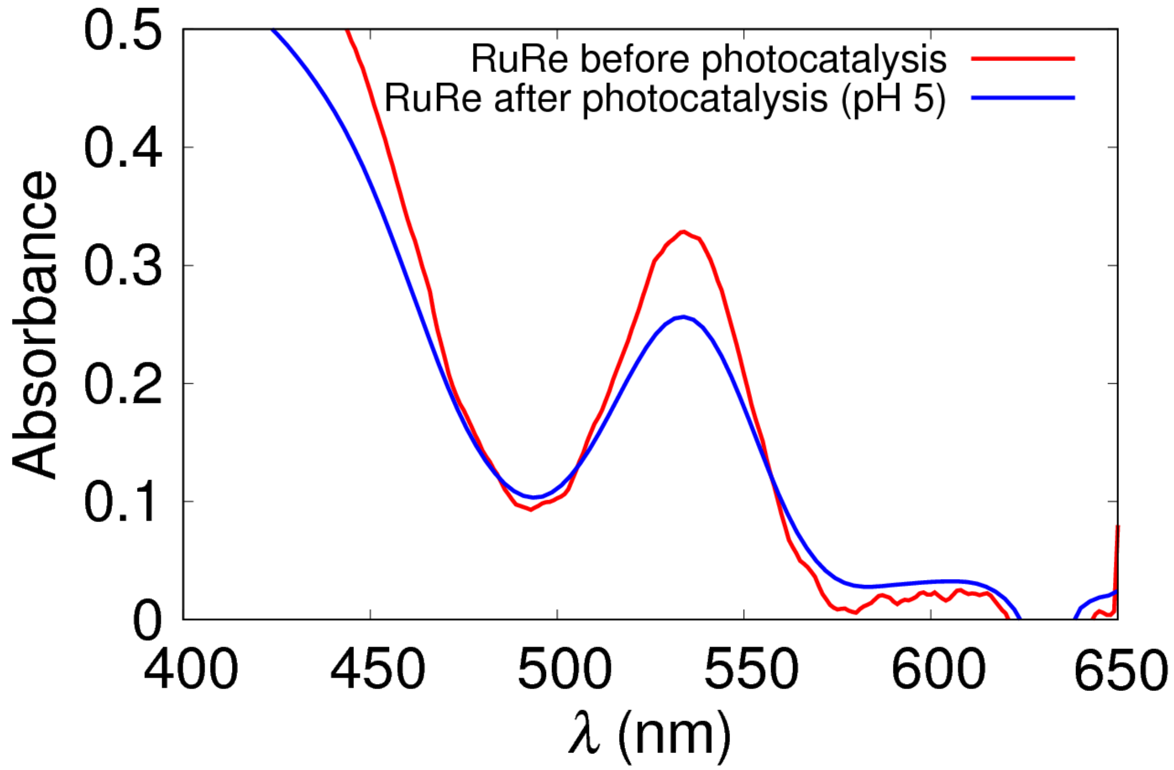

Figure S29. UV-visible absorption spectra of **RuRe** on mesoporous NiO thin films before (red) and after (blue) photocatalysis in pH 5 acetate buffer. For the raw data, please reference https://doi.org/10.25405/data.ncl.c.5696935.v1.

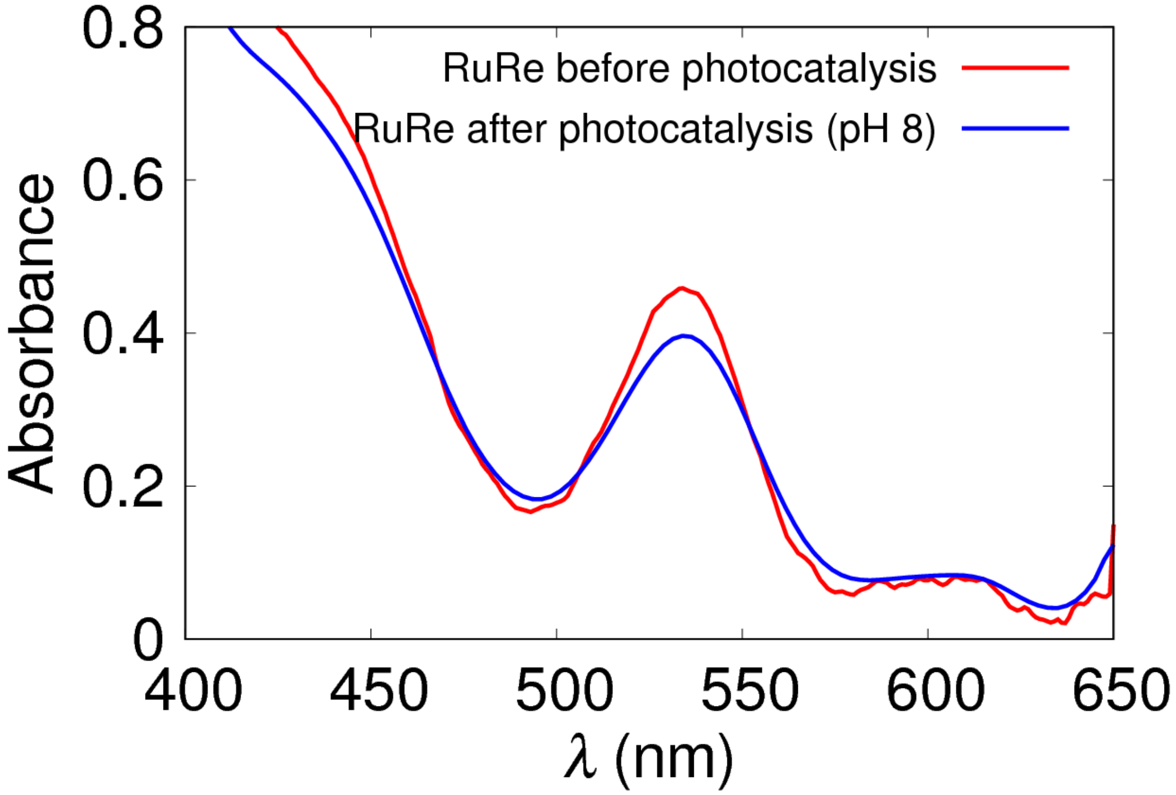

Figure S30. UV-visible absorption spectra of **RuRe** on mesoporous NiO thin films before (red) and after (blue) photocatalysis in pH 8 acetate buffer. For the raw data, please reference https://doi.org/10.25405/data.ncl.c.5696935.v1.

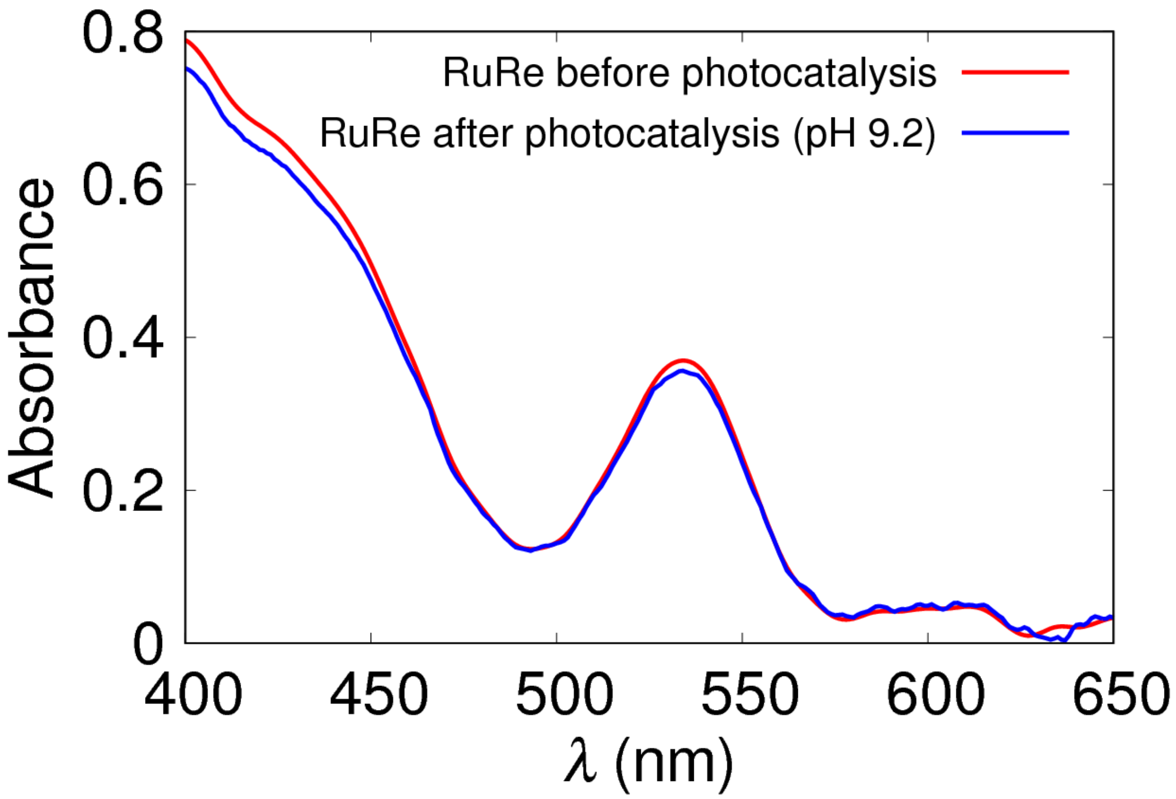

Figure S31. UV-visible absorption spectra of **RuRe** on mesoporous NiO thin films before (red) and after (blue) photocatalysis in pH 9.2 acetate buffer. For the raw data, please reference https://doi.org/10.25405/data.ncl.c.5696935.v1.

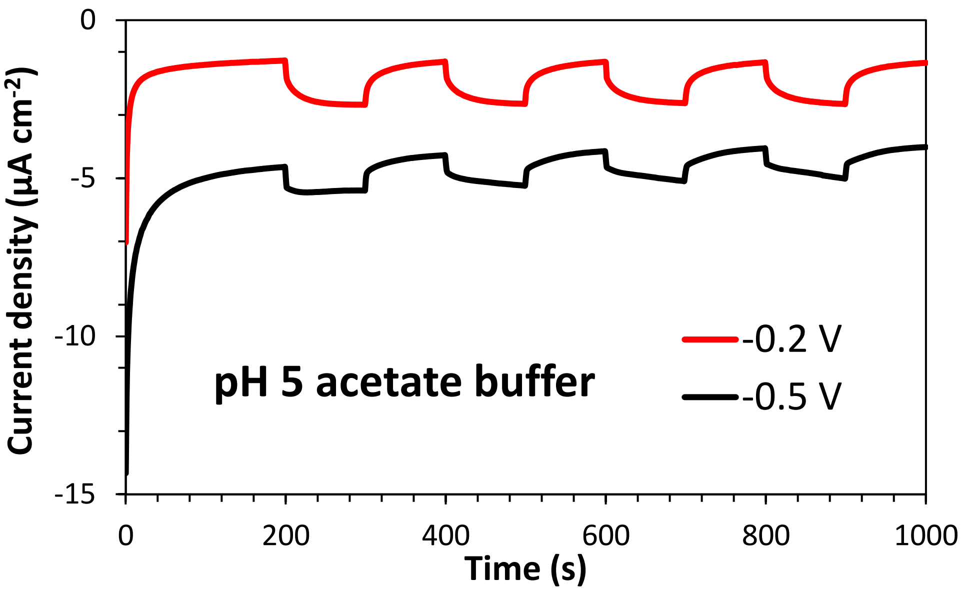

Figure S32. Chronoamperometry measurement for **RuRe** on NiO in pH 5 acetate buffer at different applied bias, under chopped 1 sun illumination. The solution was saturated with CO_2 before starting the experiment.

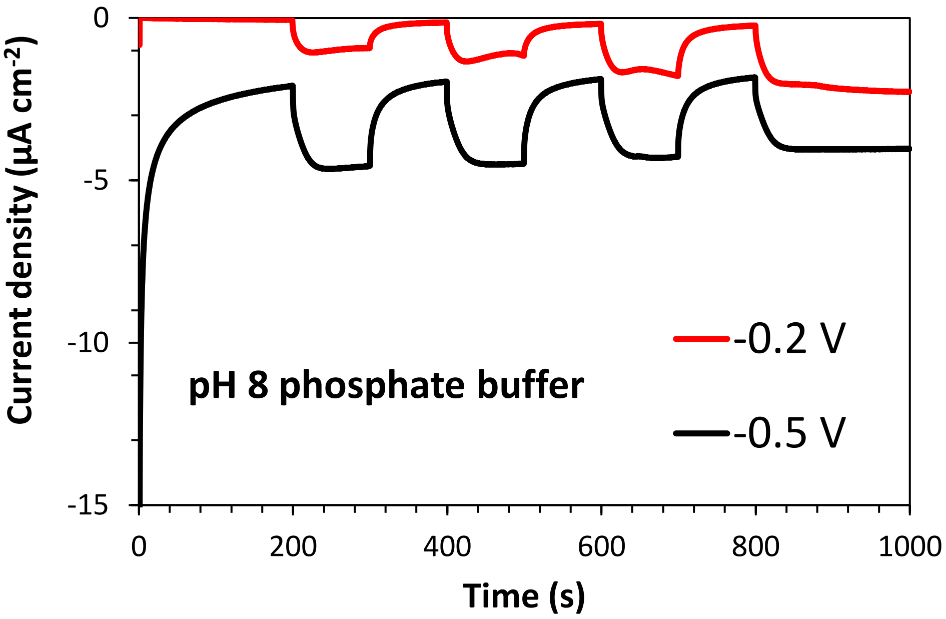

Figure S33. Chronoamperometry measurement for **RuRe** on NiO in pH 8 phosphate buffer at different applied bias, under chopped 1 sun illumination. The solution was saturated with CO_2 before starting the experiment.

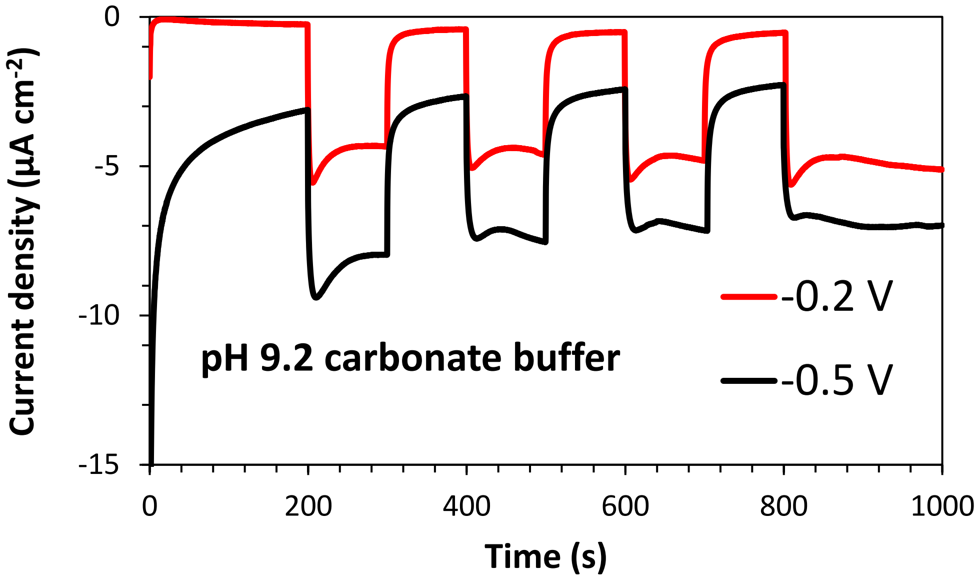

Figure S34. Chronoamperometry measurement for **RuRe** on NiO in pH 9.2 carbonate buffer at different applied bias, under chopped 1 sun illumination. The solution was saturated with CO_2 before starting the experiment.

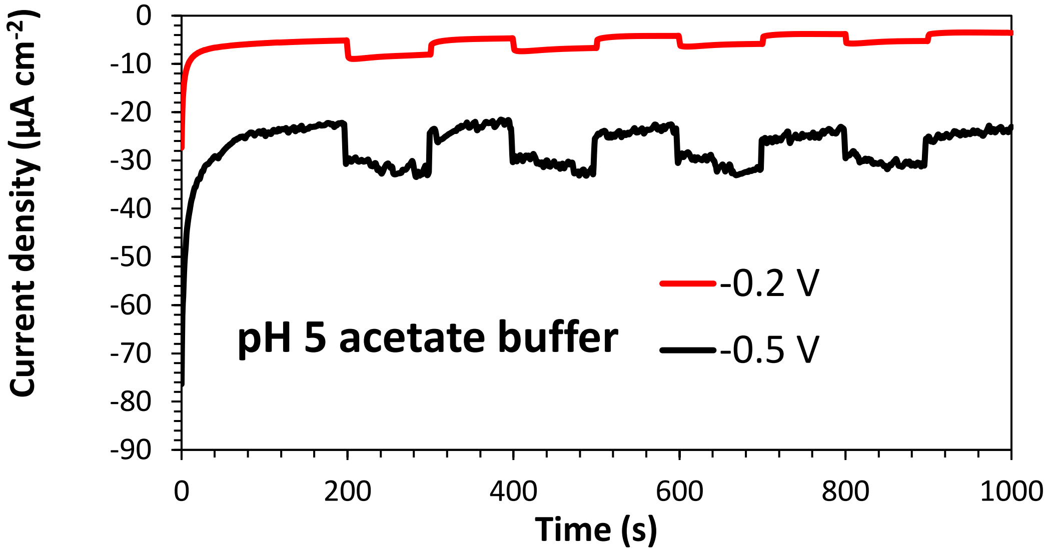

Figure S35. Chronoamperometry measurement for **RuPt** on NiO in pH 5 acetate buffer at different applied bias, under chopped 1 sun illumination. The solution was saturated with argon before starting the experiment.

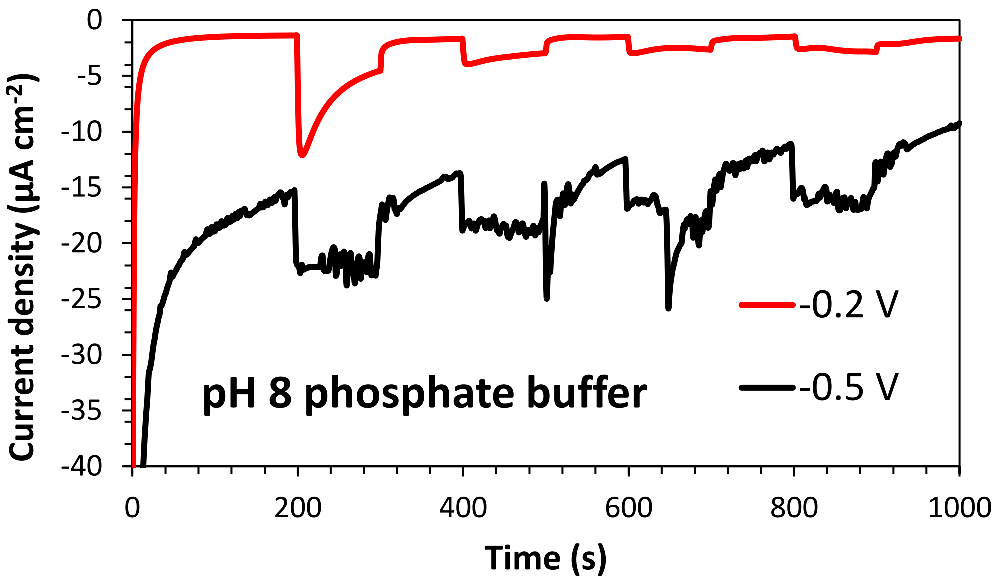

Figure S36. Chronoamperometry measurement for **RuPt** on NiO in pH 8 phosphate buffer at different applied bias, under chopped 1 sun illumination. The solution was saturated with argon before starting the experiment.

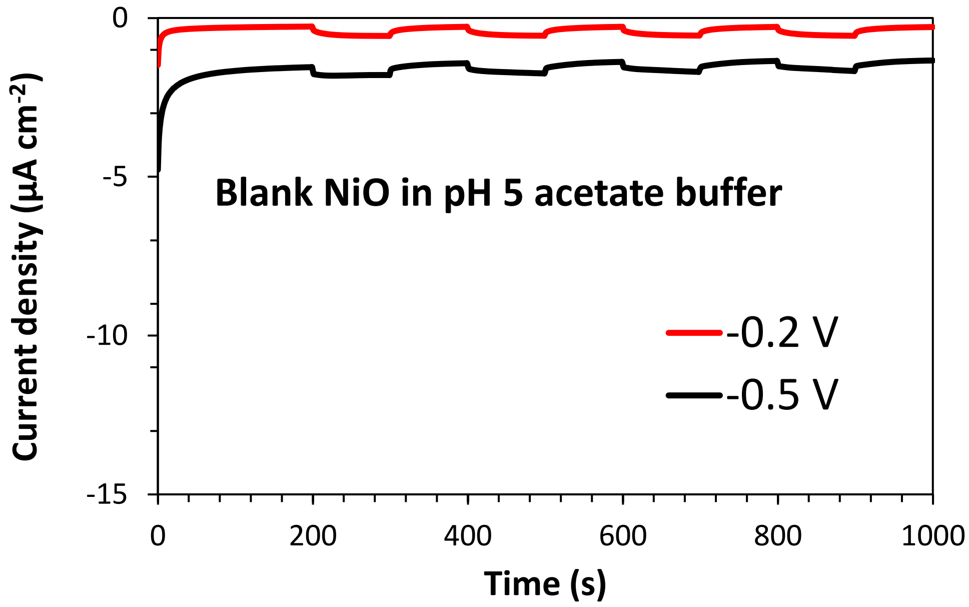

Figure S37. Chronoamperometry measurement for an unsensitized NiO film in pH 5 acetate buffer at different applied bias, under chopped 1 sun illumination. The solution was saturated with argon before starting the experiment.

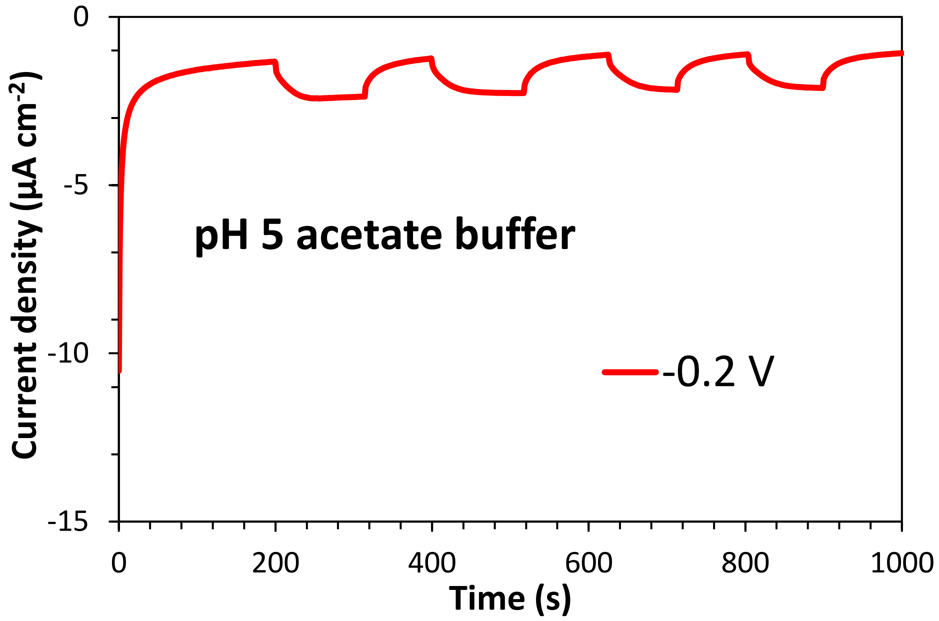

Figure S38. Chronoamperometry measurement for **Ru** on NiO in pH 5 acetate buffer at under a bias of -0.2 V, under chopped 1 sun illumination. Note that at higher applied bias the sample became unstable. The solution was saturated with argon before starting the experiment.

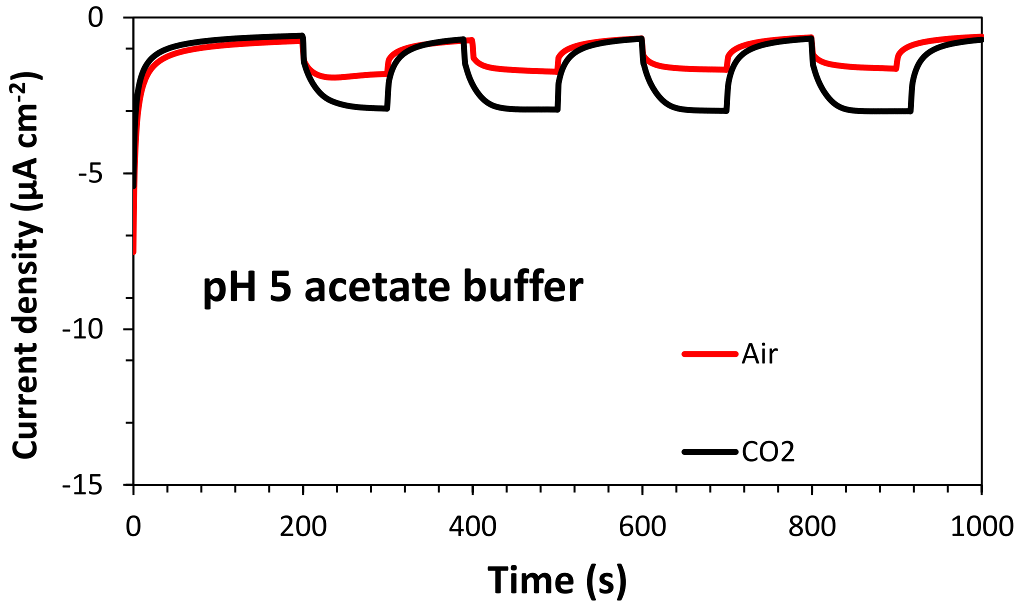

Figure S39. Chronoamperometry measurement for **RuRe** on NiO in pH 5 acetate buffer under chopped 1 sun illumination, both in the presence of air and in saturated with CO_2.

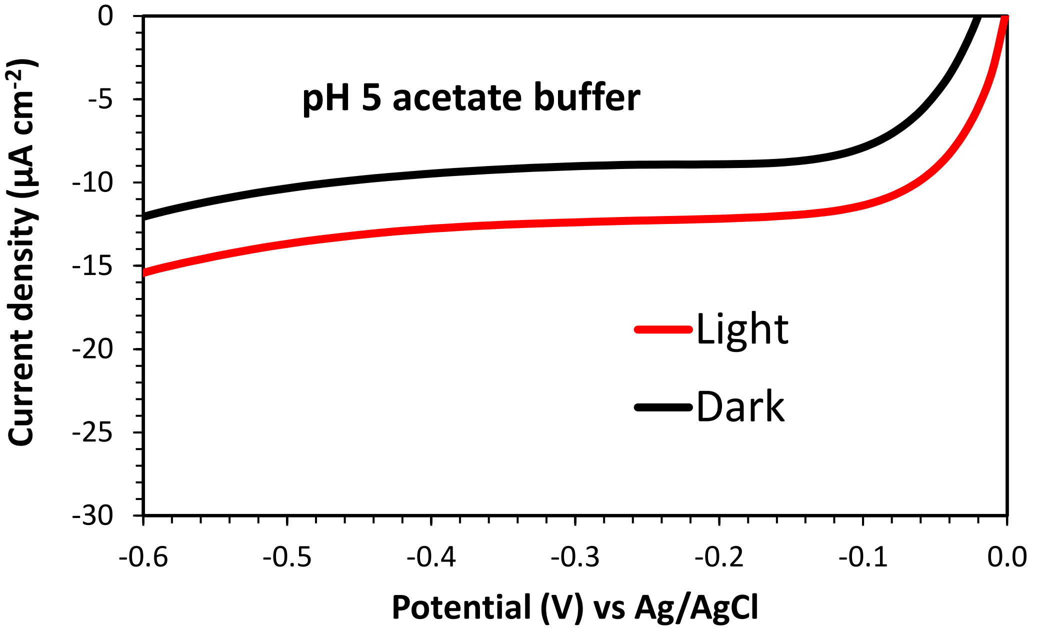

Figure S40. Linear sweep scan for **RuRe** on NiO in pH 5 acetate buffer both under simulated 1 sun illumination and in the dark. The scan speed is 0.1 Vs^{-1}. The solution was saturated with CO_2 before starting the experiment.

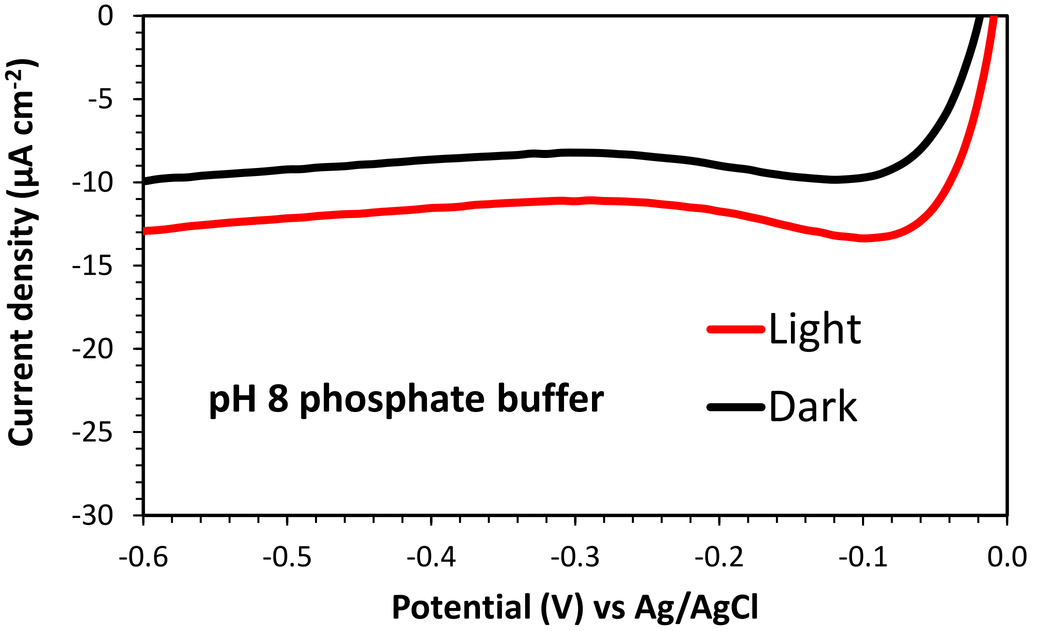

Figure S41. Linear sweep scan for **RuRe** on NiO in pH 8 phosphate buffer both under simulated 1 sun illumination and in the dark. The scan speed is 0.1 Vs^{-1}. The solution was saturated with CO_2 before starting the experiment.

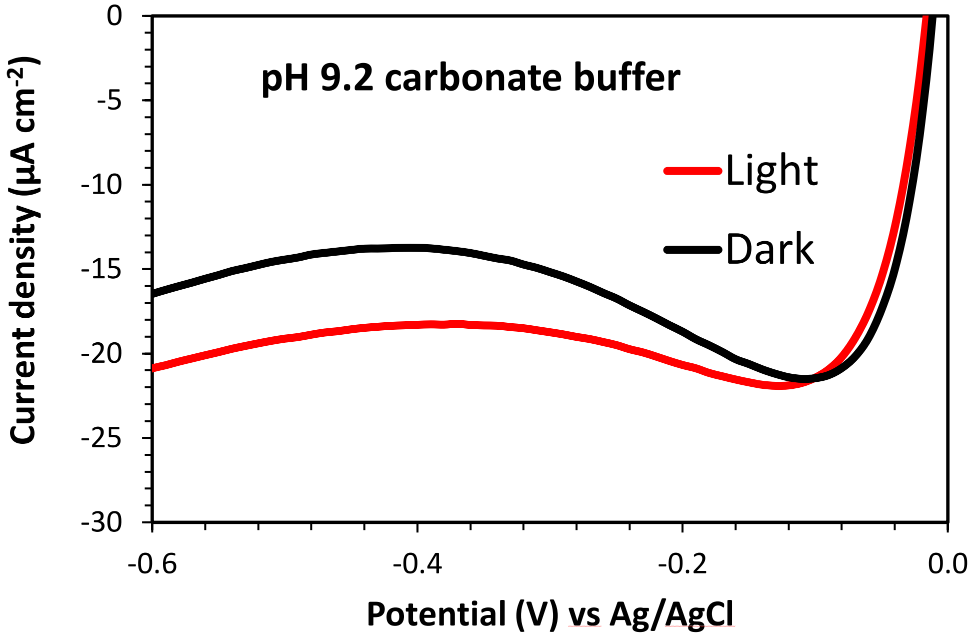

Figure S42. Linear sweep scan for **RuRe** on NiO in pH 9.2 carbonate buffer both under simulated 1 sun illumination and in the dark. The scan speed is 0.1 Vs^{-1}. The solution was saturated with CO_2 before starting the experiment.

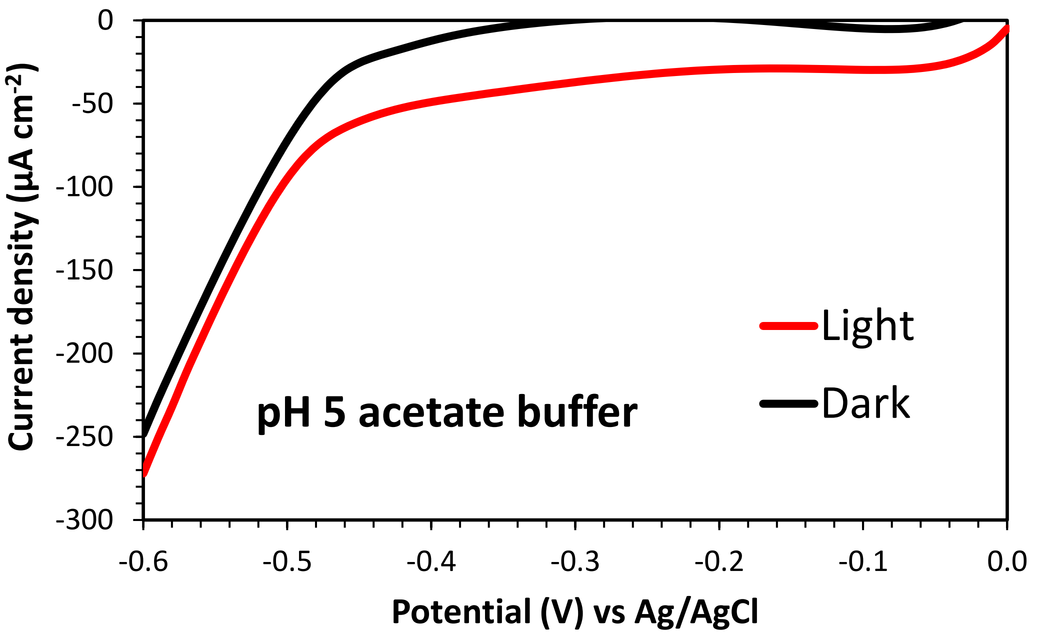

Figure S43. Linear sweep scan for **RuPt** on NiO in pH 5 acetate buffer both under simulated 1 sun illumination and in the dark. The scan speed is 0.1 Vs^{-1}. The solution was saturated with argon before starting the experiment.

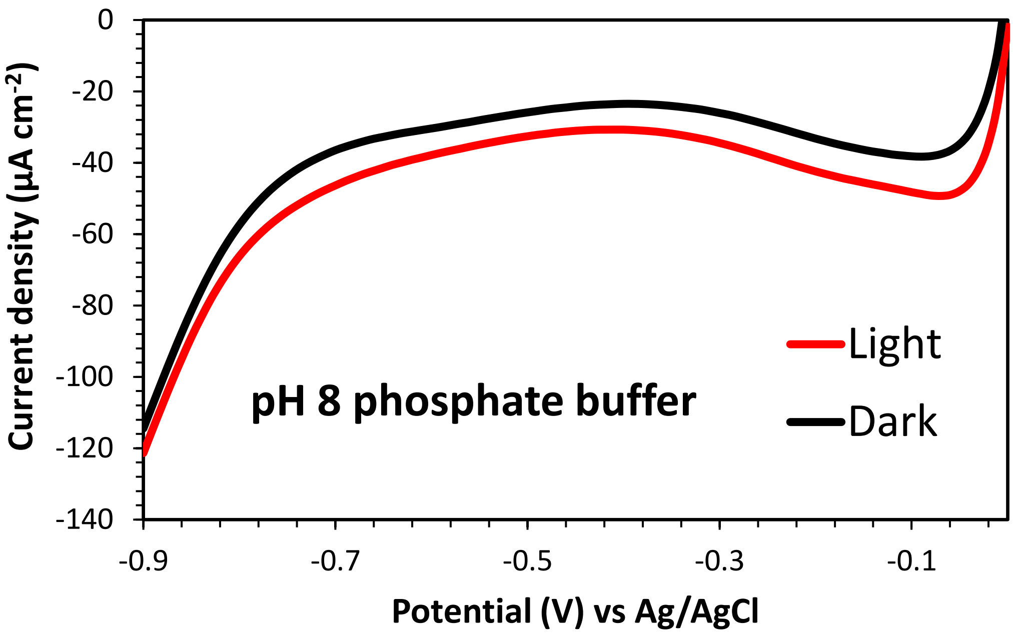

Figure S44. Linear sweep scan for **RuPt** on NiO in pH 8 phosphate buffer both under simulated 1 sun illumination and in the dark. The scan speed is 0.1 Vs^{-1}. The solution was saturated with argon before starting the experiment.

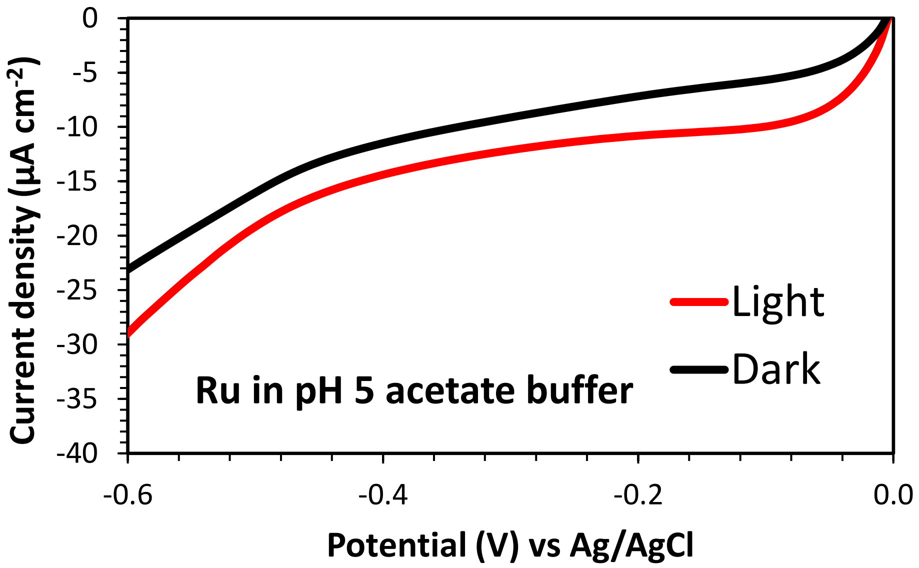

Figure S45. Linear sweep scan for **Ru** on NiO in pH 5 acetate buffer both under simulated 1 sun illumination and in the dark. The scan speed is 0.1 Vs^{-1}. The solution was saturated with argon before starting the experiment.

Gas Chromatography Measurements

Gas chromatography measurements were performed with a Shimadzu GC-2014 instrument where a thermal conductivity detector at 80 ^oC (TCD) and flame-ionisation detector (FID) at 150 ^oC operated in tandem. A Restek ShinCarbon ST packed column (2 metre, 2 mm ID) was used where argon was used as the carrier gas, and the oven temperature was 50 ^oC. For determining locations of key product peaks and for calibration purposes a standard gas mixture containing 5000 ppm propylene, 5000 ppm ethane, 5000 ppm ethylene, 5000 ppm methane, 1% carbon monoxide, 1 % carbon dioxide, and the balance carbon dioxide (BOC).

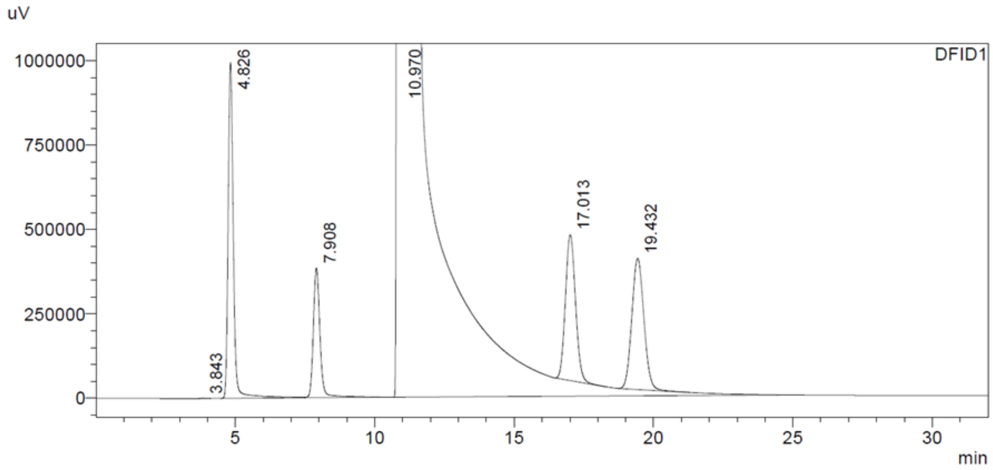

Peak#	Ret. Time	Area	Height
1	3.843	9244	762
2	4.826	13006030	994363
3	7.908	6459451	384652
4	10.970	##########	47222205
5	17.013	10927461	432330
6	19.432	11770190	388606
Total		##########	49422918

Figure S46. GC trace of standard calibration gas. Carbon monoxide is at a retention time of 4.826 minutes.

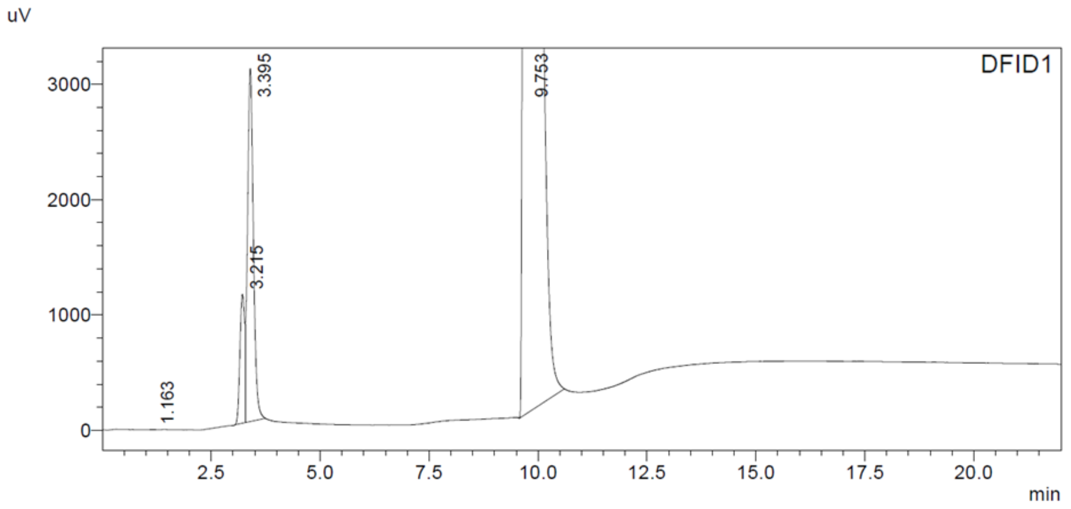

DFID1

Peak#	Ret. Time	Area
1	1.163	3
2	3.215	8283
3	3.395	28787
4	9.753	303255
Total		340328

Figure S47. GC trace of **Ru** on NiO after 1 hour irradiation in pH 5 acetate buffer with an applied bias of -0.2 V under continuous 1 sun AM 1.5 irradiation.

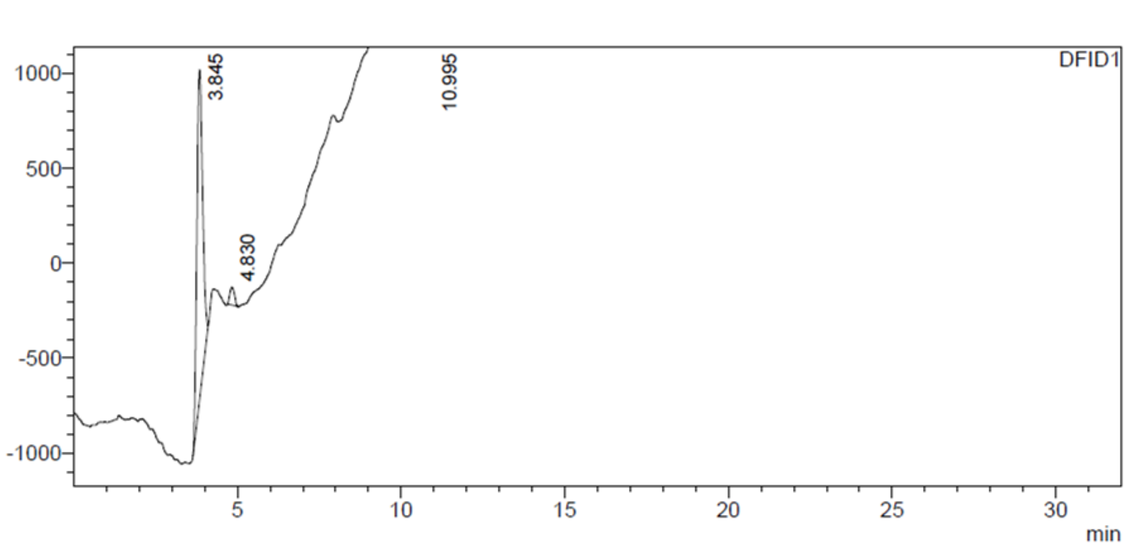

Peak#	Ret. Time	Area	Height
1	3.845	21148	1734
2	4.830	931	95
3	10.995	##########	43863285
Total		##########	43865114

Figure S48. GC trace of **RuRe** on NiO after 1 hour irradiation in pH 5 acetate buffer with an applied bias of -0.2 V under continuous 1 sun AM 1.5 irradiation.

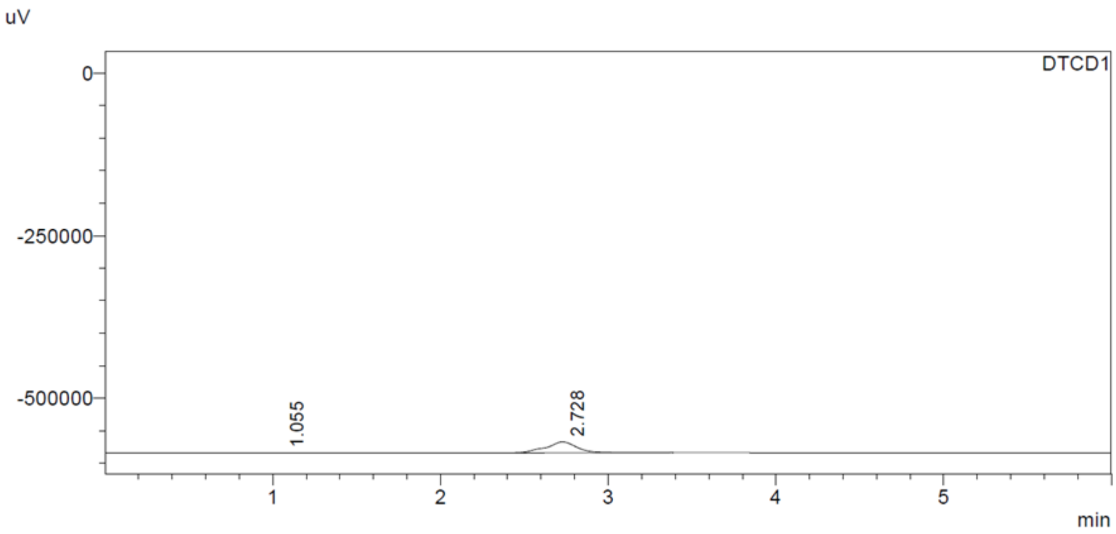

DTCD1

Peak#	Ret. Time	Area
1	1.055	14
2	2.728	214313
Total		214327

Figure S49. GC trace of **RuPt** on NiO after 1 hour irradiation in pH 5 acetate buffer with an applied bias of -0.2 V under continuous 1 sun AM 1.5 irradiation. The hydrogen peak on the TCD detector is expected at approximately 1 minute.

NMR spectra

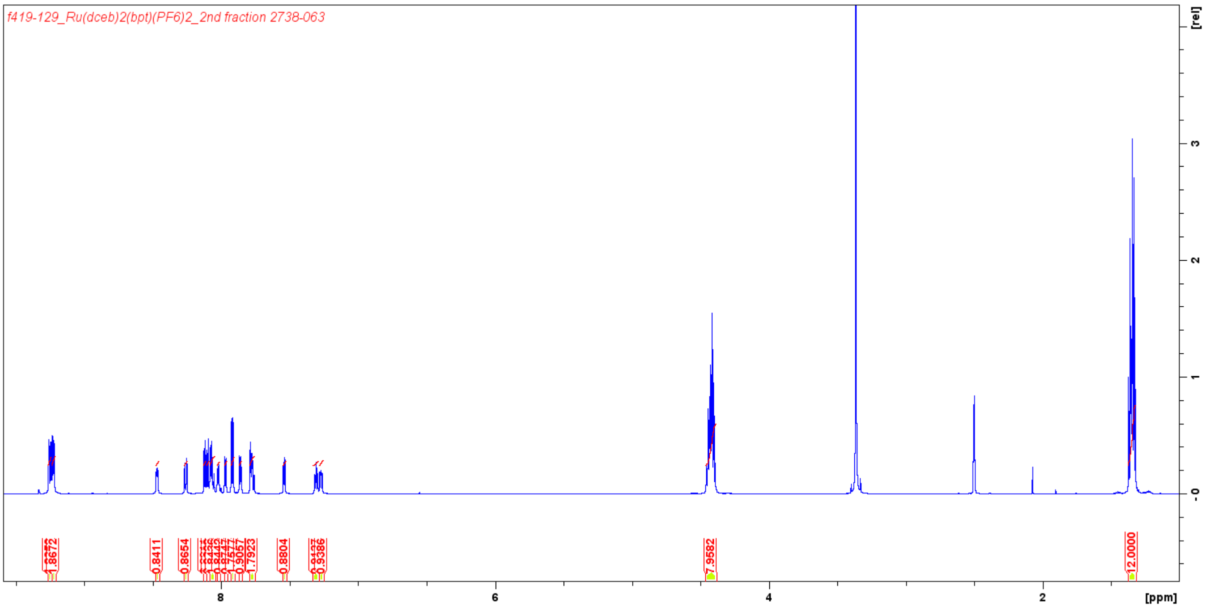

Figure S50. ^{1}H-NMR spectrum of **Ru** in DMSO-d_6.

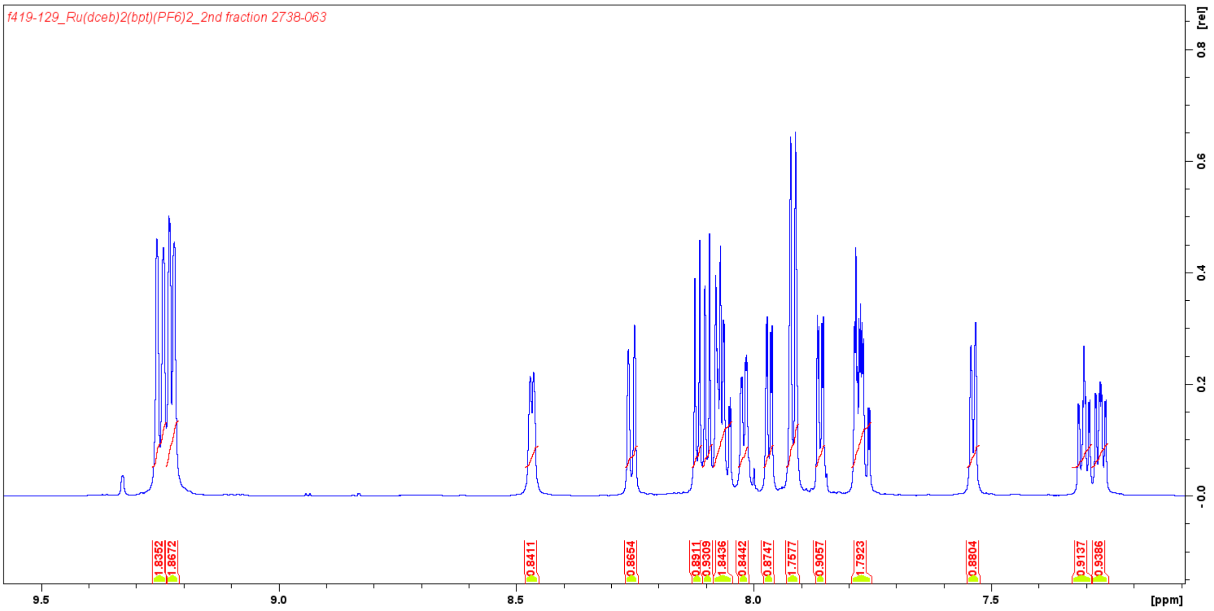

Figure S51. ^{1}H-NMR spectrum of **Ru** in DMSO-d_6, zoom in of the aromatic region.

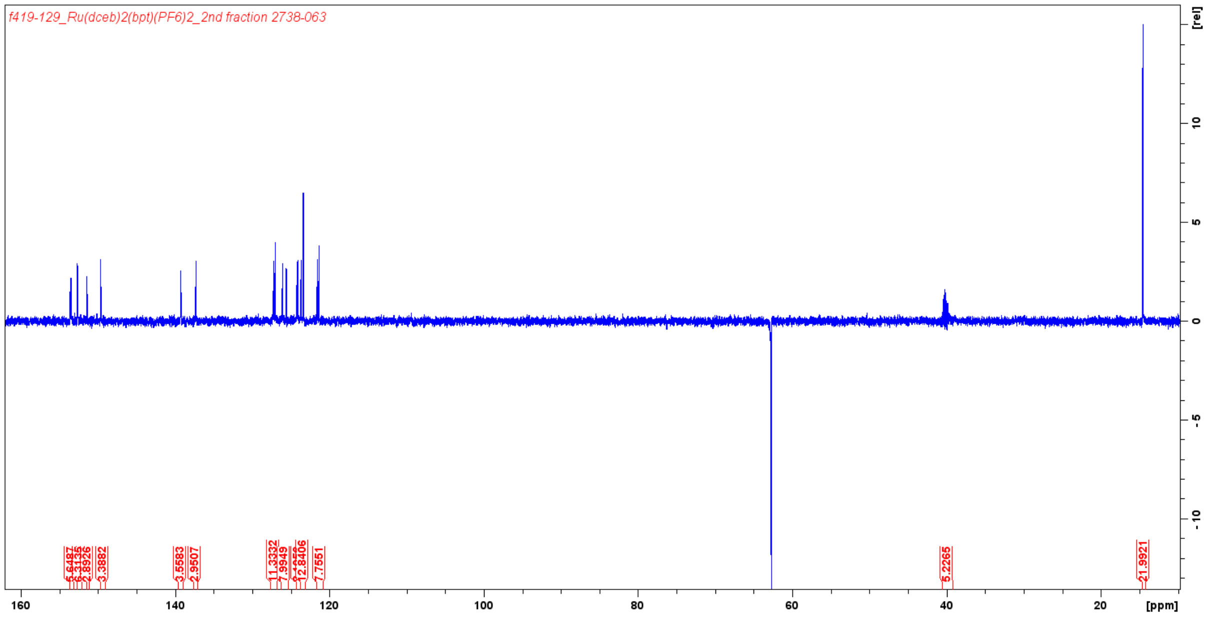

Figure S52. DEPT-135 ^{13}C-NMR spectrum of **Ru** in DMSO-d6.

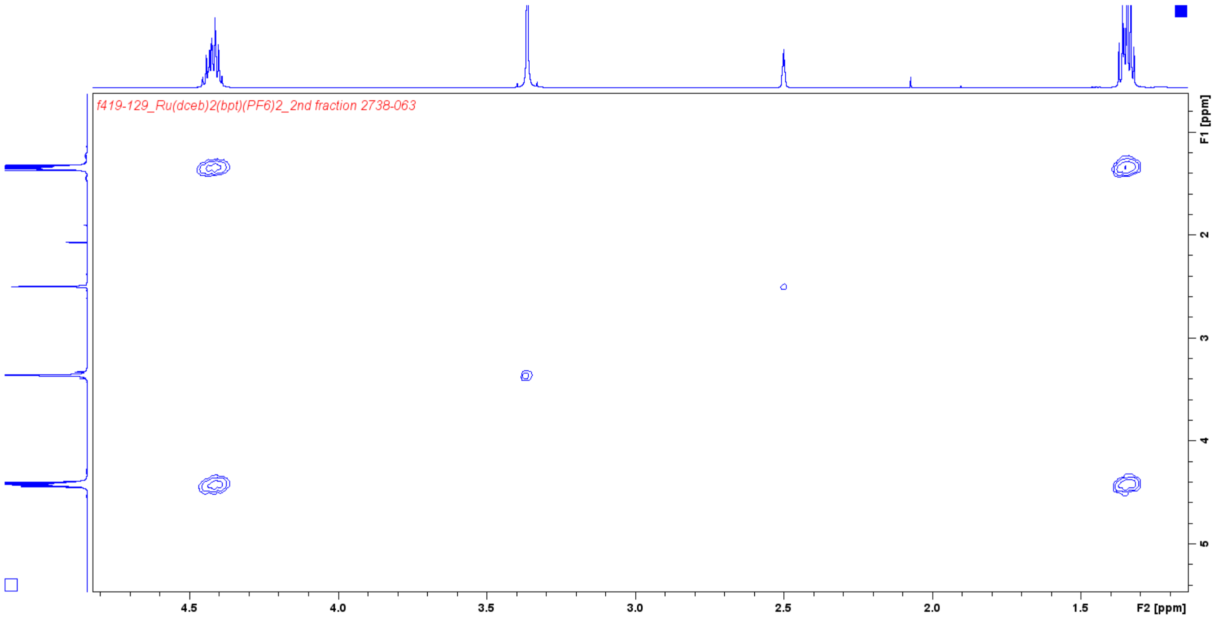

Figure S53. COSY spectrum of Ru in DMSO-d6. 0-5 ppm range.

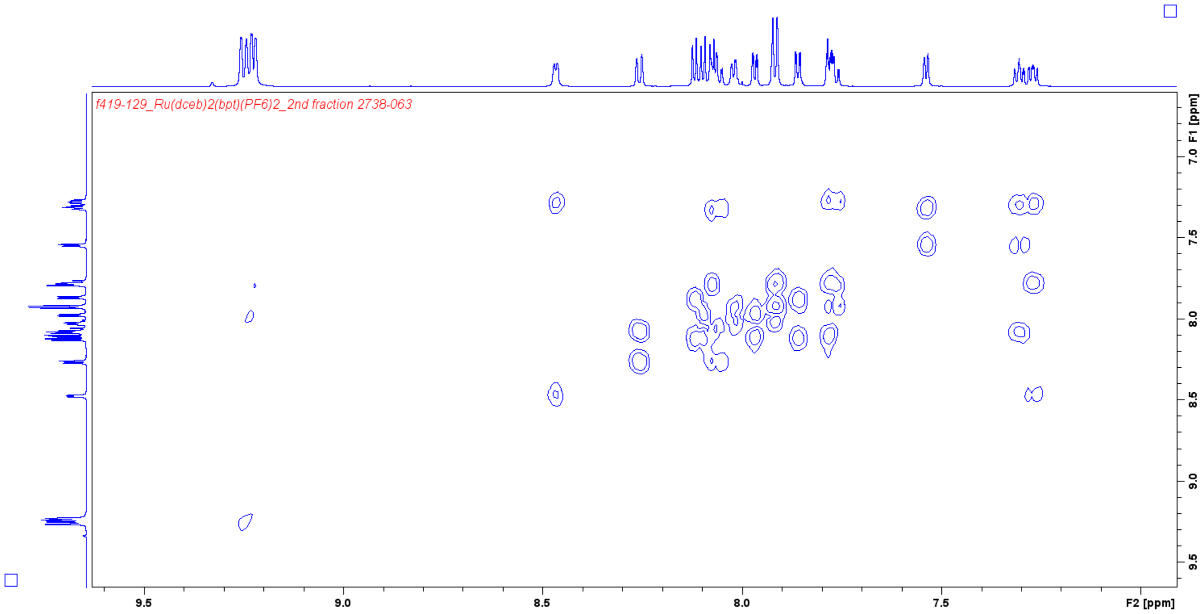

Figure S54. COSY NMR spectrum of **Ru** in DMSO-d$_6$ if the 6-9 ppm area.

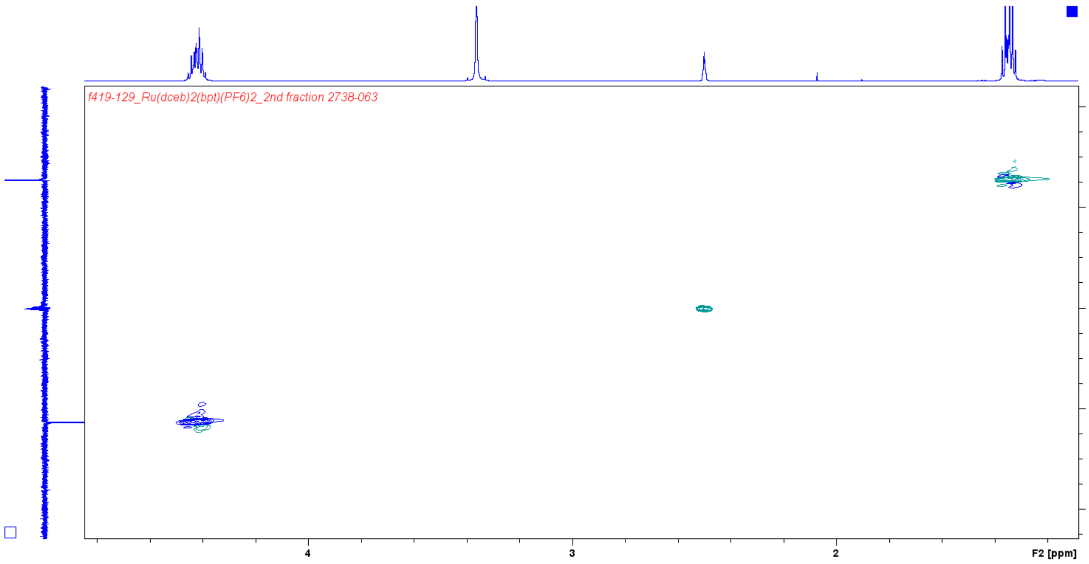

Figure S55. HSQC NMR spectrum of **Ru** in DMSO-d$_6$ focussed on the 0-5 ppm area. The ^{1}H-nmr spectrum (top axis) and ^{13}C-135-DEPT spectrum (left axis) are shown for clarity.

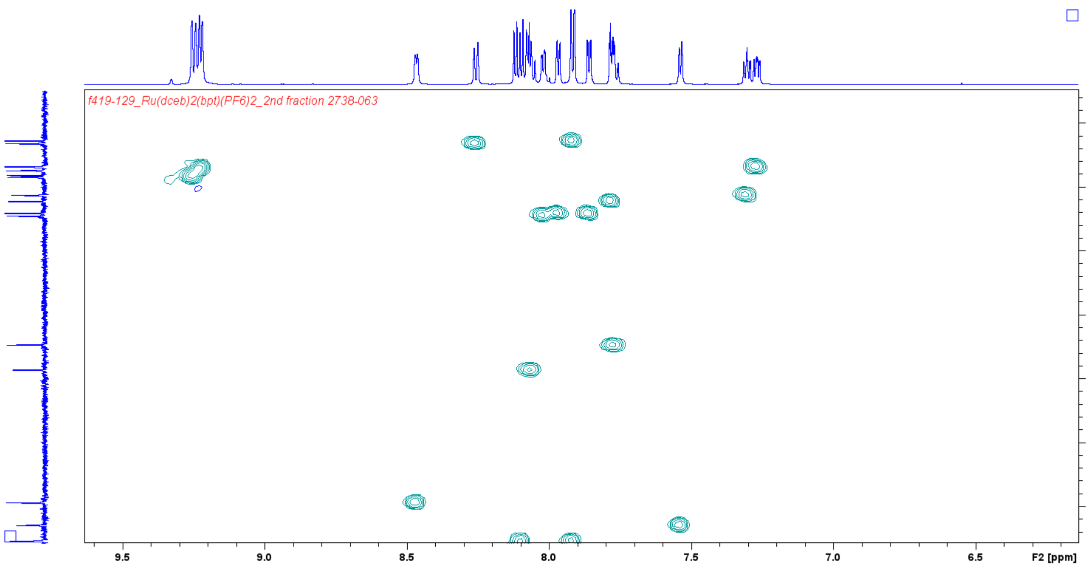

Figure S56. NMR HSQC spectrum of **Ru** in DMSO-d_6 focussed on the 6-10 ppm area. The ^{1}H-nmr spectrum (top axis) and ^{13}C-135-DEPT spectrum (left axis) are shown for clarity.

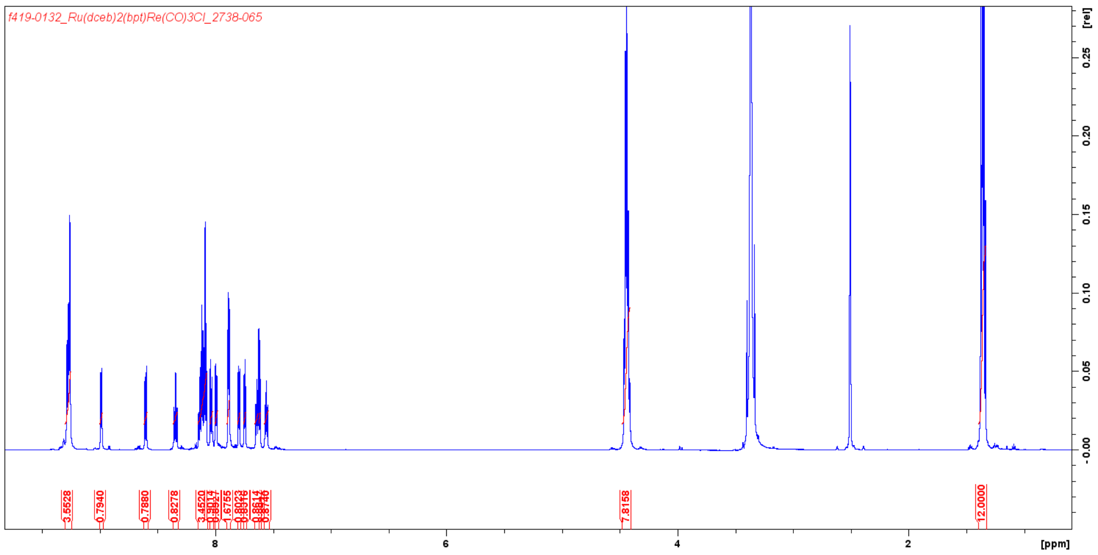

Figure S57. ^{1}H-NMR spectrum of **RuRe** in DMSO-d_6. The signal at 3.3 ppm is residual water.

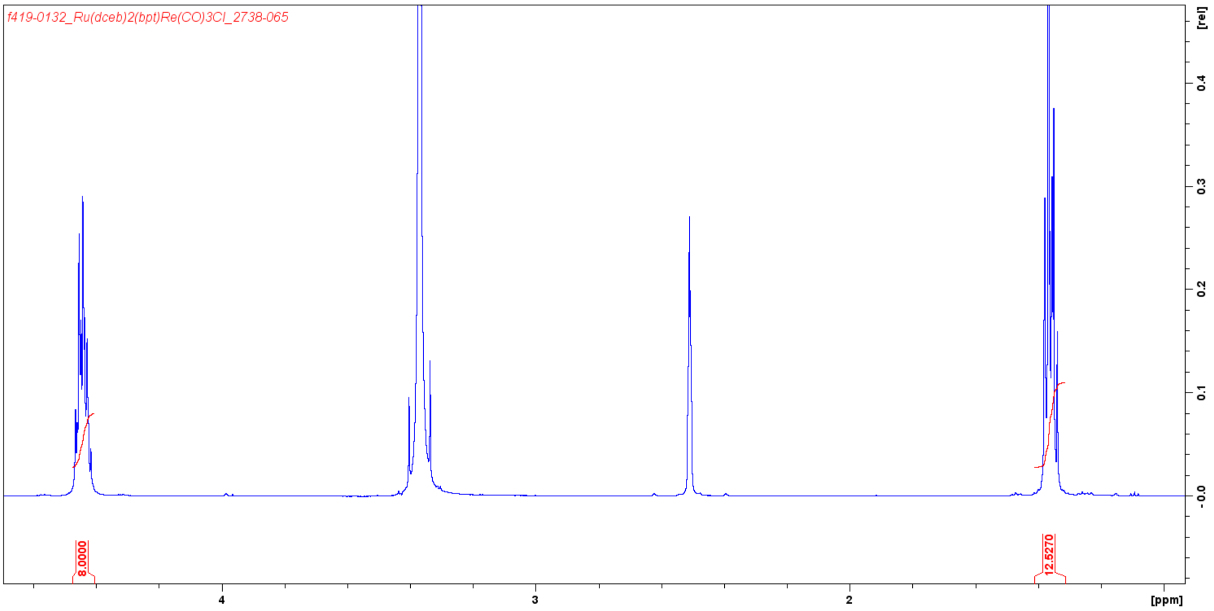

Figure S58 ^{1}H-NMR spectrum of **RuRe** in DMSO-d$_6$, zoom in of the aliphatic region.

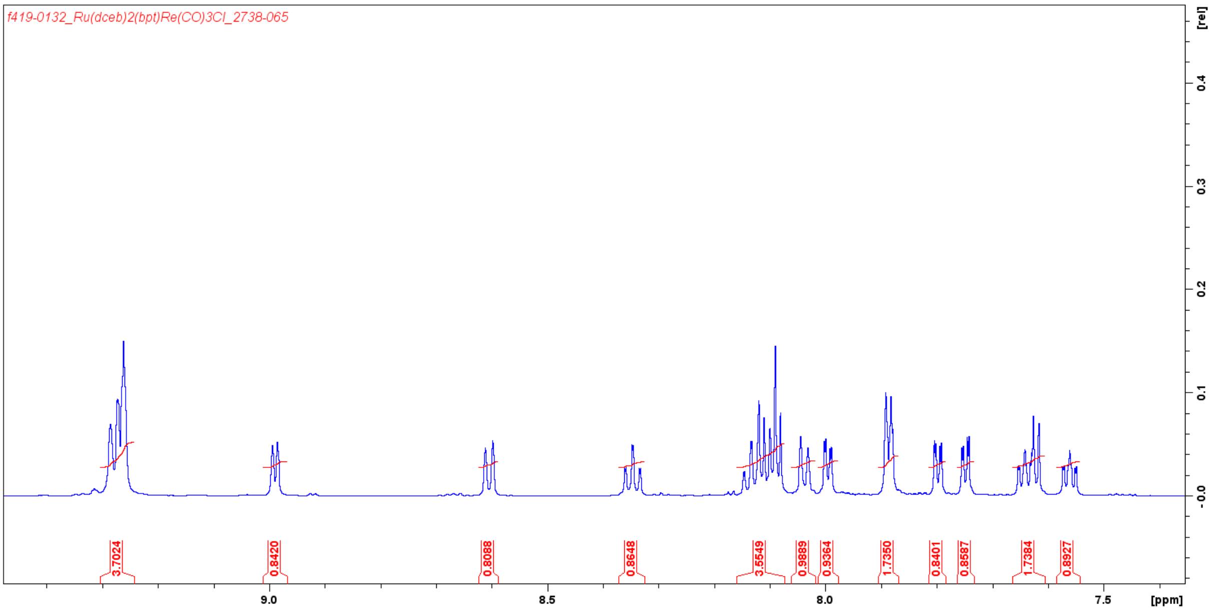

Figure S59. ^{1}H-NMR spectrum of **RuRe** in DMSO-d$_6$, zoom in of the aromatic region.

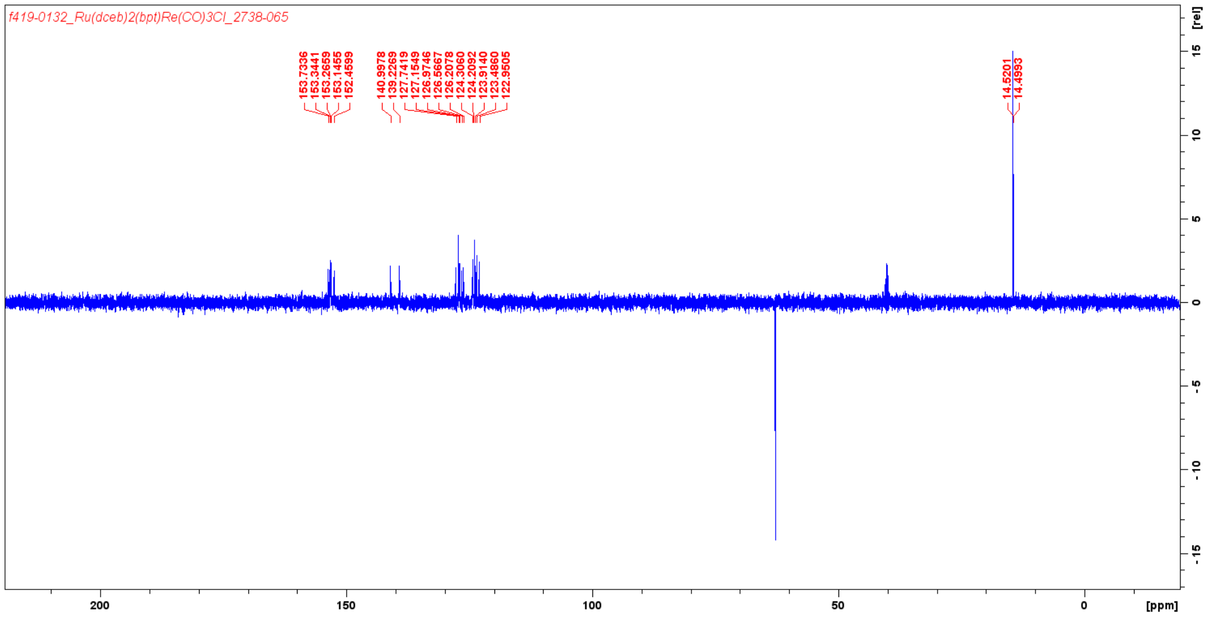

Figure S60. DEPT-135 ^{13}C-NMR spectrum of **RuRe** in DMSO-d$_6$.

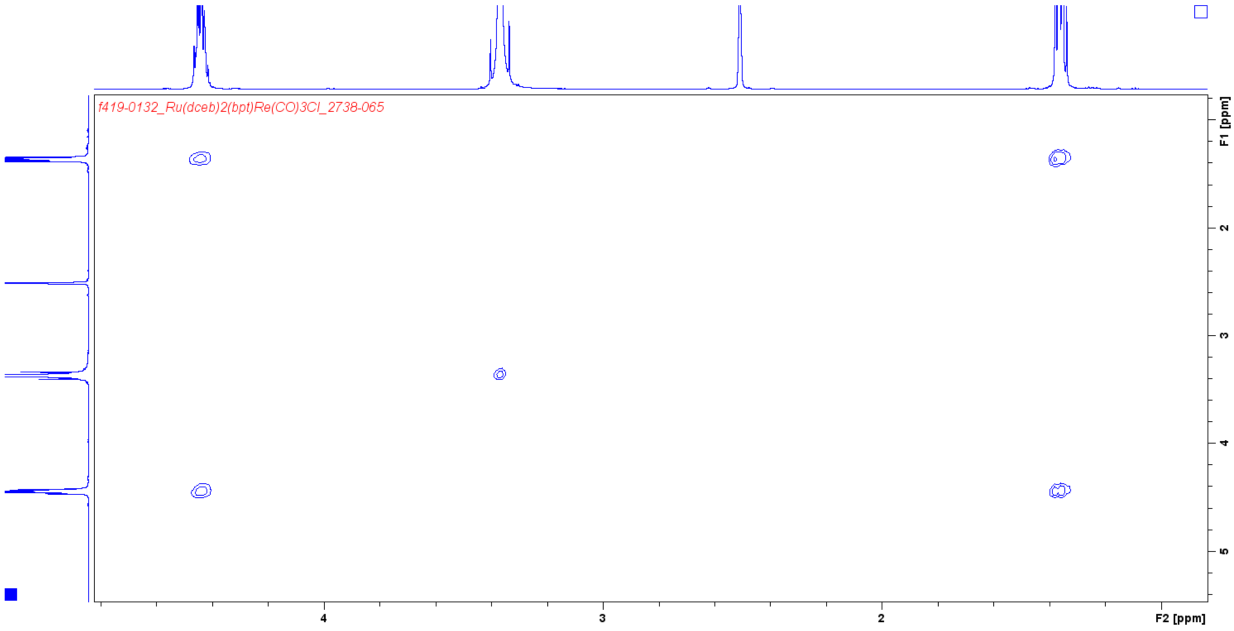

Figure S61. COSY 1H-NMR spectrum of **RuRe** in DMSO-d$_6$ focussed on the 0-5 ppm area.

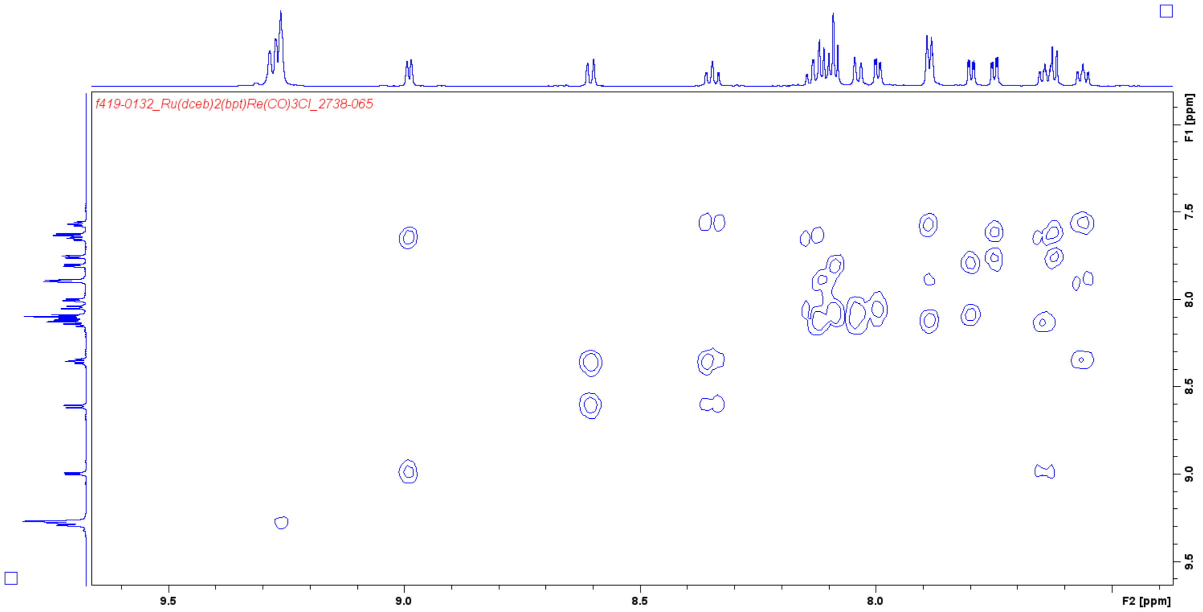

Figure S62. COSY NMR spectrum of **RuRe** in DMSO-d_6 focussed on the 6-9 ppm area.

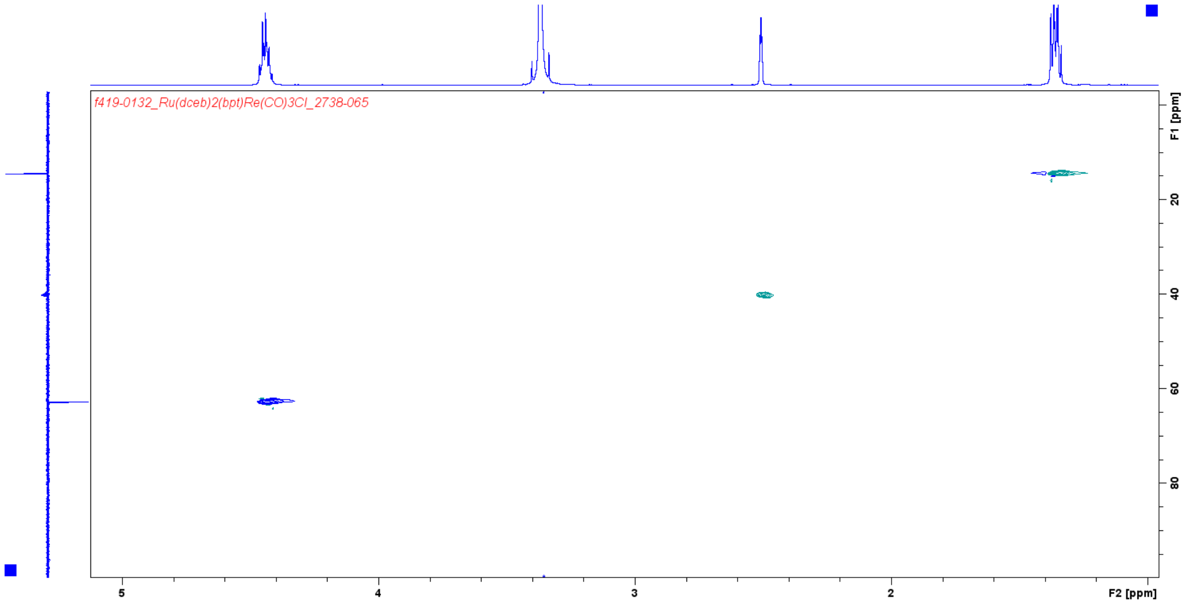

Figure S63. HSQC NMR spectrum of **RuRe** in DMSO-d_6 focussed on the 0-5 ppm area. The ^{1}H-nmr spectrum (top axis) and ^{13}C-135-DEPT spectrum (left axis) are shown for clarity.

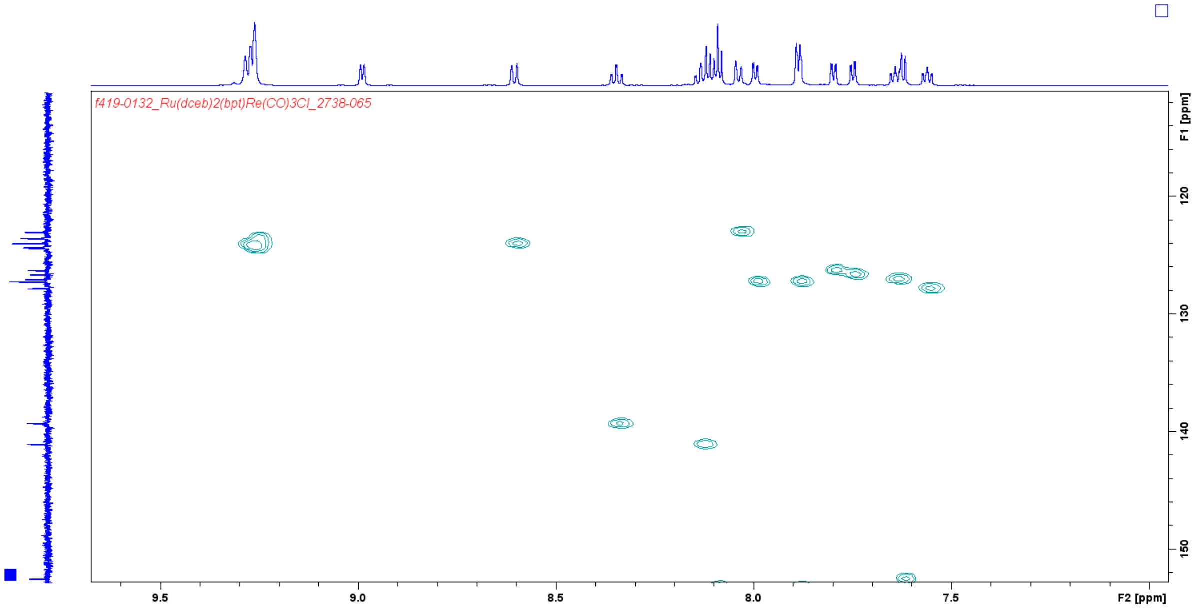

Figure S64. HSQC NMR spectrum of **RuRe** in DMSO-d_6 focussed on the 6-10 ppm area. The ^{1}H-nmr spectrum (top axis) and ^{13}C-135-DEPT spectrum (left axis) are shown for clarity.

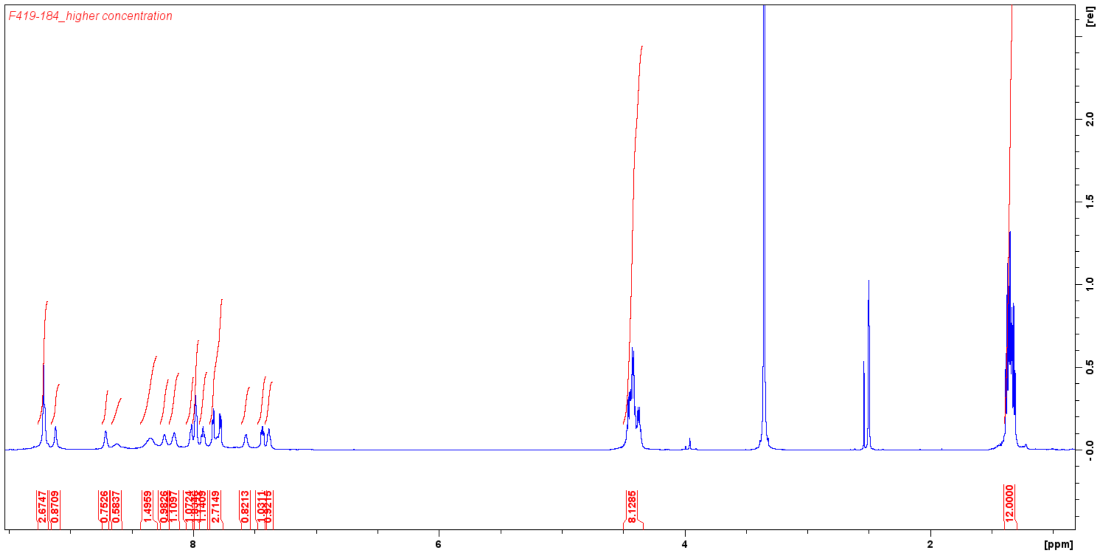

Figure S65. ^{1}H-NMR spectrum of **RuPt** in DMSO-d_6. The signal at 2.54 is residual DMSO. The signal at 3.3 is residual water present in the deuterated solvent.

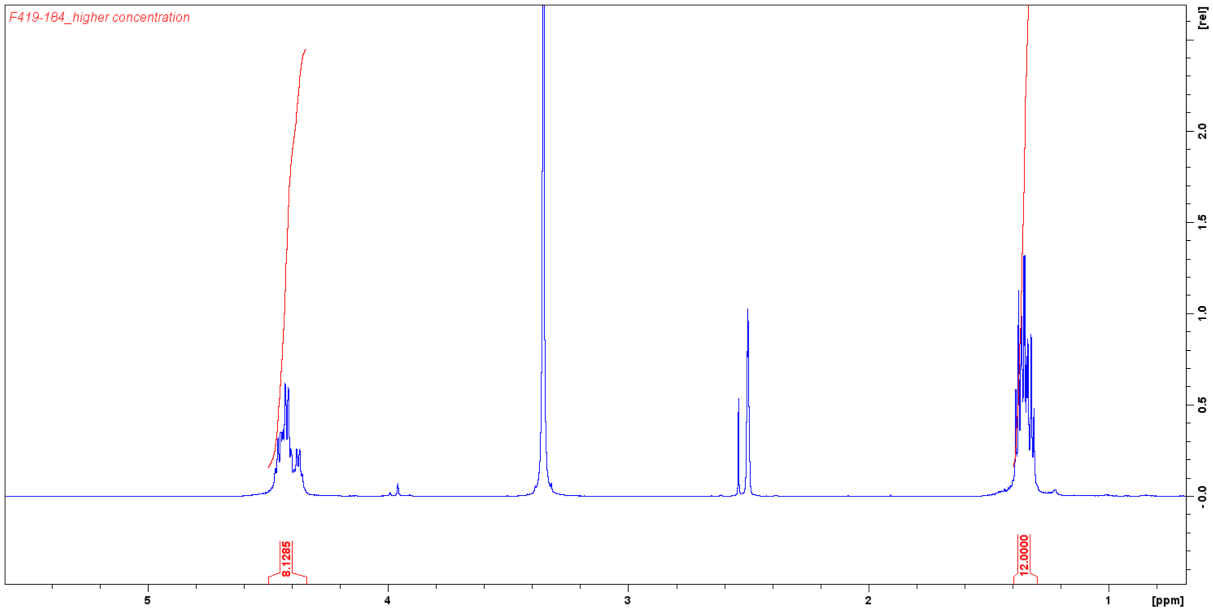

Figure S66 ^{1}H-NMR spectrum of **RuPt** in DMSO-d_6, zoom in of the aliphatic region.

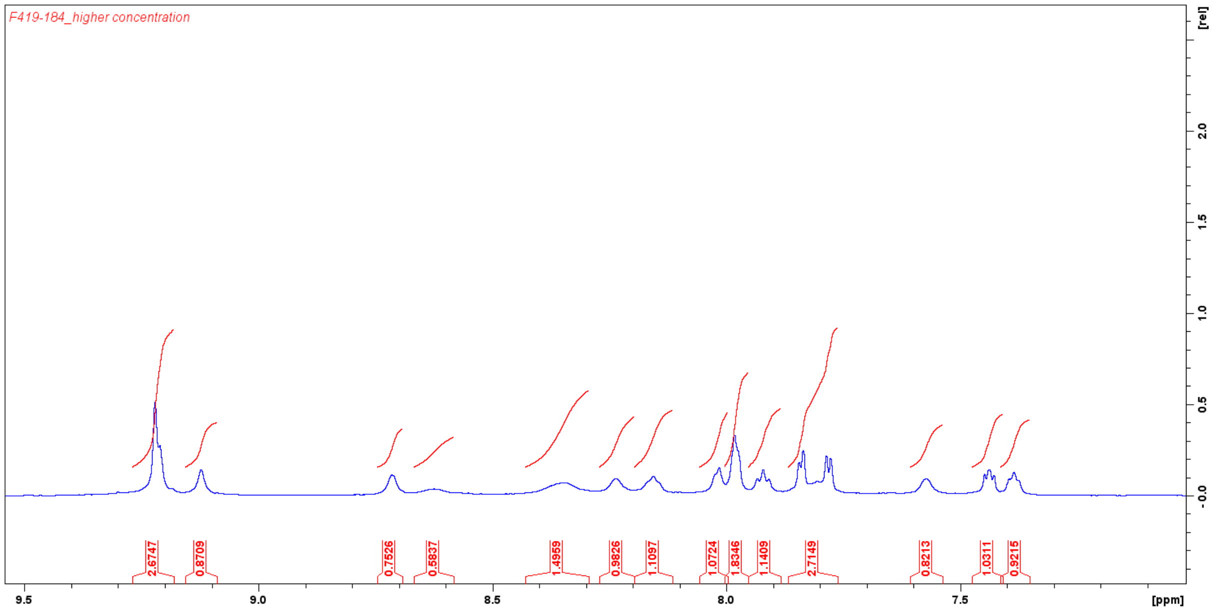

Figure S67 1H-NMR spectrum of **RuPt** in DMSO-d6, zoom in of the aliphatic region.

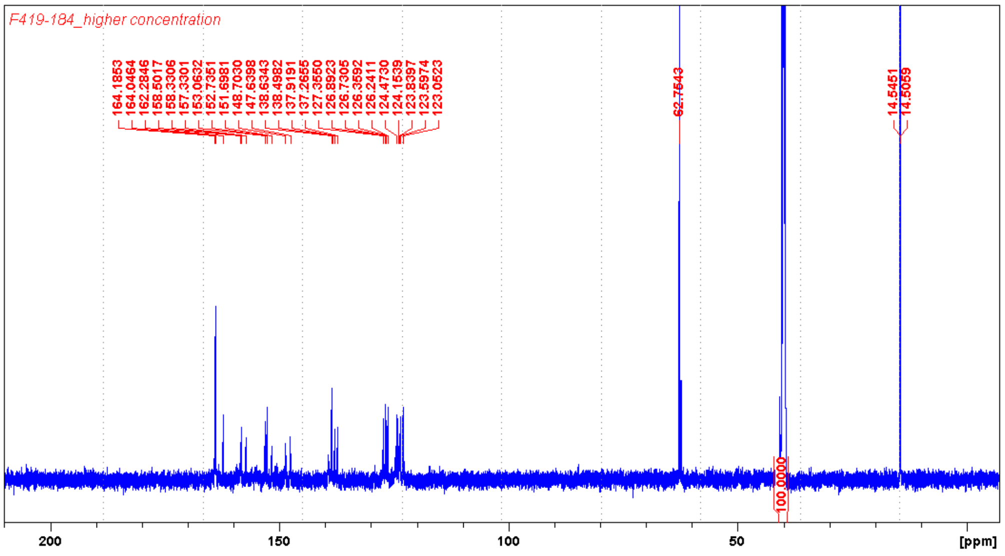

Figure S68. ^{13}C-NMR spectrum of **RuPt** in DMSO-d$_6$. Signals at 40 ppm are residual DMSO.

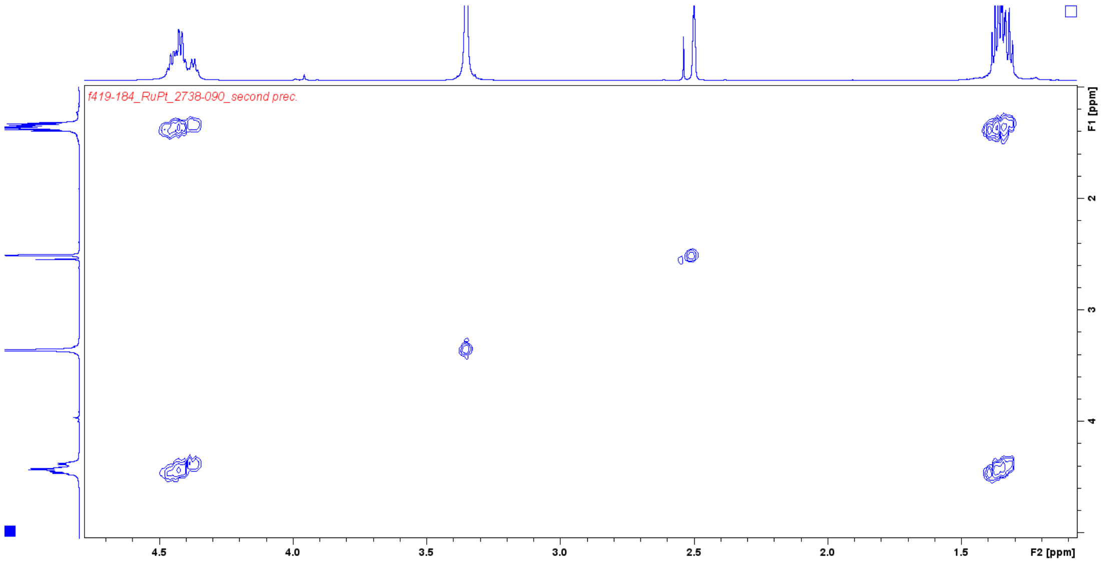

Figure S69. COSY NMR spectrum of **RuPt** in DMSO-d$_6$ focussed on the 0-5 ppm area. Signal at 2.5 and 2.54 are DMSO, signal at 3.3 is residual water.

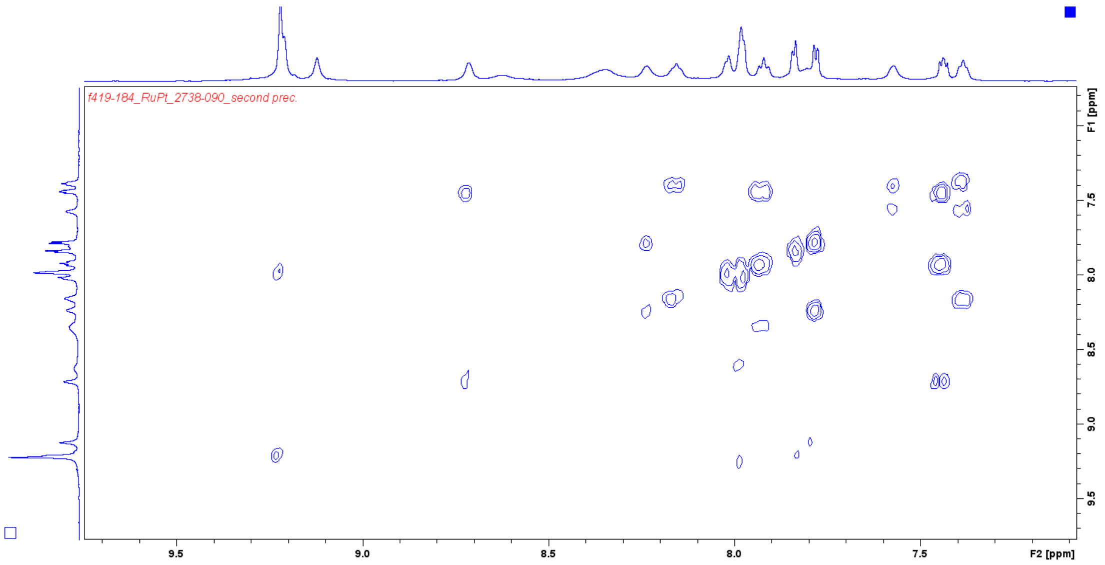

Figure S70. COSY NMR spectrum of **RuPt** in DMSO-d$_6$ focussed on the 6-11 ppm area.

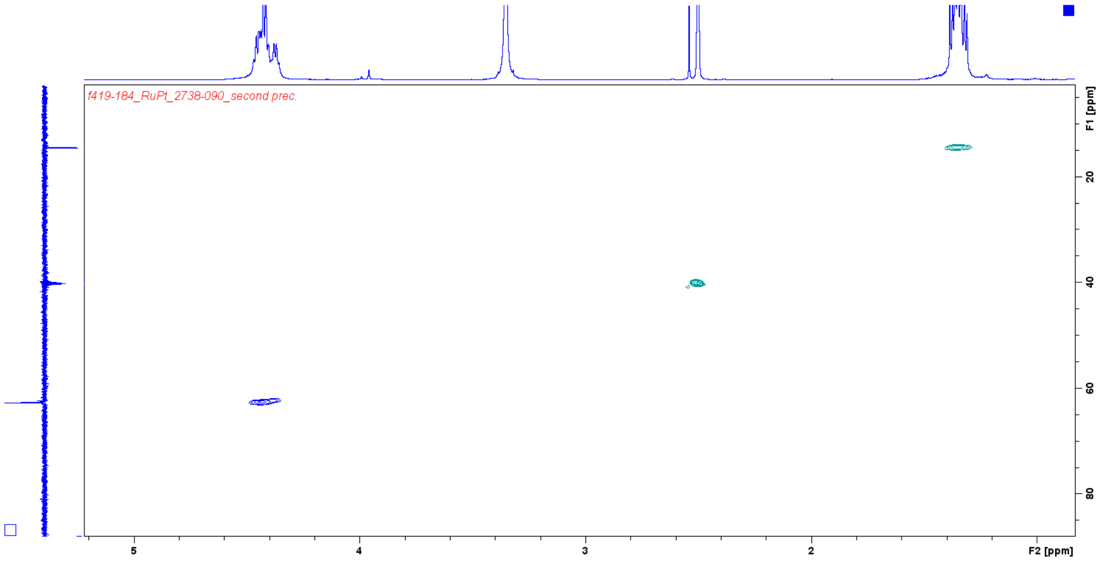

Figure S71. HSQC NMR spectrum of **RuPt** in DMSO-d$_6$ focussed on the 0-5 ppm area.

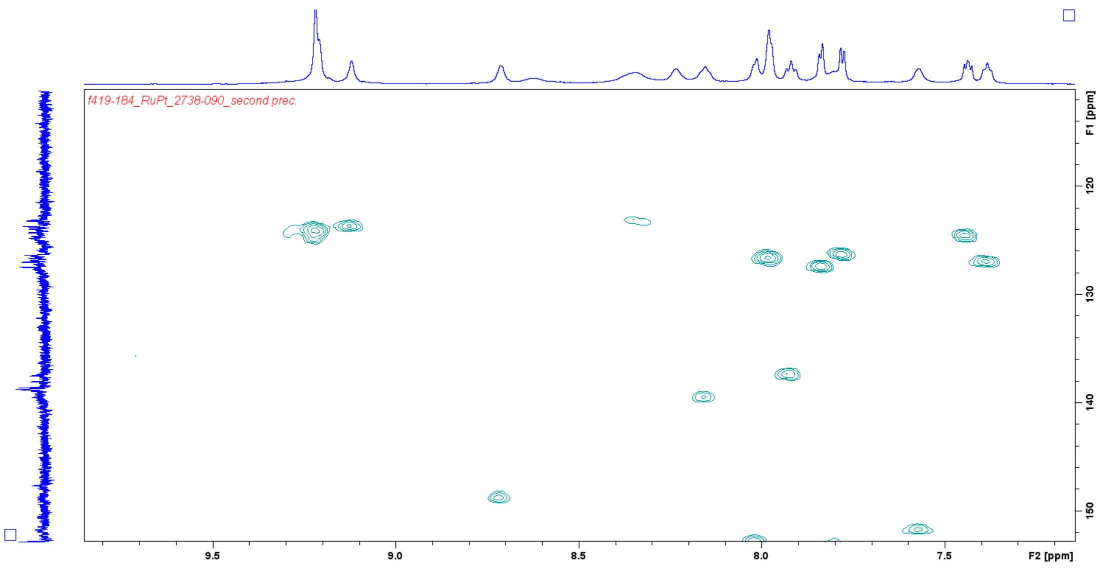

Figure S72. HSQC NMR spectrum of **RuPt** in DMSO-d$_6$ focussed on the 6-10 ppm area.

Mass spectrometry

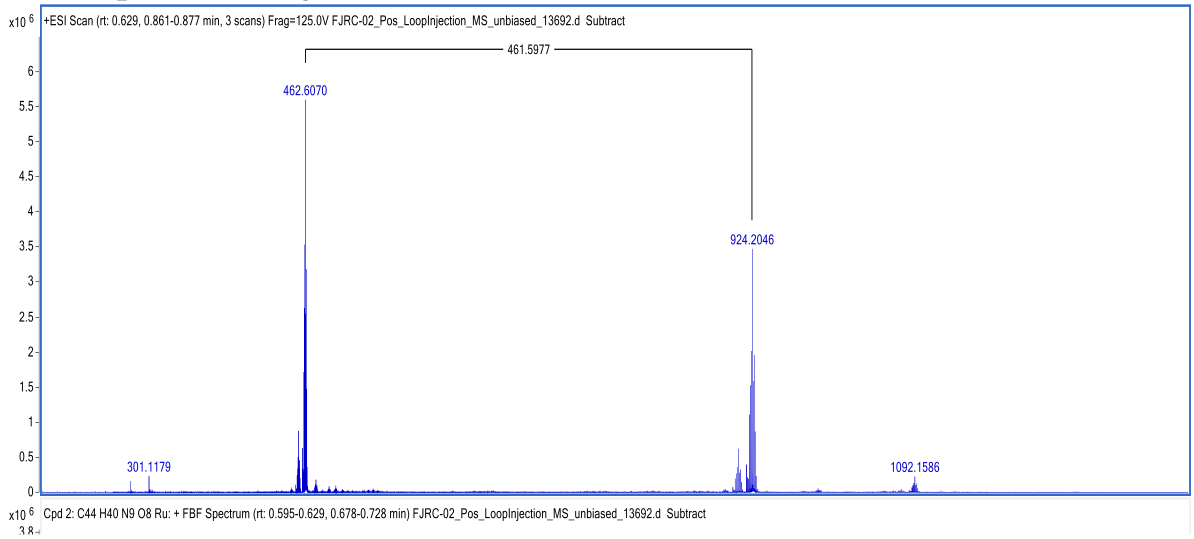

Figure S73. ESI-mass spectrum of **Ru** in acetonitrile. [Ru(dceb)$_2$(bpt)]$^{1+}$ calcd *m/z* = 924.205, found 924.2046.

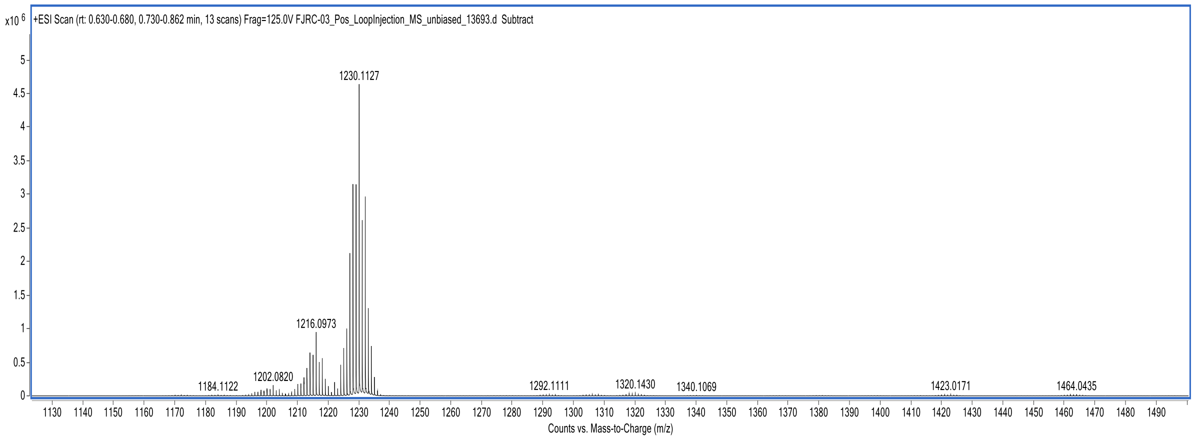

Figure S74. ESI mass spectrum of **RuRe** in acetonitrile. [Ru(dceb)$_2$(bpt)Re(CO)$_3$Cl]$^{1+}$ calcd. *m/z* 1230.114, found 1230.1127. [Ru(dceb)$_2$(bpt)Re(CO)$_3$Cl -CH$_2$] calcd *m/z* 1216.098 found 1216.0973.

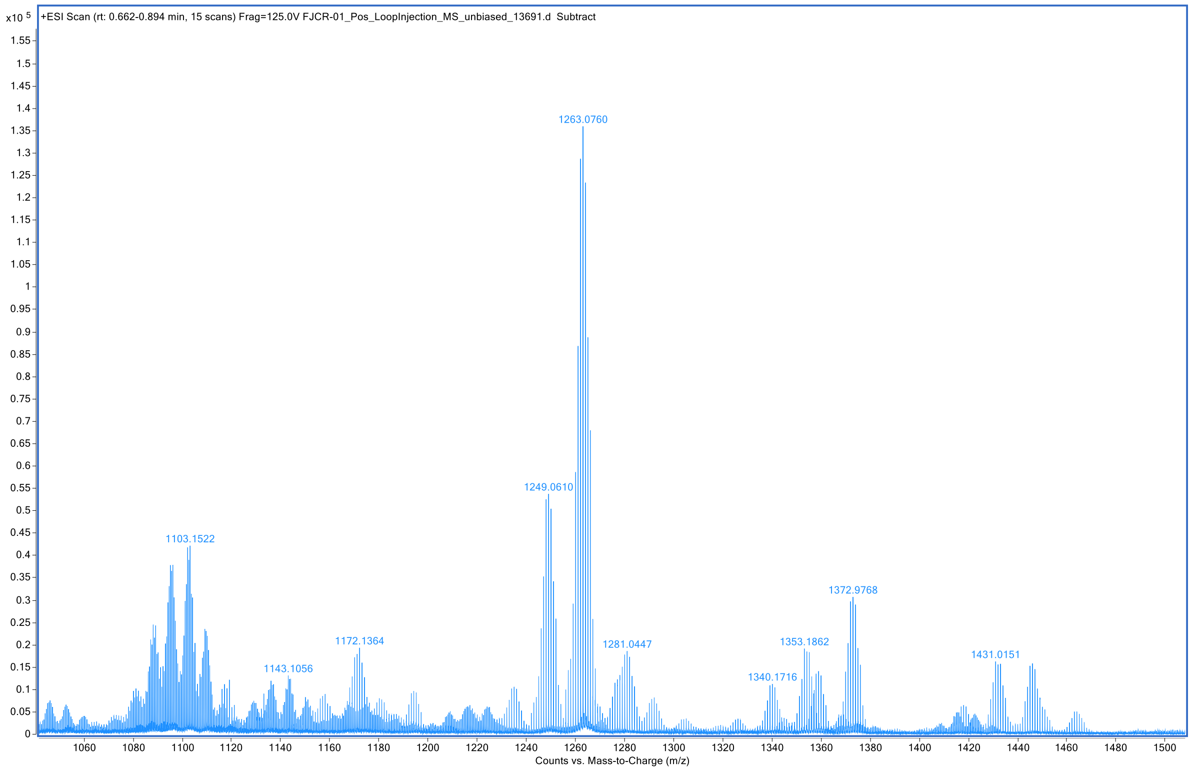

Figure S75. ESI mass spectrum of **RuPt** in acetonitrile. Ru(dceb)$_2$(bpt)Pt(H$_2$O)$_2$(PF$_6$)$_2$-CH$_2$]$^{1+}$ calcd. *m/z* 1431.103, found 1431.0151. [Ru(dceb)$_2$(bpt)PtI$_2$]$^{1+}$ calcd. *m/z* 1372.978, found 1372.9768. [Ru(dceb)$_2$(bpt)PtI(OH)(H$_2$O)] calcd. *m/z* 1281.087, found 1281.0447 [Ru(dceb)$_2$(bpt)PtI(OH)]$^{1+}$ calcd. *m/z* 1263.076, found 1263.076. [Ru(dceb)$_2$(bpt)PtI(OH)-CH$_2$]$^{1+}$ calcd. *m/z* 1249.061, found 1249.061.
